# Correction: SPACE: STRING proteins as complementary embeddings

**DOI:** 10.1093/bioinformatics/btag701

**Published:** 2026-09-30

This is a correction to: Dewei Hu, Damian Szklarczyk, Christian von Mering, Lars Juhl Jensen, SPACE: STRING proteins as complementary embeddings, Bioinformatics, Volume 41, Issue 9, September 2025, btaf496, https://doi.org/10.1093/bioinformatics/btaf496

The corresponding author on the paper has been changed to be Lars Juhl Jensen.

