# Supplementary material for: SPACE: STRING proteins as complementary embeddings
Source: Bioinformatics. 2025 Sep 9;41(9):btaf496. doi: 10.1093/bioinformatics/btaf496 (PMC12453690; doi:10.1093/bioinformatics/btaf496)
Supplement: btaf496_Supplementary_Data [file btaf496_supplementary_data.zip › SPACE_supp.pdf]

# Supplementary Materials of “SPACE: STRING proteins as complementary embeddings”

# 1 Hyperparameter Tuning

## 1.1 node2vec

We tuned the hyperparameters of node2vec in the following search spaces:

| **Hyperparameter** | **Search Space** | **Selection** |
|---|---|---|
| Dimensionality | 32, 64, 128, 256, 512 | 128 |
| Length of walk per source | 20, 50, 100 | 50 |
| Number of walks per source | 10, 20, 30 | 10 |
| Window size | 5, 10 | 5 |
| Epochs | 5, 10, 20 | 5 |
| Return and in-out hyperparameters | (0.1, 0.9), (0.3, 0.7), (0.7, 0.3), (0.9, 0.1) | (0.3,0.7) |

Suppl. Table 1: node2vec Hyperparameter Tuning Space

## 1.2 Link Prediction

Link prediction is a binary classification task that predicts whether a given unseen link exists in a network. In our networks, edges with scores higher than 0.7 were sampled as positive edges, while negative edges (not in the networks) were sampled based only on the training set. We maintained a positive-to-negative edge ratio of 1:10. To convert the node embeddings into edge vectors, we used several operators, and a logistic regression model was trained for the link prediction of each species.

| **Operator** | **Symbol** | **Definition** |
|---|---|---|
| Hadamard | $\odot$ | $[f(u) \odot f(v)]_i = f_i(u) \cdot f_i(v)$ |
| Weighted-L1 | $\Vert \cdot \Vert_{\bar{1}}$ | $\Vert f(u) - f(v) \Vert_{\bar{1}i} = \vert f_i(u) - f_i(v) \vert$ |
| Weighted-L2 | $\Vert \cdot \Vert_{\bar{2}}$ | $\Vert f(u) - f(v) \Vert_{\bar{2}i} = (f_i(u) - f_i(v))^2$ |

Suppl. Table 2: Operators for generating edge features in link prediction tasks. The table lists the operators used, their corresponding symbols, and their definitions. The Hadamard operator computes the element-wise product of two feature vectors. The Weighted-L1 and Weighted-L2 operators compute the L1 and L2 norms, respectively, weighted by the differences between corresponding feature vector elements.

## 1.3 Network Embedding Alignment

| **Hyperparameter** | **Search Space** | **Selection** |
|---|---|---|
| Distance function | Cosine distance, Euclidean distance | Euclidean distance |
| Margin | 1, 0.1, 0.01 | 0.1 |
| Balance hyperparameter | 0.1, 0.2, 0.3, 0.4, 0.5 | 0.5 |
| Latent dimensionality | 128, 256, 512, 1024 | 512 |

Suppl. Table 3: Seed Species Alignment Hyperparameter Tuning Space

# 2 Alignment quality assessment

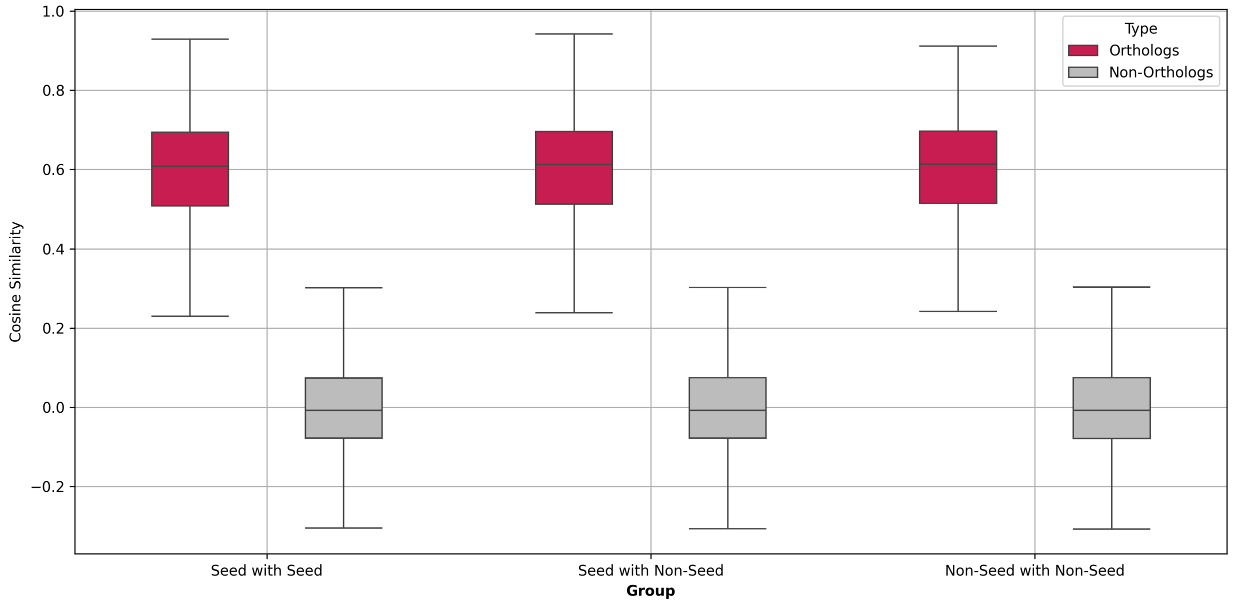


Suppl. Figure 1: Cosine similarity distributions between orthologous and non-orthologous protein pairs in aligned embedding space, in a single random sampling experiment.

| Group | N | Orthologs Median (IQR) | Non-orthologs Median (IQR) | Difference | Effect Size |
|---|---|---|---|---|---|
| Seed With Seed | 180023 | 0.61 (0.51 - 0.69) | -0.01 (-0.08 - 0.07) | 0.62 | -0.99 |
| Seed With Non-seed | 220888 | 0.61 (0.51 - 0.70) | -0.01 (-0.08 - 0.07) | 0.62 | -0.99 |
| Non-seed With Non-seed | 225552 | 0.61 (0.51 - 0.70) | -0.01 (-0.08 - 0.07) | 0.62 | -0.99 |

Suppl. Table 4: Statistical analysis of cosine similarities in different groups , in a single random sampling experiment. N is the number of sampled pairs, and the median is shown with 25% and 75% percentile. The p-values are less than 1e-300 (Mann–Whitney U test).

| Group | Type | Mean ± Std |
|---|---|---|
| Seed with Seed | Orthologs | 0.608 ± 0.0004 |
| Seed with Seed | Non-Orthologs | -0.008 ± 0.0003 |
| Seed with Non-Seed | Orthologs | 0.6131 ± 0.0005 |
| Seed with Non-Seed | Non-Orthologs | -0.0078 ± 0.0002 |
| Non-Seed with Non-Seed | Orthologs | 0.6133 ± 0.0003 |
| Non-Seed with Non-Seed | Non-Orthologs | -0.0078 ± 0.0002 |

Suppl. Table 5: Statistical analysis of cosine similarities over ten times repetitive random experiments. The mean and standard deviation values were calculated from the median of each single random sampling.

# 3 KEGG benchmark statistics

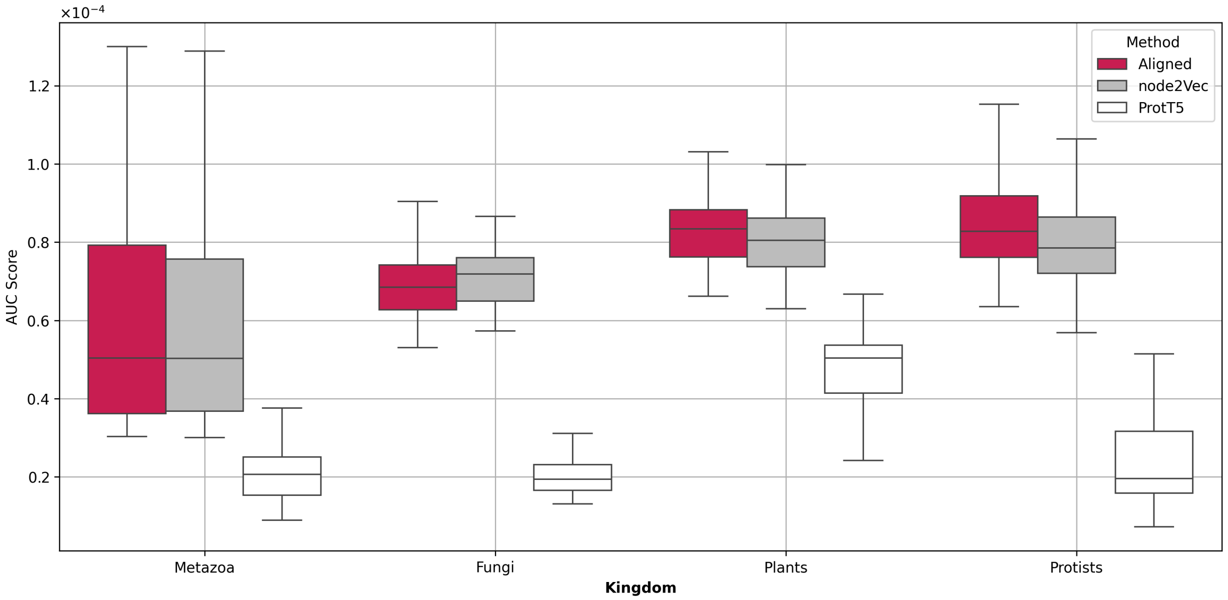


Suppl. Figure 2: Box plot of partial AUC scores (See Methods) distribution per kingdom.

| Method A | Method B | Kingdom | N | Ratio A/B Median (IQR) | p-value | Effect Size |
|---|---|---|---|---|---|---|
| Aligned | node2vec | Metazoa | 172 | 1.0 (0.99 - 1.02) | 1.33e-04 | 0.17 |
| Aligned | node2vec | Fungi | 101 | 0.96 (0.93 - 0.99) | 4.38e-09 | 0.54 |
| Aligned | node2vec | Plants | 67 | 1.03 (1.01 - 1.05) | 2.03e-08 | 0.64 |
| Aligned | node2vec | Protists | 38 | 1.06 (1.0 - 1.13) | 2.30e-06 | 0.53 |
| Aligned | ProtT5 | Metazoa | 172 | 2.48 (2.18 - 2.93) | 4.15e-29 | 0.99 |
| Aligned | ProtT5 | Fungi | 101 | 3.42 (3.02 - 4.06) | 2.67e-18 | 1.0 |
| Aligned | ProtT5 | Plants | 67 | 1.73 (1.58 - 1.87) | 1.12e-12 | 1.0 |
| Aligned | ProtT5 | Protists | 38 | 3.99 (2.71 - 4.85) | 7.28e-12 | 1.0 |
| node2vec | ProtT5 | Metazoa | 172 | 2.47 (2.19 - 2.91) | 3.68e-29 | 0.99 |
| node2vec | ProtT5 | Fungi | 101 | 3.56 (3.1 - 4.25) | 2.67e-18 | 1.0 |
| node2vec | ProtT5 | Plants | 67 | 1.7 (1.53 - 1.86) | 1.12e-12 | 1.0 |
| node2vec | ProtT5 | Protists | 38 | 3.81 (2.62 - 4.69) | 1.46e-11 | 0.95 |

Suppl. Table 6: Statistical comparison of embedding methods across taxonomic kingdoms in KEGG pathway prediction, and partial AUC scores are reported (see Methods). N is the number of species in that kingdom, and ratios are given by medians with 25% and 75% percentiles. P-values were calculated with the Wilcoxon test.

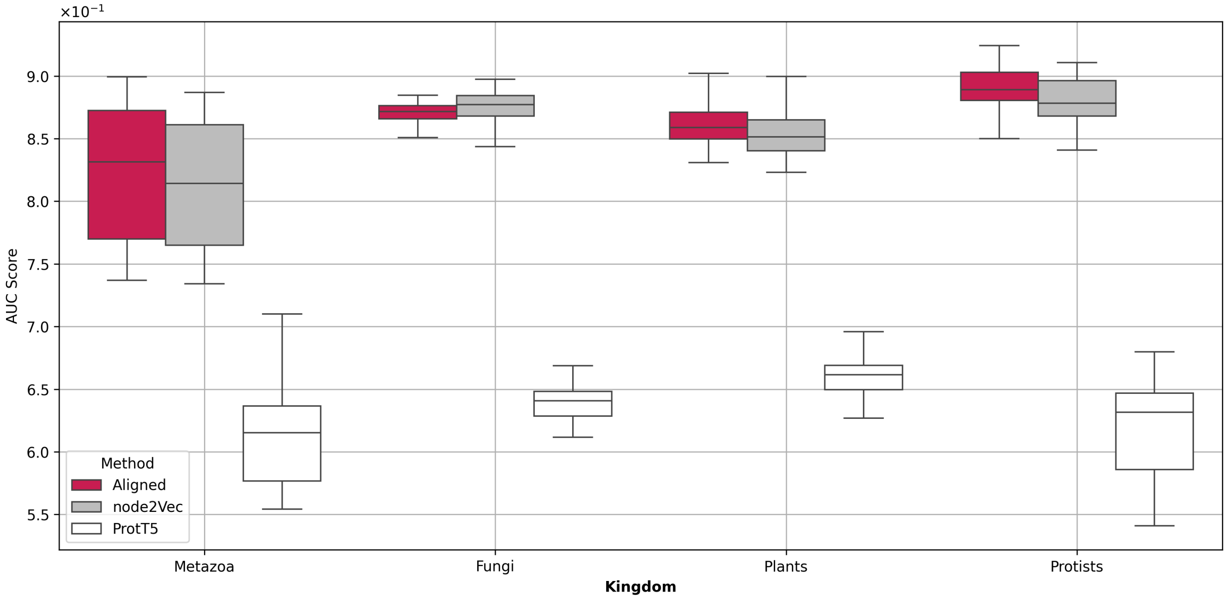


Suppl. Figure 3: Box plot of AUC scores distribution per kingdom.

| Method A | Method B | Kingdom | N | Ratio A/B Median (IQR) | p-value | Effect Size |
|---|---|---|---|---|---|---|
| Aligned | ProtT5 | Metazoa | 172 | 1.35 (1.33 - 1.36) | 5.62e-30 | 1.0 |
| Aligned | ProtT5 | Fungi | 101 | 1.35 (1.33 - 1.39) | 2.67e-18 | 1.0 |
| Aligned | ProtT5 | Plants | 67 | 1.29 (1.28 - 1.32) | 1.12e-12 | 1.0 |
| Aligned | ProtT5 | Protists | 38 | 1.4 (1.37 - 1.55) | 7.28e-12 | 1.0 |
| Aligned | node2vec | Metazoa | 172 | 1.01 (1.01 - 1.02) | 1.23e-29 | 0.93 |
| Aligned | node2vec | Fungi | 101 | 0.99 (0.99 - 1.0) | 7.72e-11 | 0.56 |
| Aligned | node2vec | Plants | 67 | 1.01 (1.0 - 1.01) | 1.39e-11 | 0.79 |
| Aligned | node2vec | Protists | 38 | 1.01 (1.01 - 1.02) | 1.51e-09 | 0.84 |
| node2vec | ProtT5 | Metazoa | 172 | 1.33 (1.31 - 1.35) | 5.62e-30 | 1.0 |
| node2vec | ProtT5 | Fungi | 101 | 1.37 (1.34 - 1.39) | 2.67e-18 | 1.0 |
| node2vec | ProtT5 | Plants | 67 | 1.28 (1.27 - 1.31) | 1.12e-12 | 1.0 |
| node2vec | ProtT5 | Protists | 38 | 1.38 (1.35 - 1.55) | 7.28e-12 | 1.0 |

Suppl. Table 7: Statistical comparison of embedding methods across taxonomic kingdoms in KEGG pathway prediction, and AUC scores are reported. N is the number of species in that kingdom, and ratios are given by medians with 25% and 75% percentiles. P-values were calculated with the Wilcoxon test.

# 4 Downstream Tasks Results

## 4.1 Subcellular Localization Prediction Scores

| | | ProtT5 | Aligned | SPACE |
|---|---|---|---|---|
| Accuracy | | $0.53 \pm 0.01$ | $0.44 \pm 0.02$ | $\mathbf{0.56 \pm 0.01}$ |
| Jaccard | | $0.55 \pm 0.02$ | $0.47 \pm 0.02$ | $\mathbf{0.58 \pm 0.02}$ |
| MicroF1 | | $0.71 \pm 0.02$ | $0.64 \pm 0.02$ | $\mathbf{0.74 \pm 0.01}$ |
| MacroF1 | | $0.52 \pm 0.00$ | $0.54 \pm 0.01$ | $\mathbf{0.65 \pm 0.01}$ |
| | | | | |
| MCC per location | count | | | |
| Nucleus | 9135 | $0.70 \pm 0.01$ | $0.66 \pm 0.01$ | $\mathbf{0.72 \pm 0.01}$ |
| Cytoplasm | 9108 | $0.59 \pm 0.02$ | $0.43 \pm 0.02$ | $\mathbf{0.60 \pm 0.01}$ |
| Cell membrane | 3930 | $0.65 \pm 0.02$ | $0.54 \pm 0.02$ | $\mathbf{0.66 \pm 0.01}$ |
| Mitochondrion | 2451 | $0.73 \pm 0.02$ | $0.67 \pm 0.02$ | $\mathbf{0.77 \pm 0.02}$ |
| Endoplasmic reticulum | 2021 | $0.45 \pm 0.03$ | $0.47 \pm 0.04$ | $\mathbf{0.56 \pm 0.04}$ |
| Extracellular | 1563 | $0.74 \pm 0.05$ | $0.53 \pm 0.03$ | $\mathbf{0.75 \pm 0.03}$ |
| Lysosome/Vacuole | 1424 | $0.07 \pm 0.06$ | $0.37 \pm 0.03$ | $\mathbf{0.38 \pm 0.03}$ |
| Golgi apparatus | 1209 | $0.27 \pm 0.06$ | $0.36 \pm 0.06$ | $\mathbf{0.41 \pm 0.07}$ |
| Plastid | 926 | $0.87 \pm 0.01$ | $0.67 \pm 0.03$ | $\mathbf{0.88 \pm 0.01}$ |
| Peroxisome | 272 | $0.06 \pm 0.09$ | $0.46 \pm 0.08$ | $\mathbf{0.52 \pm 0.04}$ |

Suppl. Table 8: **Protein subcellular localization prediction on DeepLoc 2.0 SwissProt cross-validation dataset.** The best scores for each metric are shown in bold. SPACE: the concatenation of ProtT5 and aligned network embeddings. A logistic regression model was trained on each location with the corresponding embeddings. The scores are reported in the mean score with standard deviation on 5 partitions.

| | | DeepLoc 2.0 | ProtT5 | Aligned | SPACE |
|---|---|---|---|---|---|
| Accuracy | | 0.38 | 0.51 | 0.48 | **0.53** |
| Jaccard | | 0.42 | 0.50 | 0.50 | **0.53** |
| Micro F1 | | 0.59 | 0.66 | 0.66 | **0.70** |
| Macro F1 | | 0.45 | 0.48 | 0.56 | **0.61** |
| | | | | | |
| MCC per location | count | | | | |
| Nucleus | 865 | 0.44 | 0.58 | 0.54 | **0.60** |
| Cytoplasm | 518 | 0.34 | **0.47** | 0.46 | 0.46 |
| Cell membrane | 271 | 0.35 | 0.46 | 0.53 | **0.57** |
| Mitochondrion | 183 | 0.47 | 0.71 | 0.72 | **0.77** |
| Golgi apparatus | 85 | 0.24 | 0.16 | 0.31 | **0.44** |
| Endoplasmic reticulum | 74 | 0.20 | 0.19 | 0.41 | **0.45** |

Suppl. Table 9: **Protein subcellular localization prediction on DeepLoc 2.0 HPA test set.** The best scores for each metric are shown in bold. SPACE is the concatenation of ProtT5 and aligned network embeddings. A logistic regression model was trained on each location with the corresponding embeddings and cross-validation dataset.

## 4.2 Protein Similarity in Subcellular Localizations

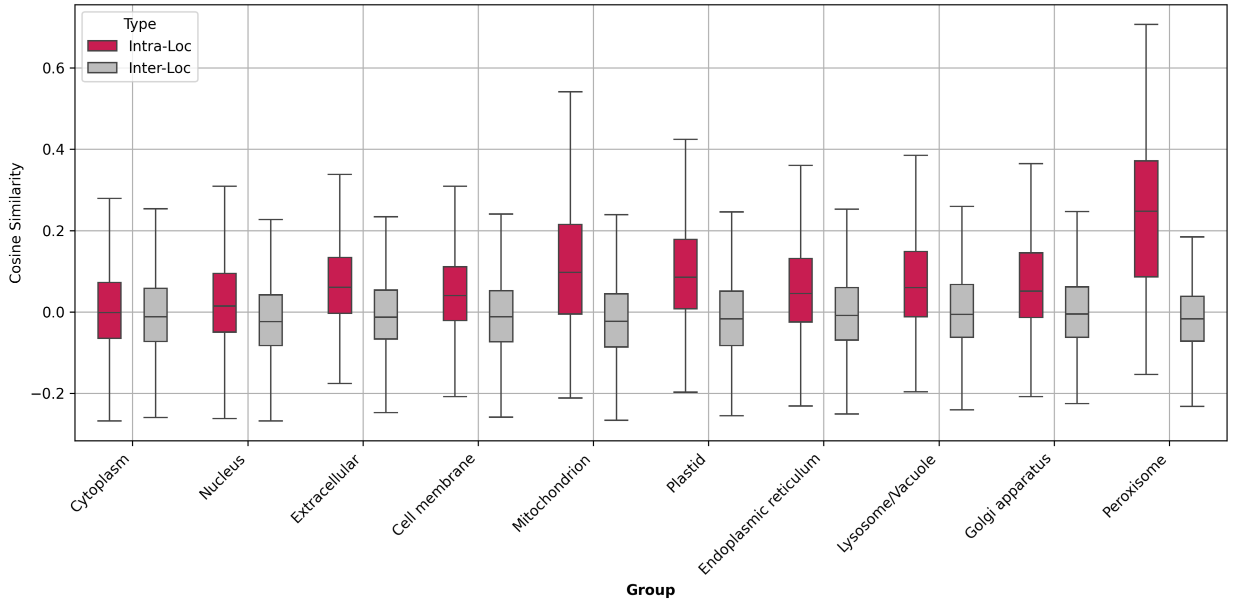


Suppl. Figure 4: Protein cosine similarity distributions per localization. Intra-Loc: proteins within the same localizations; Inter-Loc: proteins in different localizations.

| Location | N | Intra-Loc (IQR) | Inter-Loc (IQR) | Difference | p-value | Effect Size |
|---|---|---|---|---|---|---|
| Cytoplasm | 6835 | -0.00 (-0.06 - 0.07) | -0.01 (-0.07 - 0.06) | 0.01 | 3.14e-12 | -0.07 |
| Nucleus | 6836 | 0.02 (-0.05 - 0.09) | -0.02 (-0.08 - 0.04) | 0.04 | 2.74e-123 | -0.23 |
| Extracellular | 1170 | 0.06 (0.00 - 0.13) | -0.01 (-0.07 - 0.05) | 0.07 | 4.36e-70 | -0.42 |
| Cell membrane | 2950 | 0.04 (-0.02 - 0.11) | -0.01 (-0.07 - 0.05) | 0.05 | 4.63e-98 | -0.32 |
| Mitochondrion | 1829 | 0.09 (-0.00 - 0.21) | -0.03 (-0.09 - 0.05) | 0.12 | 6.31e-143 | -0.49 |
| Plastid | 683 | 0.08 (0.00 - 0.18) | -0.02 (-0.08 - 0.04) | 0.10 | 3.61e-54 | -0.48 |
| Endoplasmic reticulum | 1515 | 0.04 (-0.03 - 0.13) | -0.01 (-0.07 - 0.05) | 0.05 | 1.79e-42 | -0.29 |
| Lysosome/Vacuole | 1069 | 0.06 (-0.02 - 0.16) | -0.00 (-0.06 - 0.06) | 0.07 | 4.21e-44 | -0.35 |
| Golgi apparatus | 898 | 0.05 (-0.01 - 0.14) | -0.01 (-0.07 - 0.06) | 0.06 | 4.20e-33 | -0.33 |
| Peroxisome | 204 | 0.21 (0.08 - 0.35) | -0.02 (-0.08 - 0.06) | 0.23 | 2.37e-34 | -0.70 |

Suppl. Table 10: Statistical analysis of protein cosine similarities in different localizations. N is the number of sampled protein pairs, and the median values are given in columns Intra–Loc and Inter–Loc with 25% and 75% quantiles. Difference is the difference between median values. P-values were calculated using Mann–Whitney U test.

## 4.3 Protein Function Prediction

| | $F_{max}$ | | | AUPRC | | | $S_{min}$ | | |
|---|---|---|---|---|---|---|---|---|---|
| | MF | BP | CC | MF | BP | CC | MF | BP | CC |
| ProtT5 | 0.64 | 0.30 | 0.63 | **0.63** | 0.17 | 0.61 | 5.78 | **33.08** | 6.72 |
| Aligned | 0.64 | 0.33 | 0.61 | 0.56 | 0.19 | 0.55 | 5.37 | 36.81 | 7.44 |
| SPACE | **0.65** | **0.36** | **0.65** | 0.62 | **0.23** | **0.63** | **4.99** | 33.36 | **6.36** |

Suppl. Table 11: **Protein function prediction metrics on NetGO 2.0 test set.** The best scores for each metric are shown in bold. SPACE is the concatenation of ProtT5 and aligned network embeddings. A logistic regression model was trained on each GO term with the corresponding embeddings.

# 5 Network Statistics

Suppl. Table 12: Network statistics for 1,322 eukaryotes used in SPACE

| Name | Taxon ID | Kingdom | Seed | Number of Nodes | Number of Edges |
|---|---|---|---|---|---|
| Aedes aegypti | 7159 | metazoa | True | 13929 | 2023847 |
| Amborella trichopoda | 13333 | plant | True | 17996 | 2546231 |
| Apis mellifera | 7460 | metazoa | True | 11200 | 1741032 |
| Arabidopsis thaliana | 3702 | plant | True | 25739 | 6278747 |
| Aspergillus fumigatus | 330879 | fungi | True | 8948 | 1037248 |
| Branchiostoma floridae | 7739 | metazoa | True | 22811 | 2656434 |
| Caenorhabditis elegans | 6239 | metazoa | True | 18500 | 4635938 |
| Candida albicans | 237561 | fungi | True | 5984 | 1017304 |
| Canis lupus familiaris | 9615 | metazoa | True | 19921 | 5384258 |
| Chlamydomonas reinhardtii | 3055 | plant | True | 11452 | 2235926 |
| Citrus sinensis | 2711 | plant | True | 25592 | 6828153 |
| Cordyceps militaris | 983644 | fungi | True | 8484 | 1173991 |
| Cryptococcus neoformans | 214684 | fungi | True | 6047 | 1033028 |
| Danio rerio | 7955 | metazoa | True | 24474 | 6907601 |
| Dictyostelium discoideum | 44689 | protists | True | 8498 | 1675783 |
| Drosophila melanogaster | 7227 | metazoa | True | 13577 | 2516980 |
| Galdieria sulphuraria | 130081 | protists | True | 4663 | 782570 |
| Gallus gallus | 9031 | metazoa | True | 16538 | 3354533 |
| Gloeophyllum trabeum | 670483 | fungi | True | 9721 | 1359234 |
| Glycine max | 3847 | plant | True | 47662 | 19372483 |
| Homo sapiens | 9606 | metazoa | True | 19622 | 6857702 |
| Komagataella phaffii | 644223 | fungi | True | 4644 | 699972 |
| Malassezia restricta | 425264 | fungi | True | 3875 | 544850 |
| Mizuhopecten yessoensis | 6573 | metazoa | True | 16743 | 2254875 |
| Mus musculus | 10090 | metazoa | True | 21645 | 6342177 |
| Nelumbo nucifera | 4432 | plant | True | 22438 | 4568410 |
| Nematostella vectensis | 45351 | metazoa | True | 20291 | 3764439 |
| Neurospora crassa | 367110 | fungi | True | 8419 | 1075968 |
| Oryzias latipes | 8090 | metazoa | True | 23045 | 7567823 |
| Ostreococcus tauri | 70448 | plant | True | 6086 | 1064524 |
| Paracoccidioides brasiliensis | 502780 | fungi | True | 6852 | 1103432 |
| Physcomitrella patens | 3218 | plant | True | 19032 | 5566726 |
| Plasmodium falciparum | 36329 | protists | True | 3903 | 695804 |
| Pomacea canaliculata | 400727 | metazoa | True | 14714 | 2231226 |
| Populus trichocarpa | 3694 | plant | True | 35568 | 13871124 |
| Saccharomyces cerevisiae | 4932 | fungi | True | 6538 | 1412421 |
| Salmo salar | 8030 | metazoa | True | 45765 | 36392177 |
| Schizosaccharomyces pombe | 284812 | fungi | True | 5063 | 817409 |
| Selaginella moellendorffii | 88036 | plant | True | 26384 | 5744938 |
| Solanum tuberosum | 4113 | plant | True | 27155 | 6033833 |
| Spinacia oleracea | 3562 | plant | True | 17622 | 3070650 |
| Sus scrofa | 9823 | metazoa | True | 20244 | 4532586 |
| Thalassiosira pseudonana | 35128 | protists | True | 8566 | 1390527 |
| Trichoplax adhaerens | 10228 | metazoa | True | 9933 | 1376785 |
| Trypanosoma brucei | 185431 | protists | True | 5167 | 1007040 |
| Wallemia ichthyophaga | 1299270 | fungi | True | 4152 | 631442 |

Continued on next page

**Table 12 – continued from previous page**

| Name | Taxon ID | Kingdom | Seed | Number of Nodes | Number of Edges |
|---|---|---|---|---|---|
| Zea mays | 4577 | plant | True | 34009 | 11637187 |
| Zymoseptoria tritici IPO323 | 336722 | fungi | True | 8690 | 944388 |
| Absidia glauca | 4829 | fungi | False | 11202 | 5460879 |
| Absidia repens | 90262 | fungi | False | 11688 | 6624212 |
| Acanthamoeba castellanii | 1257118 | protists | False | 9388 | 2181227 |
| Acanthocheilonema viteae | 6277 | metazoa | False | 8646 | 4124122 |
| Acanthochromis polyacanthus | 80966 | metazoa | False | 23723 | 25711648 |
| Acaromyces ingoldii | 215250 | fungi | False | 6404 | 1997087 |
| Accipiter nisus | 211598 | metazoa | False | 16947 | 12270409 |
| Achlya hypogyna | 1202772 | protists | False | 10573 | 5913136 |
| Acidomyces richmondensis | 766039 | fungi | False | 8675 | 4094777 |
| Acremonium chrysogenum | 857340 | fungi | False | 7958 | 2887370 |
| Acromyrmex echinatior | 103372 | metazoa | False | 10653 | 1519961 |
| Actinidia chinensis | 1590841 | plant | False | 31821 | 35263247 |
| Acyrthosiphon pisum | 7029 | metazoa | False | 22431 | 3791799 |
| Aedes albopictus | 7160 | metazoa | False | 14440 | 2869194 |
| Aegilops tauschii | 200361 | plant | False | 33399 | 44708031 |
| Agaricus bisporus | 597362 | fungi | False | 8192 | 1586495 |
| Agrilus planipennis | 224129 | metazoa | False | 7624 | 2822309 |
| Ailuropoda melanoleuca | 9646 | metazoa | False | 19123 | 6185917 |
| Albugo candida | 65357 | protists | False | 8341 | 3216755 |
| Alligator mississippiensis | 8496 | metazoa | False | 18768 | 4013778 |
| Alligator sinensis | 38654 | metazoa | False | 18787 | 4492802 |
| Allomyces macrogynus | 578462 | fungi | False | 12541 | 8958199 |
| Alternaria alternata | 5599 | fungi | False | 10839 | 1113410 |
| Amanita muscaria | 946122 | fungi | False | 11245 | 6727189 |
| Amanita thiersii | 703135 | fungi | False | 7834 | 3319830 |
| Amazona aestiva | 12930 | metazoa | False | 14819 | 9300448 |
| Amazona collaria | 241587 | metazoa | False | 16346 | 12590774 |
| Amorphotheca resinae | 857342 | fungi | False | 7870 | 3432283 |
| Amphilophus citrinellus | 61819 | metazoa | False | 23345 | 23477760 |
| Amphimedon queenslandica | 400682 | metazoa | False | 21850 | 5018033 |
| Amphiprion ocellaris | 80972 | metazoa | False | 23256 | 7658057 |
| Amphiprion percula | 161767 | metazoa | False | 23535 | 23569525 |
| Anabas testudineus | 64144 | metazoa | False | 24252 | 23221958 |
| Anaeromyces robustus | 1754192 | fungi | False | 8815 | 4035862 |
| Ananas comosus | 4615 | plant | False | 21788 | 11277551 |
| Anas platyrhynchos | 8840 | metazoa | False | 16285 | 3800981 |
| Ancylostoma caninum | 29170 | metazoa | False | 22835 | 25885794 |
| Ancylostoma ceylanicum | 53326 | metazoa | False | 18120 | 10042342 |
| Angiostrongylus cantonensis | 6313 | metazoa | False | 11321 | 4625255 |
| Angiostrongylus costaricensis | 334426 | metazoa | False | 10437 | 3660205 |
| Anncaliia algerae | 1288291 | fungi | False | 1572 | 207334 |
| Anolis carolinensis | 28377 | metazoa | False | 18180 | 3950065 |
| Anopheles albimanus | 7167 | metazoa | False | 10328 | 4845215 |
| Anopheles atroparvus | 41427 | metazoa | False | 11039 | 5808315 |
| Anopheles christyi | 43041 | metazoa | False | 9765 | 4499611 |
| Anopheles coluzzii | 1518534 | metazoa | False | 11460 | 5043869 |
| Anopheles culicifacies | 139723 | metazoa | False | 12032 | 6515917 |

Continued on next page

**Table 12 – continued from previous page**

| Name | Taxon ID | Kingdom | Seed | Number of Nodes | Number of Edges |
|---|---|---|---|---|---|
| Anopheles darlingi | 43151 | metazoa | False | 9658 | 4158255 |
| Anopheles dirus | 7168 | metazoa | False | 11202 | 6253948 |
| Anopheles epiroticus | 199890 | metazoa | False | 11093 | 6030049 |
| Anopheles farauti | 69004 | metazoa | False | 10746 | 5214207 |
| Anopheles funestus | 62324 | metazoa | False | 12609 | 6794343 |
| Anopheles gambiae | 7165 | metazoa | False | 11567 | 1548366 |
| Anopheles melas | 34690 | metazoa | False | 12891 | 7180960 |
| Anopheles merus | 30066 | metazoa | False | 10815 | 5501441 |
| Anopheles minimus | 112268 | metazoa | False | 10925 | 5632383 |
| Anopheles quadriannulatus | 34691 | metazoa | False | 11547 | 6407629 |
| Anopheles sinensis | 74873 | metazoa | False | 11435 | 5904543 |
| Anopheles stephensi | 30069 | metazoa | False | 10934 | 6278579 |
| Anser brachyrhynchus | 132585 | metazoa | False | 15273 | 9334234 |
| Anser cygnoides | 8845 | metazoa | False | 15231 | 3231593 |
| Aotus nancymaae | 37293 | metazoa | False | 19877 | 5143340 |
| Aphanomyces astaci | 112090 | protists | False | 11482 | 6236165 |
| Aphanomyces invadans | 157072 | protists | False | 10025 | 4887483 |
| Apiotrichum porosum | 105984 | fungi | False | 7030 | 2516912 |
| Apis cerana | 94128 | metazoa | False | 9179 | 3710480 |
| Apostasia shenzhenica | 1088818 | plant | False | 17193 | 8456727 |
| Apostichopus japonicus | 307972 | metazoa | False | 23702 | 19788639 |
| Aptenodytes forsteri | 9233 | metazoa | False | 12958 | 5761300 |
| Apteryx haastii | 8823 | metazoa | False | 16374 | 11080754 |
| Apteryx owenii | 8824 | metazoa | False | 15869 | 10571374 |
| Apteryx rowi | 308060 | metazoa | False | 15969 | 11092716 |
| Aquila chrysaetos | 223781 | metazoa | False | 15857 | 10344685 |
| Aquilegia coerulea | 218851 | plant | False | 21840 | 10259573 |
| Arabidopsis lyrata | 81972 | plant | False | 27744 | 9201009 |
| Arachis hypogaea | 3818 | plant | False | 54351 | 86868448 |
| Armadillidium vulgare | 13347 | metazoa | False | 12434 | 7440096 |
| Armillaria gallica | 47427 | fungi | False | 15814 | 14155625 |
| Armillaria ostoyae | 47428 | fungi | False | 14976 | 7615848 |
| Armillaria solidipes | 1076256 | fungi | False | 14462 | 9922546 |
| Artemisia annua | 35608 | plant | False | 51327 | 74761730 |
| Arthrobotrys flagrans | 97331 | fungi | False | 7598 | 3233878 |
| Arthrobotrys oligospora | 756982 | fungi | False | 8348 | 3675491 |
| Asbolus verrucosus | 1661398 | metazoa | False | 12631 | 3215462 |
| Ascobolus immersus | 1160509 | fungi | False | 8090 | 4351851 |
| Ascochyta rabiei | 5454 | fungi | False | 9071 | 3319632 |
| Ascodesmis nigricans | 341454 | fungi | False | 6587 | 2783568 |
| Ascoidea rubescens | 1344418 | fungi | False | 5179 | 1615120 |
| Aspergillus aculeatus | 690307 | fungi | False | 10059 | 4139246 |
| Aspergillus arachidicola | 656916 | fungi | False | 11220 | 5880615 |
| Aspergillus awamori | 105351 | fungi | False | 10282 | 3992439 |
| Aspergillus bombycis | 109264 | fungi | False | 11524 | 5306688 |
| Aspergillus brasiliensis | 767769 | fungi | False | 10991 | 4950272 |
| Aspergillus calidoustus | 454130 | fungi | False | 13451 | 7893165 |
| Aspergillus candidus | 41067 | fungi | False | 8414 | 3231832 |
| Aspergillus carbonarius | 602072 | fungi | False | 9897 | 4323032 |

Continued on next page

Table 12 – continued from previous page

| Name | Taxon ID | Kingdom | Seed | Number of Nodes | Number of Edges |
|---|---|---|---|---|---|
| Aspergillus clavatus | 344612 | fungi | False | 8510 | 1124184 |
| Aspergillus cristatus | 573508 | fungi | False | 9197 | 4556900 |
| Aspergillus ellipticus | 1448320 | fungi | False | 10517 | 5180152 |
| Aspergillus fischeri | 331117 | fungi | False | 9713 | 1184160 |
| Aspergillus flavus | 332952 | fungi | False | 11704 | 1316544 |
| Aspergillus glaucus | 1160497 | fungi | False | 9566 | 4970440 |
| Aspergillus heteromorphus | 1448321 | fungi | False | 9097 | 3836087 |
| Aspergillus homomorphus | 1450537 | fungi | False | 9788 | 3826775 |
| Aspergillus ibericus | 1448316 | fungi | False | 10138 | 4237299 |
| Aspergillus kawachii | 1033177 | fungi | False | 10573 | 4552633 |
| Aspergillus lentulus | 293939 | fungi | False | 9125 | 4015083 |
| Aspergillus mulundensis | 1810919 | fungi | False | 10327 | 4888195 |
| Aspergillus nidulans | 227321 | fungi | False | 9607 | 1146659 |
| Aspergillus niger | 380704 | fungi | False | 10146 | 4480273 |
| Aspergillus nomius | 1509407 | fungi | False | 11157 | 5379558 |
| Aspergillus novofumigatus | 1392255 | fungi | False | 10100 | 5228127 |
| Aspergillus oryzae | 510516 | fungi | False | 11014 | 1174765 |
| Aspergillus parasiticus | 1403190 | fungi | False | 8167 | 3272188 |
| Aspergillus rambellii | 308745 | fungi | False | 7503 | 2507161 |
| Aspergillus ruber | 1388766 | fungi | False | 8818 | 3891452 |
| Aspergillus saccharolyticus | 1450539 | fungi | False | 8774 | 3306564 |
| Aspergillus sclerotialis | 2070753 | fungi | False | 10183 | 4338995 |
| Aspergillus sclerotiicarbonarius | 1448318 | fungi | False | 10868 | 5041666 |
| Aspergillus sclerotioniger | 1450535 | fungi | False | 10636 | 4862902 |
| Aspergillus steynii | 1392250 | fungi | False | 11199 | 5140317 |
| Aspergillus sydowii | 1036612 | fungi | False | 11809 | 5591936 |
| Aspergillus tanneri | 1220188 | fungi | False | 10684 | 5132178 |
| Aspergillus terreus | 341663 | fungi | False | 9653 | 3988211 |
| Aspergillus thermomutatus | 41047 | fungi | False | 9115 | 4216221 |
| Aspergillus tubingensis | 767770 | fungi | False | 10485 | 4282599 |
| Aspergillus turcosus | 1245748 | fungi | False | 8613 | 3657958 |
| Aspergillus udagawae | 91492 | fungi | False | 9417 | 4226953 |
| Aspergillus uvarum | 1448315 | fungi | False | 10361 | 4853562 |
| Aspergillus versicolor | 1036611 | fungi | False | 11747 | 5230273 |
| Aspergillus violaceofuscus | 1450538 | fungi | False | 10320 | 4710331 |
| Aspergillus welwitschiae | 1341132 | fungi | False | 10792 | 4014622 |
| Aspergillus wentii | 1073089 | fungi | False | 10757 | 5463759 |
| Astatotilapia calliptera | 8154 | metazoa | False | 26594 | 28224796 |
| Astyanax mexicanus | 7994 | metazoa | False | 25966 | 9109103 |
| Athene cunicularia | 194338 | metazoa | False | 13560 | 2888246 |
| Atta cephalotes | 12957 | metazoa | False | 9948 | 1224074 |
| Atta colombica | 520822 | metazoa | False | 9805 | 4393270 |
| Aureobasidium melanogenum | 1043003 | fungi | False | 8944 | 3621164 |
| Aureobasidium namibiae | 1043004 | fungi | False | 8833 | 4204070 |
| Aureobasidium pullulans | 1043002 | fungi | False | 9304 | 3944106 |
| Aureobasidium subglaciale | 1043005 | fungi | False | 8913 | 4100309 |
| Auriculariopsis ampla | 97359 | fungi | False | 10507 | 3853767 |
| Austrofundulus limnaeus | 52670 | metazoa | False | 23236 | 7837282 |
| Auxenochlorella protothecoides | 3075 | plant | False | 5461 | 806725 |

Continued on next page

**Table 12 – continued from previous page**

| Name | Taxon ID | Kingdom | Seed | Number of Nodes | Number of Edges |
|---|---|---|---|---|---|
| Babesia bigemina | 5866 | protists | False | 2573 | 704152 |
| Babesia bovis | 5865 | protists | False | 2420 | 578169 |
| Babjeviella inositovora | 984486 | fungi | False | 5208 | 1479682 |
| Balaenoptera acutorostrata | 310752 | metazoa | False | 18576 | 5729300 |
| Basidiobolus meristosporus | 1314790 | fungi | False | 11971 | 7460656 |
| Bathycoccus prasinos | 41875 | plant | False | 6064 | 1127035 |
| Batrachochytrium dendrobatidis JAM81 | 684364 | fungi | False | 6424 | 2298845 |
| Batrachochytrium dendrobatidis JEL423 | 403673 | fungi | False | 6328 | 2557435 |
| Batrachochytrium salamandrivorans | 1357716 | fungi | False | 6931 | 2716366 |
| Baudoinia panamericana | 717646 | fungi | False | 7947 | 907682 |
| Beauveria bassiana ARSEF2860 | 655819 | fungi | False | 9308 | 4116416 |
| Beauveria bassiana D15 | 1245745 | fungi | False | 10674 | 4554664 |
| Besnoitia besnoiti | 94643 | protists | False | 4740 | 1478324 |
| Betta splendens | 158456 | metazoa | False | 21860 | 20565673 |
| Biomphalaria glabrata | 6526 | metazoa | False | 19236 | 7195856 |
| Bipolaris maydis | 701091 | fungi | False | 10225 | 1907070 |
| Bipolaris oryzae | 930090 | fungi | False | 9625 | 1158270 |
| Bipolaris sorokiniana | 665912 | fungi | False | 9861 | 1113107 |
| Bipolaris zeicola | 930089 | fungi | False | 9763 | 1146916 |
| Bison bison | 43346 | metazoa | False | 19881 | 5734820 |
| Blastocystis sp. ATCC 50177Nand | 478820 | protists | False | 4638 | 1874777 |
| Blastomyces dermatitidis | 559297 | fungi | False | 7516 | 3377035 |
| Blastomyces gilchristii | 559298 | fungi | False | 7623 | 1114758 |
| Blastomyces parvus | 2060905 | fungi | False | 7465 | 3083321 |
| Blastomyces percursus | 1658174 | fungi | False | 8585 | 4494788 |
| Blastomyces silverae | 2060906 | fungi | False | 6997 | 3077314 |
| Blattella germanica | 6973 | metazoa | False | 16144 | 10765518 |
| Blumeria graminis | 546991 | fungi | False | 5444 | 1847202 |
| Bombyx mori | 7091 | metazoa | False | 11681 | 1718650 |
| Bos grunniens | 30521 | metazoa | False | 20673 | 5068119 |
| Bos indicus | 30522 | metazoa | False | 21081 | 5471806 |
| Bos mutus | 72004 | metazoa | False | 20501 | 6840640 |
| Bos taurus | 9913 | metazoa | False | 21531 | 6608569 |
| Botryobasidium botryosum | 930990 | fungi | False | 11265 | 7484574 |
| Botryotinia calthae | 38488 | fungi | False | 9697 | 4618958 |
| Botryotinia narcissicola | 278944 | fungi | False | 9671 | 4601103 |
| Botrytis cinerea BcDW1 | 1290391 | fungi | False | 9370 | 4678360 |
| Botrytis cinerea T4 | 999810 | fungi | False | 10037 | 4745044 |
| Botrytis elliptica | 278938 | fungi | False | 9834 | 4648621 |
| Botrytis porri | 87229 | fungi | False | 9337 | 4357055 |
| Brachionus plicatilis | 10195 | metazoa | False | 12817 | 7156704 |
| Brachypodium distachyon | 15368 | plant | False | 25821 | 7627285 |
| Brassica napus | 3708 | plant | False | 50575 | 31596341 |
| Brassica oleracea | 109376 | plant | False | 49201 | 25711098 |
| Brassica rapa | 51351 | plant | False | 37331 | 17771374 |
| Bremia lactucae | 4779 | protists | False | 6265 | 1958791 |
| Brettanomyces bruxellensis | 5007 | fungi | False | 4140 | 1200692 |
| Brettanomyces naardenensis | 13370 | fungi | False | 4761 | 1340752 |
| Brugia malayi | 6279 | metazoa | False | 6599 | 1447349 |

Continued on next page

**Table 12 – continued from previous page**

| Name | Taxon ID | Kingdom | Seed | Number of Nodes | Number of Edges |
|---|---|---|---|---|---|
| Brugia pahangi | 6280 | metazoa | False | 11408 | 6762255 |
| Brugia timori | 42155 | metazoa | False | 12083 | 7502791 |
| Byssochlamys spectabilis | 264951 | fungi | False | 8418 | 3490377 |
| Cadophora sp. DSE1049 | 1485229 | fungi | False | 16946 | 8948919 |
| Caenorhabditis brenneri | 135651 | metazoa | False | 20866 | 20123665 |
| Caenorhabditis briggsae | 6238 | metazoa | False | 15771 | 4885892 |
| Caenorhabditis japonica | 281687 | metazoa | False | 21217 | 18446741 |
| Caenorhabditis latens | 1503980 | metazoa | False | 15927 | 10653811 |
| Caenorhabditis nigoni | 1611254 | metazoa | False | 18962 | 15823998 |
| Caenorhabditis remanei | 31234 | metazoa | False | 21488 | 25723434 |
| Caenorhabditis tropicalis | 1561998 | metazoa | False | 19687 | 17968078 |
| Cajanus cajan | 3821 | plant | False | 37993 | 9515062 |
| Calidris pugnax | 198806 | metazoa | False | 16036 | 10734463 |
| Calidris pygmaea | 425635 | metazoa | False | 16038 | 10910930 |
| Callipepla squamata | 9009 | metazoa | False | 16744 | 10321884 |
| Callithrix jacchus | 9483 | metazoa | False | 21624 | 8201935 |
| Callorhinchus milii | 7868 | metazoa | False | 18781 | 4380154 |
| Calocera cornea | 1353952 | fungi | False | 9150 | 4995275 |
| Calocera viscosa | 1330018 | fungi | False | 9205 | 4528409 |
| Calypte anna | 9244 | metazoa | False | 12723 | 6712548 |
| Camellia sinensis | 542762 | plant | False | 27695 | 21677408 |
| Camelus dromedarius | 9838 | metazoa | False | 19034 | 5733182 |
| Camponotus floridanus | 104421 | metazoa | False | 11610 | 1552212 |
| Candida arabinofermentans | 983967 | fungi | False | 4909 | 1245199 |
| Candida auris | 498019 | fungi | False | 5035 | 694834 |
| Candida glabrata | 284593 | fungi | False | 5073 | 990616 |
| Candida haemulonis | 45357 | fungi | False | 4796 | 1175551 |
| Candida inconspicua | 52247 | fungi | False | 4480 | 1092071 |
| Candida intermedia | 45354 | fungi | False | 5363 | 1546329 |
| Candida maltosa | 1245528 | fungi | False | 5399 | 1478605 |
| Candida parapsilosis | 578454 | fungi | False | 5378 | 1449020 |
| Candida pseudohaemulonis | 418784 | fungi | False | 4680 | 1118049 |
| Candida tropicalis | 294747 | fungi | False | 5666 | 823789 |
| Candida viswanathii | 5486 | fungi | False | 9583 | 4587517 |
| Canis lupus dingo | 286419 | metazoa | False | 21021 | 6801434 |
| Capitella teleta | 283909 | metazoa | False | 21560 | 16182951 |
| Capra hircus | 9925 | metazoa | False | 21151 | 8316810 |
| Capronia coronata | 1182541 | fungi | False | 8491 | 3735740 |
| Capronia epimyces | 1182542 | fungi | False | 9484 | 4114272 |
| Capsaspora owczarzaki | 595528 | other | False | 6803 | 2060415 |
| Capsella rubella | 81985 | plant | False | 25146 | 7072184 |
| Capsicum annuum | 4072 | plant | False | 33034 | 10547035 |
| Capsicum baccatum | 33114 | plant | False | 32303 | 10310181 |
| Capsicum chinense | 80379 | plant | False | 31838 | 10412139 |
| Carlito syrichta | 1868482 | metazoa | False | 18183 | 15591015 |
| Castor canadensis | 51338 | metazoa | False | 20186 | 6585248 |
| Catagonus wagneri | 51154 | metazoa | False | 19330 | 10390079 |
| Catenaria anguillulae | 765915 | fungi | False | 6373 | 1892804 |
| Caulochytrium protostelioides | 1555241 | fungi | False | 4673 | 1457063 |

Continued on next page

**Table 12 – continued from previous page**

| Name | Taxon ID | Kingdom | Seed | Number of Nodes | Number of Edges |
|---|---|---|---|---|---|
| Cavenderia fasciculata | 1054147 | protists | False | 7269 | 1882647 |
| Cavia aperea | 37548 | metazoa | False | 14148 | 8792393 |
| Cavia porcellus | 10141 | metazoa | False | 18006 | 12187025 |
| Cebus capucinus | 9516 | metazoa | False | 19799 | 5803086 |
| Cephalotus follicularis | 3775 | plant | False | 25299 | 12610350 |
| Ceraceosorus bombacis | 401625 | fungi | False | 5620 | 1844492 |
| Ceraceosorus guamensis | 1522189 | fungi | False | 5835 | 1720022 |
| Ceratocystis fimbriata | 1035309 | fungi | False | 6288 | 1968352 |
| Cercocebus atys | 9531 | metazoa | False | 19719 | 4959756 |
| Cercospora berteroae | 357750 | fungi | False | 9810 | 4129640 |
| Cercospora zeina | 348901 | fungi | False | 8511 | 3697298 |
| Chaetomium globosum | 306901 | fungi | False | 9087 | 1262137 |
| Chaetomium thermophilum | 759272 | fungi | False | 6581 | 954562 |
| Chara braunii | 69332 | plant | False | 18540 | 8743973 |
| Charadrius vociferus | 50402 | metazoa | False | 13006 | 6560406 |
| Chelonia mydas | 8469 | metazoa | False | 16284 | 2914544 |
| Chelonoidis abingdonii | 106734 | metazoa | False | 19739 | 16896203 |
| Chilo suppressalis | 168631 | metazoa | False | 12270 | 6196505 |
| Chiloscyllium punctatum | 137246 | metazoa | False | 23844 | 23775701 |
| Chinchilla lanigera | 34839 | metazoa | False | 17743 | 12779363 |
| Chlamydomonas eustigma | 1157962 | plant | False | 9899 | 3235618 |
| Chlorella sorokiniana | 3076 | plant | False | 7307 | 1697283 |
| Chlorella variabilis | 554065 | plant | False | 8287 | 1639354 |
| Chlorocebus sabaeus | 60711 | metazoa | False | 18852 | 5906810 |
| Chloropicon primus | 1764295 | plant | False | 6561 | 1492560 |
| Choanephora cucurbitarum | 101091 | fungi | False | 9218 | 3721744 |
| Choiromyces venosus | 1336337 | fungi | False | 9819 | 5289346 |
| Choloepus hoffmanni | 9358 | metazoa | False | 12148 | 7164402 |
| Chondrus crispus | 2769 | protists | False | 5002 | 845200 |
| Chrysemys picta | 8478 | metazoa | False | 20714 | 5273532 |
| Chrysolophus pictus | 9089 | metazoa | False | 11780 | 4098838 |
| Chytriomyces confervae | 246404 | fungi | False | 8924 | 4299939 |
| Cicer arietinum | 3827 | plant | False | 23474 | 5629043 |
| Cinnamomum micranthum | 337451 | plant | False | 23803 | 13611406 |
| Ciona intestinalis | 7719 | metazoa | False | 11270 | 1598954 |
| Ciona savignyi | 51511 | metazoa | False | 9033 | 4043992 |
| Citrus clementina | 85681 | plant | False | 22932 | 5470180 |
| Citrus unshiu | 55188 | plant | False | 25290 | 15003996 |
| Cladophialophora carrionii | 86049 | fungi | False | 9479 | 4405749 |
| Cladophialophora immunda | 569365 | fungi | False | 10943 | 5359274 |
| Cladophialophora psammophila | 1182543 | fungi | False | 11990 | 6754425 |
| Cladophialophora yegresii | 1182544 | fungi | False | 9136 | 4853571 |
| Claviceps purpurea | 1111077 | fungi | False | 7664 | 3322143 |
| Clavispora lusitaniae | 306902 | fungi | False | 5080 | 729323 |
| Clohesyomyces aquaticus | 1231657 | fungi | False | 11783 | 7095842 |
| Clonorchis sinensis | 79923 | metazoa | False | 8838 | 3206590 |
| Clunio marinus | 568069 | metazoa | False | 10285 | 5444772 |
| Clupea harengus | 7950 | metazoa | False | 23475 | 23104274 |
| Coccidioides immitis H5384 | 396776 | fungi | False | 7725 | 2910174 |

Continued on next page

**Table 12 – continued from previous page**

| Name | Taxon ID | Kingdom | Seed | Number of Nodes | Number of Edges |
|---|---|---|---|---|---|
| Coccidioides immitis RMSCC2394 | 404692 | fungi | False | 7447 | 2362427 |
| Coccidioides immitis RMSCC3703 | 454286 | fungi | False | 7725 | 3165728 |
| Coccidioides immitis RS | 246410 | fungi | False | 7350 | 1061650 |
| Coccidioides posadasii | 443226 | fungi | False | 7459 | 2397758 |
| Coccomyxa subellipsoidea | 574566 | plant | False | 7356 | 1106597 |
| Coemansia reversa | 763665 | fungi | False | 5393 | 1643505 |
| Coffea canephora | 49390 | plant | False | 23278 | 16455066 |
| Coleophoma cylindrospora | 1849047 | fungi | False | 12604 | 6200335 |
| Colinus virginianus | 9014 | metazoa | False | 16930 | 11963067 |
| Colletotrichum chlorophyti | 708187 | fungi | False | 9814 | 3995565 |
| Colletotrichum fioriniae | 1445577 | fungi | False | 12027 | 1320360 |
| Colletotrichum fructicola | 1213859 | fungi | False | 13632 | 7704832 |
| Colletotrichum gloeosporioides | 1237896 | fungi | False | 13175 | 6398702 |
| Colletotrichum graminicola | 645133 | fungi | False | 10561 | 4991797 |
| Colletotrichum higginsianum | 759273 | fungi | False | 13523 | 8584751 |
| Colletotrichum incanum | 1573173 | fungi | False | 11270 | 5102108 |
| Colletotrichum orbiculare | 1213857 | fungi | False | 11078 | 4980222 |
| Colletotrichum orchidophilum | 1209926 | fungi | False | 11366 | 5742816 |
| Colletotrichum salicis | 1209931 | fungi | False | 12000 | 6214468 |
| Colletotrichum sublineola | 1173701 | fungi | False | 10971 | 5487169 |
| Colletotrichum tanaceti | 1306861 | fungi | False | 10092 | 5507758 |
| Colletotrichum tofieldiae | 708197 | fungi | False | 11386 | 4873990 |
| Colletotrichum trifolii | 5466 | fungi | False | 10591 | 4656713 |
| Collichthys lucidus | 240159 | metazoa | False | 25883 | 32292374 |
| Colobus angolensis | 336983 | metazoa | False | 19585 | 5090634 |
| Columba livia | 8932 | metazoa | False | 14393 | 2960175 |
| Conidiobolus coronatus | 796925 | fungi | False | 6397 | 2491353 |
| Coniella lustricola | 2025994 | fungi | False | 8582 | 3709494 |
| Coniochaeta ligniaria | 1408157 | fungi | False | 10481 | 4966850 |
| Coniochaeta pulveracea | 177199 | fungi | False | 8365 | 3489322 |
| Coniosporium apollinis | 1168221 | fungi | False | 8142 | 3532579 |
| Coprinellus micaceus | 71717 | fungi | False | 12826 | 11020770 |
| Coprinopsis cinerea | 240176 | fungi | False | 9797 | 1372246 |
| Coprinopsis marcescibilis | 230819 | fungi | False | 11084 | 6771460 |
| Corchorus capsularis | 210143 | plant | False | 20056 | 8059573 |
| Corchorus olitorius | 93759 | plant | False | 24044 | 16483365 |
| Cordyceps confragosa | 1081108 | fungi | False | 10005 | 4254663 |
| Cordyceps fumosorosea | 1081104 | fungi | False | 9110 | 3638350 |
| Cordyceps javanica | 43265 | fungi | False | 9078 | 4082979 |
| Cordyceps sp. RAO2017 | 2004951 | fungi | False | 8434 | 3440076 |
| Corvus brachyrhynchos | 85066 | metazoa | False | 13015 | 6594561 |
| Corynespora cassiicola | 1448308 | fungi | False | 12528 | 6831569 |
| Cottoperca gobio | 56716 | metazoa | False | 21292 | 20880693 |
| Coturnix japonica | 93934 | metazoa | False | 15456 | 3432566 |
| Cricetulus griseus | 10029 | metazoa | False | 19383 | 7638343 |
| Crocodylus porosus | 8502 | metazoa | False | 14886 | 8718124 |
| Crucibulum laeve | 68775 | fungi | False | 10321 | 4977190 |
| Cryomyces minteri | 331657 | fungi | False | 11839 | 6090310 |
| Cryptococcus amylolentus | 1295533 | fungi | False | 6490 | 2411171 |

Continued on next page

**Table 12 – continued from previous page**

| Name | Taxon ID | Kingdom | Seed | Number of Nodes | Number of Edges |
|---|---|---|---|---|---|
| Cryptococcus depauperatus | 1295531 | fungi | False | 5472 | 1648237 |
| Cryptococcus gattii | 294750 | fungi | False | 5761 | 1912385 |
| Cryptosporidium muris | 441375 | protists | False | 2527 | 593306 |
| Cryptosporidium parvum | 353152 | protists | False | 2544 | 521535 |
| Cryptotermes secundus | 105785 | metazoa | False | 15120 | 9172958 |
| Cuculus canorus | 55661 | metazoa | False | 12888 | 6363395 |
| Cucumis melo | 1194695 | plant | False | 25342 | 4139628 |
| Cucumis sativus | 3659 | plant | False | 19707 | 4185676 |
| Culex quinquefasciatus | 7176 | metazoa | False | 16578 | 3363026 |
| Cutaneotrichosporon oleaginosum | 879819 | fungi | False | 6366 | 2038343 |
| Cyanidioschyzon merolae | 280699 | protists | False | 3875 | 471974 |
| Cyanistes caeruleus | 156563 | metazoa | False | 15170 | 3596370 |
| Cyberlindnera fabianii | 36022 | fungi | False | 4972 | 1376475 |
| Cyberlindnera jadinii | 983966 | fungi | False | 5167 | 1580737 |
| Cylindrobasidium torrendii | 1314674 | fungi | False | 10201 | 4590572 |
| Cynara cardunculus | 59895 | plant | False | 24524 | 8370239 |
| Cynoglossus semilaevis | 244447 | metazoa | False | 21085 | 7337731 |
| Cyphellophora europaea | 1220924 | fungi | False | 9802 | 3589259 |
| Cyphomyrmex costatus | 456900 | metazoa | False | 11756 | 5227679 |
| Cyprinodon variegatus | 28743 | metazoa | False | 22717 | 7018133 |
| Cytospora leucostoma | 1230097 | fungi | False | 8944 | 3808697 |
| Dacryopinax primogenitus | 1858805 | fungi | False | 7780 | 3503799 |
| Dactylellina haptotyla | 1284197 | fungi | False | 8295 | 3821225 |
| Daedalea quercina | 1314783 | fungi | False | 9439 | 4684901 |
| Daldinia sp. EC12 | 1001832 | fungi | False | 9678 | 5523182 |
| Danaus plexippus | 278856 | metazoa | False | 11902 | 1983541 |
| Danionella translucida | 623744 | metazoa | False | 22028 | 12110509 |
| Daphnia magna | 35525 | metazoa | False | 13640 | 6557433 |
| Daphnia pulex | 6669 | metazoa | False | 16864 | 3170036 |
| Dasypus novemcinctus | 9361 | metazoa | False | 22309 | 22877421 |
| Daucus carota | 79200 | plant | False | 26357 | 7826668 |
| Debaryomyces hansenii | 284592 | fungi | False | 5544 | 826184 |
| Delphinapterus leucas | 9749 | metazoa | False | 16928 | 5721559 |
| Dendrobium catenatum | 906689 | plant | False | 21362 | 4282425 |
| Dendroctonus ponderosae | 77166 | metazoa | False | 10908 | 1713753 |
| Denticeps clupeoides | 299321 | metazoa | False | 23666 | 23940506 |
| Dentipellis fragilis | 205917 | fungi | False | 8239 | 4127810 |
| Diaphorina citri | 121845 | metazoa | False | 16020 | 10531960 |
| Diaporthe ampelina | 1214573 | fungi | False | 9751 | 4216617 |
| Diaporthe helianthi | 158607 | fungi | False | 11023 | 5078422 |
| Dichanthelium oligosanthes | 888268 | plant | False | 22674 | 18439357 |
| Dichomitus squalens | 114155 | fungi | False | 10534 | 1615442 |
| Dictyocaulus viviparus | 29172 | metazoa | False | 11371 | 5450530 |
| Dictyostelium purpureum | 5786 | protists | False | 8074 | 1717790 |
| Dimargaris cristalligena | 215637 | fungi | False | 5358 | 1679732 |
| Dinothrombium tinctorium | 1965070 | metazoa | False | 12492 | 4764652 |
| Diplocarpon rosae | 946125 | fungi | False | 12885 | 9580989 |
| Diplodia corticola | 236234 | fungi | False | 9556 | 4965895 |
| Diplodia seriata | 420778 | fungi | False | 7889 | 3263960 |

Continued on next page

**Table 12 – continued from previous page**

| Name | Taxon ID | Kingdom | Seed | Number of Nodes | Number of Edges |
|---|---|---|---|---|---|
| Diploscapter pachys | 2018661 | metazoa | False | 19406 | 16615468 |
| Dipodomys ordii | 10020 | metazoa | False | 16814 | 12063593 |
| Diversispora epigaea | 1348612 | fungi | False | 14213 | 11310774 |
| Dothistroma septosporum | 675120 | fungi | False | 9164 | 4281150 |
| Dracunculus medinensis | 318479 | metazoa | False | 8861 | 3819177 |
| Drechmeria coniospora | 98403 | fungi | False | 7060 | 2496570 |
| Dromaius novaehollandiae | 8790 | metazoa | False | 15275 | 9336572 |
| Drosophila ananassae | 7217 | metazoa | False | 13157 | 2980809 |
| Drosophila busckii | 30019 | metazoa | False | 11398 | 4138644 |
| Drosophila grimshawi | 7222 | metazoa | False | 13273 | 2419622 |
| Drosophila guanche | 7266 | metazoa | False | 12430 | 2129385 |
| Drosophila navojoa | 7232 | metazoa | False | 12755 | 2086730 |
| Drosophila persimilis | 7234 | metazoa | False | 14456 | 2578694 |
| Drosophila pseudoobscura | 46245 | metazoa | False | 13113 | 2277476 |
| Drosophila sechellia | 7238 | metazoa | False | 14426 | 2696211 |
| Drosophila simulans | 7240 | metazoa | False | 13663 | 2183329 |
| Drosophila virilis | 7244 | metazoa | False | 12534 | 2667850 |
| Drosophila willistoni | 7260 | metazoa | False | 12878 | 2237251 |
| Dufourea novaeangliae | 178035 | metazoa | False | 8994 | 3944529 |
| Echeneis naucrates | 173247 | metazoa | False | 21361 | 20616206 |
| Echinococcus granulosus | 6210 | metazoa | False | 7477 | 2762353 |
| Echinococcus multilocularis | 6211 | metazoa | False | 7932 | 3758789 |
| Echinops telfairi | 9371 | metazoa | False | 16084 | 13244127 |
| Ectocarpus siliculosus | 2880 | protists | False | 9732 | 6129642 |
| Edhazardia aedis | 1003232 | fungi | False | 1405 | 173618 |
| Egretta garzetta | 188379 | metazoa | False | 11505 | 1888458 |
| Elaeophora elaphi | 1147741 | metazoa | False | 9010 | 3960566 |
| Electrophorus electricus | 8005 | metazoa | False | 22408 | 8271287 |
| Elsinoe australis | 40998 | fungi | False | 8116 | 3248446 |
| Elysia chlorotica | 188477 | metazoa | False | 17024 | 6818668 |
| Emergomyces pasteurianus | 1447872 | fungi | False | 7931 | 3831063 |
| Emiliania huxleyi | 2903 | protists | False | 17796 | 5085914 |
| Emmonsia crescens | 73230 | fungi | False | 7848 | 3522271 |
| Emmonsia sp. CAC2015a | 1658172 | fungi | False | 7787 | 3054970 |
| Encephalitozoon cuniculi | 284813 | fungi | False | 1353 | 172630 |
| Enhydra lutris | 391180 | metazoa | False | 19046 | 5919169 |
| Entamoeba histolytica | 5759 | protists | False | 4665 | 1748466 |
| Enterobius vermicularis | 51028 | metazoa | False | 9478 | 3919527 |
| Enterocytozoon bieneusi | 481877 | fungi | False | 1638 | 190953 |
| Enterocytozoon hepatopenaei | 646526 | fungi | False | 1106 | 129838 |
| Epicoccum nigrum | 105696 | fungi | False | 9979 | 4331717 |
| Eptatretus burgeri | 7764 | metazoa | False | 15609 | 11402285 |
| Equus asinus | 83772 | metazoa | False | 19622 | 6524495 |
| Equus caballus | 9796 | metazoa | False | 20559 | 7197116 |
| Eremothecium cymbalariae | 931890 | fungi | False | 4202 | 614432 |
| Eremothecium gossypii | 284811 | fungi | False | 4531 | 703398 |
| Eremothecium sinecaudum | 45286 | fungi | False | 4325 | 717597 |
| Erinaceus europaeus | 9365 | metazoa | False | 14308 | 8052911 |
| Erpetoichthys calabaricus | 27687 | metazoa | False | 21804 | 18915329 |

Continued on next page

Table 12 – continued from previous page

| Name | Taxon ID | Kingdom | Seed | Number of Nodes | Number of Edges |
|---|---|---|---|---|---|
| Erysiphe necator | 52586 | fungi | False | 5394 | 1745314 |
| Erysiphe pulchra | 225359 | fungi | False | 5880 | 1837500 |
| Erythranthe guttata | 4155 | plant | False | 25630 | 16300254 |
| Erythrura gouldiae | 44316 | metazoa | False | 16069 | 3719546 |
| Escovopsis weberi | 150374 | fungi | False | 6489 | 2173079 |
| Esox lucius | 8010 | metazoa | False | 23513 | 8332048 |
| Eucalyptus grandis | 71139 | plant | False | 31136 | 8114973 |
| Eumeta japonica | 151549 | metazoa | False | 27740 | 23969992 |
| Eutrema salsugineum | 72664 | plant | False | 24729 | 7242380 |
| Eutypa lata | 1287681 | fungi | False | 10406 | 1287382 |
| Exidia glandulosa | 1314781 | fungi | False | 14591 | 10724406 |
| Exophiala aquamarina | 1182545 | fungi | False | 12135 | 6757524 |
| Exophiala dermatitidis | 858893 | fungi | False | 8212 | 3275155 |
| Exophiala mesophila | 212818 | fungi | False | 8333 | 3053606 |
| Exophiala oligosperma | 215243 | fungi | False | 10496 | 4509253 |
| Exophiala sideris | 1016849 | fungi | False | 9124 | 4362834 |
| Exophiala spinifera | 91928 | fungi | False | 10773 | 4809820 |
| Exophiala xenobiotica | 348802 | fungi | False | 10605 | 4825063 |
| Exserohilum turcica | 671987 | fungi | False | 9627 | 3974292 |
| Fasciola gigantica | 46835 | metazoa | False | 9375 | 4454045 |
| Felis catus | 9685 | metazoa | False | 19224 | 6465547 |
| Fibroporia radiculosa | 599839 | fungi | False | 7847 | 2885387 |
| Fibularhizoctonia sp. CBS109695 | 436010 | fungi | False | 17847 | 19012914 |
| Ficedula albicollis | 59894 | metazoa | False | 14798 | 2986392 |
| Folsomia candida | 158441 | metazoa | False | 15639 | 1883767 |
| Fomitopsis pinicola | 743788 | fungi | False | 11029 | 5241397 |
| Fomitopsis rosea | 34475 | fungi | False | 9400 | 5092199 |
| Fonsecaea erecta | 1367422 | fungi | False | 10565 | 4792572 |
| Fonsecaea multimorphosa | 1442371 | fungi | False | 11038 | 5653369 |
| Fonsecaea pedrosoi | 1442368 | fungi | False | 11071 | 5554589 |
| Fonticula alba | 691883 | other | False | 4055 | 868089 |
| Friedmanniomyces endolithicus | 329885 | fungi | False | 15427 | 12415007 |
| Friedmanniomyces simplex | 329884 | fungi | False | 11518 | 5839647 |
| Fukomys damarensis | 885580 | metazoa | False | 17617 | 12057399 |
| Fundulus heteroclitus | 8078 | metazoa | False | 22576 | 16087148 |
| Furculomyces boomerangus | 61424 | fungi | False | 5222 | 1326996 |
| Fusarium fasciculatum | 2594813 | fungi | False | 11640 | 6103956 |
| Fusarium fujikuroi | 1279085 | fungi | False | 12941 | 6849034 |
| Fusarium graminearum | 229533 | fungi | False | 11326 | 1522997 |
| Fusarium kuroshium | 2010991 | fungi | False | 14103 | 9454054 |
| Fusarium longipes | 694270 | fungi | False | 10507 | 4388841 |
| Fusarium nygamai | 42673 | fungi | False | 14066 | 6987567 |
| Fusarium oxysporum Fo5176 | 660025 | fungi | False | 15085 | 8705017 |
| Fusarium oxysporum f. sp. cubense race 1 | 1229664 | fungi | False | 13454 | 6885833 |
| Fusarium oxysporum f. sp. cubense race 4 | 1229665 | fungi | False | 12475 | 6678967 |
| Fusarium oxysporum f. sp. lycopersici | 426428 | fungi | False | 14487 | 2913780 |
| Fusarium oxysporum f. sp. radiciscucumerinum | 327505 | fungi | False | 14738 | 9122261 |
| Fusarium poae | 36050 | fungi | False | 12433 | 6642289 |
| Fusarium sp. AF4 | 1325735 | fungi | False | 15359 | 10200736 |

Continued on next page

**Table 12 – continued from previous page**

| Name | Taxon ID | Kingdom | Seed | Number of Nodes | Number of Edges |
|---|---|---|---|---|---|
| Fusarium sp. AF6 | 1325737 | fungi | False | 13359 | 7194512 |
| Fusarium sp. AF8 | 1325734 | fungi | False | 13899 | 7474134 |
| Fusarium sporotrichioides | 5514 | fungi | False | 10973 | 4548018 |
| Fusarium venenatum | 56646 | fungi | False | 11767 | 5847774 |
| Fusarium verticillioides | 334819 | fungi | False | 13139 | 2006988 |
| Gadus morhua | 8049 | metazoa | False | 19630 | 17590791 |
| Gaeumannomyces tritici | 644352 | fungi | False | 10036 | 5665861 |
| Galerina marginata | 685588 | fungi | False | 14190 | 9941041 |
| Gambusia affinis | 33528 | metazoa | False | 21421 | 17969231 |
| Ganoderma sinense | 1077348 | fungi | False | 11521 | 6969388 |
| Gasterosteus aculeatus | 69293 | metazoa | False | 20410 | 21758335 |
| Gelatoporia subvermispora | 914234 | fungi | False | 9241 | 4331562 |
| Geotrichum candidum | 1173061 | fungi | False | 5874 | 2090322 |
| Giardia lamblia ATCC 50803 | 184922 | protists | False | 2667 | 487534 |
| Giardia lamblia P15 | 658858 | protists | False | 2455 | 396810 |
| Gigaspora rosea | 44941 | fungi | False | 15861 | 7674420 |
| Glarea lozoyensis | 1116229 | fungi | False | 10435 | 1117504 |
| Globisporangium ultimum | 431595 | protists | False | 10726 | 4690332 |
| Glomus cerebriforme | 658196 | fungi | False | 12288 | 4912842 |
| Glossina austeni | 7395 | metazoa | False | 13432 | 6576011 |
| Glossina brevipalpis | 37001 | metazoa | False | 11423 | 5204407 |
| Glossina fuscipes | 201502 | metazoa | False | 13429 | 6364586 |
| Glossina morsitans | 37546 | metazoa | False | 11100 | 5134007 |
| Glossina pallidipes | 7398 | metazoa | False | 12695 | 6495739 |
| Glossina palpalis | 67801 | metazoa | False | 13210 | 6527575 |
| Golovinomyces cichoracearum | 62708 | fungi | False | 5496 | 2171381 |
| Golovinomyces magnicellulatus | 62714 | fungi | False | 7645 | 3609008 |
| Gonapodya prolifera | 1344416 | fungi | False | 8408 | 3533287 |
| Gonium pectorale | 33097 | plant | False | 11199 | 4026318 |
| Gopherus agassizii | 38772 | metazoa | False | 19734 | 15348086 |
| Gorilla gorilla | 9593 | metazoa | False | 20527 | 6974658 |
| Gossypium hirsutum | 3635 | plant | False | 57908 | 37801058 |
| Gossypium raimondii | 29730 | plant | False | 34370 | 14838623 |
| Gouania willdenowi | 441366 | metazoa | False | 22779 | 25246833 |
| Gracilariopsis chorda | 448386 | protists | False | 5861 | 1450190 |
| Grifola frondosa | 5627 | fungi | False | 9809 | 4872503 |
| Grosmannia clavigera | 655863 | fungi | False | 7530 | 3297257 |
| Guillardia theta | 905079 | protists | False | 12016 | 5310141 |
| Gymnopilus dilepis | 231916 | fungi | False | 10442 | 6240975 |
| Habropoda laboriosa | 597456 | metazoa | False | 10410 | 5378260 |
| Haemonchus placei | 6290 | metazoa | False | 14154 | 7908967 |
| Hamiltosporidium magnivora | 148818 | fungi | False | 1769 | 262377 |
| Hamiltosporidium tvaerminnensis | 1176355 | fungi | False | 2373 | 399627 |
| Handroanthus impetiginosus | 429701 | plant | False | 26619 | 22324842 |
| Hanseniaspora osmophila | 56408 | fungi | False | 4182 | 789579 |
| Hanseniaspora uvarum | 29833 | fungi | False | 3450 | 627602 |
| Haplochromis burtoni | 8153 | metazoa | False | 22825 | 22535667 |
| Harpegnathos saltator | 610380 | metazoa | False | 12028 | 1594128 |
| Hebeloma cylindrosporum | 686832 | fungi | False | 10418 | 6350193 |

Continued on next page

**Table 12 – continued from previous page**

| Name | Taxon ID | Kingdom | Seed | Number of Nodes | Number of Edges |
|---|---|---|---|---|---|
| Helianthus annuus | 4232 | plant | False | 39824 | 15040997 |
| Helicocarpus griseus | 1447875 | fungi | False | 9079 | 4353487 |
| Helicosporidium sp. ATCC 50920 | 1291522 | plant | False | 4693 | 933797 |
| Heliocybe sulcata | 5364 | fungi | False | 9359 | 4669257 |
| Heliothis virescens | 7102 | metazoa | False | 12076 | 7134973 |
| Helobdella robusta | 6412 | metazoa | False | 14580 | 4031860 |
| Hericium alpestre | 135208 | fungi | False | 8729 | 3402213 |
| Hesseltinella vesiculosa | 101127 | fungi | False | 8492 | 3190381 |
| Heterobasidion irregulare | 747525 | fungi | False | 9399 | 1379105 |
| Heterocephalus glaber | 10181 | metazoa | False | 21001 | 7490506 |
| Heterostelium album | 670386 | protists | False | 7456 | 2121662 |
| Hippocampus comes | 109280 | metazoa | False | 20441 | 6490135 |
| Hirundo rustica | 333673 | metazoa | False | 26762 | 25999586 |
| Histoplasma capsulatum G186AR | 447093 | fungi | False | 7196 | 2999611 |
| Histoplasma capsulatum H143 | 544712 | fungi | False | 7449 | 3164754 |
| Histoplasma capsulatum H88 | 544711 | fungi | False | 7289 | 3156354 |
| Histoplasma capsulatum NAm1 | 339724 | fungi | False | 7726 | 1142224 |
| Hordeum vulgare | 112509 | plant | False | 31232 | 37673942 |
| Hortaea werneckii | 1157616 | fungi | False | 14496 | 9057012 |
| Hucho hucho | 62062 | metazoa | False | 49553 | 144917507 |
| Hyaloperonospora arabidopsidis | 559515 | protists | False | 7456 | 2885979 |
| Hyaloscypha bicolor | 1095630 | fungi | False | 14117 | 8928822 |
| Hyaloscypha variabilis | 1149755 | fungi | False | 15737 | 10236753 |
| Hydatigera taeniaeformis | 6205 | metazoa | False | 9468 | 5538733 |
| Hymenolepis diminuta | 6216 | metazoa | False | 8640 | 3940340 |
| Hypholoma sublateritium | 945553 | fungi | False | 12097 | 7961849 |
| Hyphopichia burtonii | 984485 | fungi | False | 5362 | 1479279 |
| Hypoxylon sp. CI4A | 1001833 | fungi | False | 9957 | 5459364 |
| Hypoxylon sp. CO275 | 1001938 | fungi | False | 10434 | 6316215 |
| Hypsizygus marmoreus | 39966 | fungi | False | 10411 | 5171977 |
| Ichthyophthirius multifiliis | 857967 | protists | False | 5990 | 2789467 |
| Ictalurus punctatus | 7998 | metazoa | False | 23170 | 8578513 |
| Ictidomys tridecemlineatus | 43179 | metazoa | False | 18380 | 12544556 |
| Ixodes scapularis | 6945 | metazoa | False | 13108 | 2431906 |
| Jaapia argillacea | 933084 | fungi | False | 10951 | 7640681 |
| Jaculus jaculus | 51337 | metazoa | False | 17744 | 14111801 |
| Jaminaea rosea | 1569628 | fungi | False | 5357 | 1719202 |
| Jatropha curcas | 180498 | plant | False | 20872 | 4393339 |
| Juglans regia | 51240 | plant | False | 34471 | 12317525 |
| Junco hyemalis | 40217 | metazoa | False | 14203 | 8886539 |
| Kalmanozyma brasiliensis | 1365824 | fungi | False | 5365 | 1636039 |
| Kazachstania africana | 1071382 | fungi | False | 5075 | 950015 |
| Kazachstania naganishii | 1071383 | fungi | False | 5021 | 931656 |
| Kazachstania saulgeensis | 1789683 | fungi | False | 5071 | 991896 |
| Klebsormidium nitens | 105231 | plant | False | 10881 | 4633170 |
| Kluyveromyces lactis | 284590 | fungi | False | 4751 | 730337 |
| Kockovaella imperatae | 4999 | fungi | False | 6112 | 1886609 |
| Kryptolebias marmoratus | 37003 | metazoa | False | 21600 | 7241675 |
| Kuraishia capsulata | 1382522 | fungi | False | 5254 | 1530699 |

Continued on next page

**Table 12 – continued from previous page**

| Name | Taxon ID | Kingdom | Seed | Number of Nodes | Number of Edges |
|---|---|---|---|---|---|
| Kwoniella bestiolae | 1296100 | fungi | False | 6941 | 2366999 |
| Kwoniella dejecticola | 1296121 | fungi | False | 6838 | 2033243 |
| Kwoniella heveanensis | 1296120 | fungi | False | 6552 | 2083298 |
| Kwoniella mangroviensis | 1331196 | fungi | False | 6704 | 2308897 |
| Kwoniella pini | 1296096 | fungi | False | 6461 | 2154338 |
| Labeo rohita | 84645 | metazoa | False | 28276 | 20766213 |
| Labrus bergylta | 56723 | metazoa | False | 26618 | 29461365 |
| Laccaria amethystina | 1095629 | fungi | False | 12002 | 8140171 |
| Laccaria bicolor | 486041 | fungi | False | 10383 | 2186777 |
| Lachancea dasiensis | 1266660 | fungi | False | 4853 | 814605 |
| Lachancea fermentati | 4955 | fungi | False | 4971 | 852920 |
| Lachancea lanzarotensis | 1245769 | fungi | False | 4831 | 827315 |
| Lachancea mirantina | 1230905 | fungi | False | 4820 | 787042 |
| Lachancea thermotolerans | 559295 | fungi | False | 4845 | 736808 |
| Lactuca sativa | 4236 | plant | False | 31168 | 11645870 |
| Laetiporus sulphureus | 1314785 | fungi | False | 9995 | 4865521 |
| Laodelphax striatellus | 195883 | metazoa | False | 12829 | 6031709 |
| Larimichthys crocea | 215358 | metazoa | False | 22419 | 7259761 |
| Lasius niger | 67767 | metazoa | False | 13995 | 6184214 |
| Lates calcarifer | 8187 | metazoa | False | 24550 | 7327195 |
| Latimeria chalumnae | 7897 | metazoa | False | 19304 | 4911511 |
| Leersia perrieri | 77586 | plant | False | 23845 | 22604208 |
| Leishmania braziliensis | 5660 | protists | False | 5263 | 956474 |
| Leishmania infantum | 5671 | protists | False | 5250 | 1013730 |
| Leishmania major | 5664 | protists | False | 5275 | 838868 |
| Lentinula edodes | 5353 | fungi | False | 8871 | 3329484 |
| Lentinus tigrinus | 1328759 | fungi | False | 11402 | 7324437 |
| Lepidothrix coronata | 321398 | metazoa | False | 15015 | 9770776 |
| Lepisosteus oculatus | 7918 | metazoa | False | 18151 | 11048423 |
| Leptonychotes weddellii | 9713 | metazoa | False | 13136 | 2558557 |
| Leptosphaeria maculans | 985895 | fungi | False | 8548 | 3080995 |
| Leptotrombidium deliense | 299467 | metazoa | False | 12976 | 5644330 |
| Leucoagaricus sp. SymCcos | 1714833 | fungi | False | 10624 | 4693669 |
| Leucosporidium creatinivorum | 106004 | fungi | False | 7338 | 3049024 |
| Lichtheimia corymbifera | 1263082 | fungi | False | 8880 | 3846378 |
| Linderina pennispora | 61395 | fungi | False | 6205 | 2172068 |
| Lingula anatina | 7574 | metazoa | False | 22423 | 4137247 |
| Lipomyces starkeyi | 675824 | fungi | False | 6266 | 1982012 |
| Lipotes vexillifer | 118797 | metazoa | False | 18705 | 5832383 |
| Litomosoides sigmodontis | 42156 | metazoa | False | 8254 | 3485197 |
| Loa loa | 7209 | metazoa | False | 9442 | 2478897 |
| Lobosporangium transversale | 64571 | fungi | False | 8033 | 3178801 |
| Lodderomyces elongisporus | 379508 | fungi | False | 5174 | 764747 |
| Lomentospora prolificans | 41688 | fungi | False | 8157 | 2969682 |
| Lonchura striata | 299123 | metazoa | False | 15584 | 3405792 |
| Lottia gigantea | 225164 | metazoa | False | 16627 | 2422383 |
| Loxodonta africana | 9785 | metazoa | False | 19897 | 5569954 |
| Lucilia cuprina | 7375 | metazoa | False | 10528 | 1780068 |
| Lupinus angustifolius | 3871 | plant | False | 28824 | 11423838 |

Continued on next page

**Table 12 – continued from previous page**

| Name | Taxon ID | Kingdom | Seed | Number of Nodes | Number of Edges |
|---|---|---|---|---|---|
| Lynx canadensis | 61383 | metazoa | False | 18844 | 5135983 |
| Macaca fascicularis | 9541 | metazoa | False | 20237 | 7735022 |
| Macaca mulatta | 9544 | metazoa | False | 20591 | 6629959 |
| Macaca nemestrina | 9545 | metazoa | False | 19788 | 5038486 |
| Macleaya cordata | 56857 | plant | False | 19786 | 9464919 |
| Macrophomina phaseolina | 1126212 | fungi | False | 10959 | 5435420 |
| Macrostomum lignano | 282301 | metazoa | False | 33086 | 56285929 |
| Madurella mycetomatis | 100816 | fungi | False | 8851 | 3445824 |
| Magnaporthiopsis poae | 644358 | fungi | False | 8373 | 4142906 |
| Malassezia globosa | 425265 | fungi | False | 4054 | 599843 |
| Malassezia pachydermatis | 77020 | fungi | False | 3951 | 1004340 |
| Malassezia sympodialis | 1230383 | fungi | False | 4199 | 577209 |
| Malassezia vespertilionis | 2020962 | fungi | False | 3580 | 849197 |
| Malus baccata | 106549 | plant | False | 38147 | 30161588 |
| Malus domestica | 3750 | plant | False | 33062 | 9858472 |
| Manacus vitellinus | 328815 | metazoa | False | 15543 | 9863217 |
| Mandrillus leucophaeus | 9568 | metazoa | False | 19489 | 4754015 |
| Manihot esculenta | 3983 | plant | False | 28368 | 9258169 |
| Marmota marmota | 9994 | metazoa | False | 20010 | 16684064 |
| Marssonina brunnea | 1072389 | fungi | False | 8068 | 923273 |
| Marssonina coronariae | 503106 | fungi | False | 7506 | 2846387 |
| Mastacembelus armatus | 205130 | metazoa | False | 22953 | 24467281 |
| Maylandia zebra | 106582 | metazoa | False | 26805 | 8878848 |
| Medicago truncatula | 3880 | plant | False | 34041 | 12188443 |
| Megaselia scalaris | 36166 | metazoa | False | 8158 | 3716757 |
| Meira miltonrushii | 1280837 | fungi | False | 5847 | 1817975 |
| Melampsora laricipopulina | 747676 | fungi | False | 7805 | 1148310 |
| Meleagris gallopavo | 9103 | metazoa | False | 1865 | 11190 |
| Melipona quadrifasciata | 166423 | metazoa | False | 9879 | 4335141 |
| Melopsittacus undulatus | 13146 | metazoa | False | 14292 | 8645152 |
| Meriones unguiculatus | 10047 | metazoa | False | 20743 | 6360262 |
| Mesocestoides corti | 53468 | metazoa | False | 7949 | 3253793 |
| Mesocricetus auratus | 10036 | metazoa | False | 18093 | 14376851 |
| Metarhizium acridum | 655827 | fungi | False | 9151 | 1274260 |
| Metarhizium album | 1081103 | fungi | False | 7763 | 3541619 |
| Metarhizium anisopliae | 1291518 | fungi | False | 10193 | 3826620 |
| Metarhizium rileyi | 1081105 | fungi | False | 8052 | 3509229 |
| Metschnikowia aff. | 2163413 | fungi | False | 5046 | 1315253 |
| Metschnikowia bicuspidata | 869754 | fungi | False | 4961 | 1415505 |
| Metschnikowia sp. JCM33374 | 2562755 | fungi | False | 5313 | 1564011 |
| Meyerozyma guilliermondii | 294746 | fungi | False | 5414 | 711868 |
| Meyerozyma sp. JA9 | 2028340 | fungi | False | 4729 | 1164623 |
| Micractinium conductrix | 554055 | plant | False | 7369 | 1692646 |
| Microbotryum intermedium | 269621 | fungi | False | 6687 | 2436551 |
| Microbotryum lychnidisdioicae | 683840 | fungi | False | 6249 | 2135021 |
| Microbotryum saponariae | 289078 | fungi | False | 7667 | 2947774 |
| Microbotryum silenesdioicae | 796604 | fungi | False | 8744 | 3275423 |
| Microcebus murinus | 30608 | metazoa | False | 18652 | 15297029 |
| Microdochium bolleyi | 196109 | fungi | False | 10529 | 6605866 |

Continued on next page

**Table 12 – continued from previous page**

| Name | Taxon ID | Kingdom | Seed | Number of Nodes | Number of Edges |
|---|---|---|---|---|---|
| Micromonas commoda | 296587 | plant | False | 7499 | 1551901 |
| Micromonas pusilla | 564608 | plant | False | 7209 | 1483770 |
| Microsporum canis | 554155 | fungi | False | 7616 | 3333461 |
| Microtus ochrogaster | 79684 | metazoa | False | 18968 | 17063256 |
| Millerozyma farinosa | 559304 | fungi | False | 8036 | 3698941 |
| Mixia osmundae | 764103 | fungi | False | 5238 | 1529583 |
| Moelleriella libera | 1081109 | fungi | False | 7729 | 2604899 |
| Moesziomyces antarcticus | 1151754 | fungi | False | 5689 | 1637412 |
| Mola mola | 94237 | metazoa | False | 21090 | 19962126 |
| Monascus purpureus | 5098 | fungi | False | 7552 | 2799035 |
| Moniliophthora perniciosa | 554373 | fungi | False | 10449 | 1441734 |
| Moniliophthora roreri | 1381753 | fungi | False | 13463 | 1701806 |
| Monodelphis domestica | 13616 | metazoa | False | 21185 | 6013336 |
| Monopterus albus | 43700 | metazoa | False | 21675 | 8245483 |
| Monoraphidium neglectum | 145388 | plant | False | 11743 | 2333861 |
| Monosiga brevicollis | 81824 | other | False | 6742 | 1536891 |
| Monosporascus ibericus | 155417 | fungi | False | 9770 | 5793076 |
| Monosporascus sp. 5C6A | 2211642 | fungi | False | 10749 | 5557080 |
| Monosporascus sp. CRB83 | 2211644 | fungi | False | 10157 | 4989214 |
| Monosporascus sp. CRB92 | 2211643 | fungi | False | 10263 | 5113627 |
| Monosporascus sp. GIB2 | 2211647 | fungi | False | 10137 | 5472169 |
| Monosporascus sp. MC138B | 2211646 | fungi | False | 9213 | 4554740 |
| Monosporascus sp. MG133 | 2211645 | fungi | False | 10006 | 4827929 |
| Monosporascus sp. mg162 | 1081914 | fungi | False | 10220 | 5788664 |
| Morchella conica | 1392247 | fungi | False | 7874 | 3870221 |
| Mortierella elongata | 1314771 | fungi | False | 9743 | 4758629 |
| Morus notabilis | 981085 | plant | False | 20847 | 11303329 |
| Moschus moschiferus | 68415 | metazoa | False | 21034 | 13976172 |
| Mucor ambiguus | 91626 | fungi | False | 9253 | 4396774 |
| Mucor circinelloides f. circinelloides | 1220926 | fungi | False | 9971 | 4473495 |
| Mucor circinelloides f. lusitanicus | 747725 | fungi | False | 9500 | 4321707 |
| Mucuna pruriens | 157652 | plant | False | 38860 | 9368106 |
| Mus caroli | 10089 | metazoa | False | 18709 | 5124776 |
| Mus pahari | 10093 | metazoa | False | 18571 | 5221462 |
| Mus spicilegus | 10103 | metazoa | False | 21924 | 14522801 |
| Mus spretus | 10096 | metazoa | False | 18957 | 6947272 |
| Musa acuminata | 214687 | plant | False | 30631 | 13742732 |
| Musa balbisiana | 52838 | plant | False | 26474 | 25949715 |
| Musca domestica | 7370 | metazoa | False | 12959 | 2143112 |
| Mustela putorius | 9669 | metazoa | False | 18769 | 4791894 |
| Myotis lucifugus | 59463 | metazoa | False | 19584 | 17664123 |
| Myripristis murdjan | 586833 | metazoa | False | 23454 | 25932695 |
| Nadsonia fulvescens | 857566 | fungi | False | 4951 | 1578219 |
| Naegleria gruberi | 5762 | protists | False | 8413 | 2964668 |
| Naematelia encephala | 71784 | fungi | False | 6523 | 1760034 |
| Nannizzia gypsea | 535722 | fungi | False | 7563 | 3285782 |
| Nannospalax galili | 1026970 | metazoa | False | 18499 | 5970634 |
| Nasonia vitripennis | 7425 | metazoa | False | 12746 | 1668961 |
| Naumovozyma castellii | 1064592 | fungi | False | 5233 | 995588 |

Continued on next page

**Table 12 – continued from previous page**

| Name | Taxon ID | Kingdom | Seed | Number of Nodes | Number of Edges |
|---|---|---|---|---|---|
| Naumovozyma dairenensis | 1071378 | fungi | False | 5209 | 1003339 |
| Necator americanus | 51031 | metazoa | False | 14809 | 2974823 |
| Nectria haematococca | 660122 | fungi | False | 14108 | 2965446 |
| Nematocida displodere | 1805483 | fungi | False | 1201 | 130053 |
| Nematocida parisii | 935791 | fungi | False | 1249 | 146845 |
| Nematocida sp. 1 ERTm2 | 944018 | fungi | False | 1271 | 161151 |
| Neocallimastix californiae | 1754190 | fungi | False | 14197 | 9922963 |
| Neofusicoccum parvum | 1287680 | fungi | False | 9388 | 1045935 |
| Neogobius melanostomus | 47308 | metazoa | False | 21949 | 21165111 |
| Neolamprologus brichardi | 32507 | metazoa | False | 23267 | 25034493 |
| Neolecta irregularis | 1198029 | fungi | False | 4398 | 974117 |
| Neolentinus lepideus | 1314782 | fungi | False | 10145 | 5690125 |
| Neomonachus schauinslandi | 29088 | metazoa | False | 18625 | 4822798 |
| Neonectria ditissima | 78410 | fungi | False | 11423 | 5886616 |
| Neophocaena asiaeorientalis | 1706337 | metazoa | False | 18052 | 6429275 |
| Neotoma lepida | 56216 | metazoa | False | 22932 | 33129653 |
| Neovison vison | 452646 | metazoa | False | 19323 | 5669432 |
| Neurospora tetrasperma | 510952 | fungi | False | 7910 | 1106746 |
| Nicotiana attenuata | 49451 | plant | False | 28295 | 8564459 |
| Nicotiana sylvestris | 4096 | plant | False | 30596 | 8616183 |
| Nicotiana tabacum | 4097 | plant | False | 57588 | 33551630 |
| Nipponia nippon | 128390 | metazoa | False | 13188 | 2254204 |
| Nippostrongylus brasiliensis | 27835 | metazoa | False | 16850 | 11120196 |
| Nomascus leucogenys | 61853 | metazoa | False | 19738 | 6284803 |
| Nosema bombycis | 578461 | fungi | False | 2104 | 379703 |
| Nosema ceranae | 578460 | fungi | False | 1204 | 121874 |
| Notamacropus eugenii | 9315 | metazoa | False | 15155 | 9289426 |
| Notechis scutatus | 8663 | metazoa | False | 18535 | 14718883 |
| Nothoprocta perdicaria | 30464 | metazoa | False | 15368 | 10354813 |
| Numida meleagris | 8996 | metazoa | False | 15413 | 3161836 |
| Ochotona princeps | 9978 | metazoa | False | 15728 | 9698535 |
| Octodon degus | 10160 | metazoa | False | 19893 | 24771371 |
| Octopus bimaculoides | 37653 | metazoa | False | 17528 | 2938528 |
| Odobenus rosmarus | 9708 | metazoa | False | 19177 | 6351837 |
| Ogataea parapolymorpha | 871575 | fungi | False | 4917 | 1460926 |
| Oidiodendron maius | 913774 | fungi | False | 13806 | 8949803 |
| Oidium neolycopersici | 212602 | fungi | False | 5669 | 2045137 |
| Onchocerca flexuosa | 387005 | metazoa | False | 13245 | 8039670 |
| Onchocerca ochengi | 42157 | metazoa | False | 11261 | 6887217 |
| Onchocerca volvulus | 6282 | metazoa | False | 8680 | 4137466 |
| Oncorhynchus mykiss | 8022 | metazoa | False | 45387 | 147667795 |
| Ooceraea biroi | 2015173 | metazoa | False | 12238 | 1581222 |
| Operophtera brumata | 104452 | metazoa | False | 13191 | 8350499 |
| Ophiocordyceps australis | 1399860 | fungi | False | 7355 | 3273662 |
| Ophiocordyceps camponotirufipedis | 2004952 | fungi | False | 6681 | 2322097 |
| Ophiocordyceps polyrhachisfurcata | 1330021 | fungi | False | 6279 | 2310073 |
| Ophiocordyceps sp. camponotileonardi | 2039875 | fungi | False | 6451 | 2359088 |
| Ophiocordyceps sp. camponotisaundersi | 2039874 | fungi | False | 6441 | 2189244 |
| Ophiocordyceps unilateralis | 268505 | fungi | False | 7215 | 2601283 |

Continued on next page

**Table 12 – continued from previous page**

| Name | Taxon ID | Kingdom | Seed | Number of Nodes | Number of Edges |
|---|---|---|---|---|---|
| Ophiostoma piceae | 1262450 | fungi | False | 8000 | 2939014 |
| Opisthocomus hoazin | 30419 | metazoa | False | 11421 | 4742520 |
| Opisthorchis felineus | 147828 | metazoa | False | 8867 | 3812605 |
| Opisthorchis viverrini | 6198 | metazoa | False | 9535 | 2503141 |
| Orchesella cincta | 48709 | metazoa | False | 14619 | 8990487 |
| Ordospora colligata | 1354746 | fungi | False | 1206 | 140794 |
| Oreochromis aureus | 47969 | metazoa | False | 24136 | 23069322 |
| Oreochromis niloticus | 8128 | metazoa | False | 27464 | 8183791 |
| Ornithorhynchus anatinus | 9258 | metazoa | False | 21159 | 6732285 |
| Oryctolagus cuniculus | 9986 | metazoa | False | 20232 | 7008235 |
| Oryza barthii | 65489 | plant | False | 25503 | 33187259 |
| Oryza brachyantha | 4533 | plant | False | 23886 | 7433442 |
| Oryza glaberrima | 4538 | plant | False | 24829 | 25576852 |
| Oryza glumipatula | 40148 | plant | False | 25962 | 29606444 |
| Oryza meridionalis | 40149 | plant | False | 21591 | 23595219 |
| Oryza punctata | 4537 | plant | False | 24559 | 29527078 |
| Oryza rufipogon | 4529 | plant | False | 26402 | 29470266 |
| Oryza sativa Indica | 39946 | plant | False | 30145 | 35557603 |
| Oryza sativa Japonica | 39947 | plant | False | 28354 | 33719175 |
| Oryza sativa f. spontanea | 4536 | plant | False | 26220 | 29917100 |
| Oryzias melastigma | 30732 | metazoa | False | 23134 | 21168197 |
| Ostreococcus lucimarinus | 436017 | plant | False | 5947 | 948622 |
| Otolemur garnettii | 30611 | metazoa | False | 19405 | 14464100 |
| Ovis aries | 9940 | metazoa | False | 20591 | 6861728 |
| Pachysolen tannophilus | 669874 | fungi | False | 5010 | 1329629 |
| Pan paniscus | 9597 | metazoa | False | 19988 | 6880259 |
| Pan troglodytes | 9598 | metazoa | False | 22067 | 8059125 |
| Panaeolus cyanescens | 181874 | fungi | False | 9627 | 4631727 |
| Panicum hallii | 1504633 | plant | False | 25609 | 20928226 |
| Panicum miliaceum | 4540 | plant | False | 48256 | 94129652 |
| Panthera pardus | 9691 | metazoa | False | 19418 | 6352175 |
| Panthera tigris | 74533 | metazoa | False | 17359 | 4859330 |
| Papaver somniferum | 3469 | plant | False | 32572 | 9940995 |
| Papilio machaon | 76193 | metazoa | False | 12714 | 1934838 |
| Papilio xuthus | 66420 | metazoa | False | 12279 | 6179466 |
| Papio anubis | 9555 | metazoa | False | 20196 | 5326430 |
| Paracoccidioides lutzii | 502779 | fungi | False | 7038 | 1055456 |
| Parambassis ranga | 210632 | metazoa | False | 23594 | 24768388 |
| Paramecium tetraurelia | 5888 | protists | False | 19837 | 17913536 |
| Paramicrosporidium saccamoebae | 1246581 | fungi | False | 2572 | 481133 |
| Paramormyrops kingsleyae | 1676925 | metazoa | False | 24245 | 9886181 |
| Paraphaeosphaeria sporulosa | 1460663 | fungi | False | 11370 | 4947449 |
| Parasitella parasitica | 35722 | fungi | False | 10206 | 4621466 |
| Parasponia andersonii | 3476 | plant | False | 23316 | 12017203 |
| Parastagonospora nodorum | 321614 | fungi | False | 10592 | 1257460 |
| Parastrongyloides trichosuri | 131310 | metazoa | False | 10560 | 4779590 |
| Paroedura picta | 143630 | metazoa | False | 25027 | 25782418 |
| Parus major | 9157 | metazoa | False | 14719 | 3155417 |
| Patagioenas fasciata | 372326 | metazoa | False | 16051 | 10794213 |

Continued on next page

**Table 12 – continued from previous page**

| Name | Taxon ID | Kingdom | Seed | Number of Nodes | Number of Edges |
|---|---|---|---|---|---|
| Pavo cristatus | 9049 | metazoa | False | 17844 | 7405403 |
| Paxillus rubicundulus | 930991 | fungi | False | 11361 | 6996902 |
| Pediculus humanus | 121224 | metazoa | False | 9271 | 1352494 |
| Pelodiscus sinensis | 13735 | metazoa | False | 17962 | 4075598 |
| Penaeus vannamei | 6689 | metazoa | False | 17607 | 3005289 |
| Penicilliopsis zonata | 1073090 | fungi | False | 8745 | 4143592 |
| Penicillium antarcticum | 416450 | fungi | False | 10067 | 4594154 |
| Penicillium arizonense | 1835702 | fungi | False | 10865 | 5139302 |
| Penicillium brasilianum | 104259 | fungi | False | 10621 | 6090584 |
| Penicillium camemberti | 1429867 | fungi | False | 11901 | 6958060 |
| Penicillium coprophilum | 36646 | fungi | False | 8373 | 3822600 |
| Penicillium decumbens | 69771 | fungi | False | 6656 | 2490769 |
| Penicillium digitatum | 1170229 | fungi | False | 8189 | 3431586 |
| Penicillium expansum | 27334 | fungi | False | 10210 | 5524076 |
| Penicillium flavigenum | 254877 | fungi | False | 9763 | 4150678 |
| Penicillium freii | 48697 | fungi | False | 10305 | 6111722 |
| Penicillium griseofulvum | 5078 | fungi | False | 9082 | 4473183 |
| Penicillium italicum | 40296 | fungi | False | 8865 | 3800883 |
| Penicillium nalgiovense | 60175 | fungi | False | 10218 | 5434817 |
| Penicillium nordicum | 229535 | fungi | False | 10118 | 5437478 |
| Penicillium oxalicum | 933388 | fungi | False | 8217 | 3543959 |
| Penicillium polonicum | 60169 | fungi | False | 9717 | 4582323 |
| Penicillium roqueforti | 1365484 | fungi | False | 10165 | 4971830 |
| Penicillium rubens | 500485 | fungi | False | 10506 | 1351348 |
| Penicillium solitum | 60172 | fungi | False | 10865 | 5215177 |
| Penicillium sp. occitanis | 290292 | fungi | False | 10474 | 4578387 |
| Penicillium steckii | 303698 | fungi | False | 9485 | 4867320 |
| Penicillium subrubescens | 1316194 | fungi | False | 11997 | 6871643 |
| Penicillium vulpinum | 29845 | fungi | False | 9376 | 4065906 |
| Peniophora sp. CONT | 1314672 | fungi | False | 12394 | 6535298 |
| Perca flavescens | 8167 | metazoa | False | 20954 | 20435290 |
| Periconia macrospinosa | 97972 | fungi | False | 14060 | 6970975 |
| Periophthalmus magnuspinnatus | 409849 | metazoa | False | 21899 | 20693728 |
| Peromyscus maniculatus | 230844 | metazoa | False | 21226 | 20986126 |
| Peronospora effusa | 542832 | protists | False | 6415 | 1879394 |
| Pestalotiopsis fici | 1229662 | fungi | False | 13500 | 1563475 |
| Petromyzon marinus | 7757 | metazoa | False | 10004 | 4662333 |
| Pezoloma ericae | 1745343 | fungi | False | 12279 | 7683695 |
| Phaeodactylum tricornutum | 556484 | protists | False | 6566 | 1193349 |
| Phanerochaete carnosa | 650164 | fungi | False | 10278 | 1478559 |
| Phascolarctos cinereus | 38626 | metazoa | False | 19636 | 6250585 |
| Phaseolus vulgaris | 3885 | plant | False | 25758 | 6888018 |
| Phasianus colchicus | 9054 | metazoa | False | 15460 | 4748839 |
| Phialemoniopsis curvata | 1093900 | fungi | False | 10728 | 5130273 |
| Phialocephala scopiformis | 149040 | fungi | False | 13800 | 1340788 |
| Phialocephala subalpina | 576137 | fungi | False | 15957 | 9906223 |
| Phialophora americana | 5601 | fungi | False | 9612 | 3719394 |
| Phialophora attae | 1664694 | fungi | False | 9835 | 3903624 |
| Phialosimplex sp. HF37 | 1960876 | fungi | False | 8109 | 2814161 |

Continued on next page

**Table 12 – continued from previous page**

| Name | Taxon ID | Kingdom | Seed | Number of Nodes | Number of Edges |
|---|---|---|---|---|---|
| Phlebia centrifuga | 98765 | fungi | False | 9778 | 4930851 |
| Phlebiopsis gigantea | 745531 | fungi | False | 9151 | 3763355 |
| Phoenix dactylifera | 42345 | plant | False | 24037 | 6142315 |
| Phycomyces blakesleeanus | 763407 | fungi | False | 9820 | 4495785 |
| Physeter catodon | 9755 | metazoa | False | 19448 | 7313229 |
| Phytophthora cactorum | 29920 | protists | False | 14172 | 8665603 |
| Phytophthora infestans | 403677 | protists | False | 12230 | 2977983 |
| Phytophthora kernoviae | 325452 | protists | False | 8186 | 3396241 |
| Phytophthora megakarya | 4795 | protists | False | 18553 | 6844719 |
| Phytophthora nicotianae | 4790 | protists | False | 11414 | 4650937 |
| Phytophthora parasitica | 761204 | protists | False | 13151 | 6304143 |
| Phytophthora ramorum | 164328 | protists | False | 12037 | 5430437 |
| Phytophthora sojae | 1094619 | protists | False | 14995 | 3077363 |
| Pichia kudriavzevii | 4909 | fungi | False | 4716 | 741057 |
| Pichia membranifaciens | 763406 | fungi | False | 4758 | 1106033 |
| Picoides pubescens | 118200 | metazoa | False | 12378 | 6804171 |
| Piliocolobus tephrosceles | 591936 | metazoa | False | 23302 | 7822104 |
| Piloderma croceum | 765440 | fungi | False | 13229 | 7393639 |
| Piromyces finnis | 1754191 | fungi | False | 8116 | 3660837 |
| Piromyces sp. E2 | 73868 | fungi | False | 9675 | 4792838 |
| Pisolithus microcarpus | 765257 | fungi | False | 12838 | 7632585 |
| Pisolithus tinctorius | 870435 | fungi | False | 12618 | 10015417 |
| Planoprotostelium fungivorum | 1890364 | protists | False | 9435 | 3667970 |
| Plasmodiophora brassicae | 37360 | protists | False | 6381 | 2144182 |
| Plasmodium berghei | 5823 | protists | False | 3389 | 742841 |
| Plasmodium knowlesi | 5851 | protists | False | 3495 | 885336 |
| Plasmodium vivax | 126793 | protists | False | 3502 | 870670 |
| Plasmodium yoelii | 73239 | protists | False | 4103 | 1025718 |
| Plasmopara halstedii | 4781 | protists | False | 8088 | 3095140 |
| Platysternon megacephalum | 55544 | metazoa | False | 18285 | 12436744 |
| Pleurotus ostreatus | 1137138 | fungi | False | 9324 | 5520409 |
| Pneumocystis jirovecii RU7 | 1408657 | fungi | False | 3403 | 979848 |
| Pneumocystis jirovecii SE8 | 1209962 | fungi | False | 3124 | 815491 |
| Pneumocystis murina | 1069680 | fungi | False | 3383 | 1001967 |
| Pochonia chlamydosporia 123 | 1052797 | fungi | False | 10785 | 6670404 |
| Pochonia chlamydosporia 170 | 1380566 | fungi | False | 11344 | 5693076 |
| Pocillopora damicornis | 46731 | metazoa | False | 17029 | 2993136 |
| Podarcis muralis | 64176 | metazoa | False | 21600 | 5632658 |
| Podospora anserina | 515849 | fungi | False | 9361 | 1075972 |
| Poecilia formosa | 48698 | metazoa | False | 23374 | 22828639 |
| Poecilia latipinna | 48699 | metazoa | False | 23359 | 21685745 |
| Poecilia mexicana | 48701 | metazoa | False | 23762 | 23200497 |
| Poecilia reticulata | 8081 | metazoa | False | 22436 | 6945467 |
| Pogona vitticeps | 103695 | metazoa | False | 19155 | 5023191 |
| Polyporus arcularius | 1314778 | fungi | False | 12372 | 7051964 |
| Polyporus brumalis | 139420 | fungi | False | 12292 | 7622136 |
| Polytolypa hystricis | 1447883 | fungi | False | 8656 | 4610802 |
| Pongo abelii | 9601 | metazoa | False | 19130 | 5819858 |
| Populus alba | 43335 | plant | False | 30378 | 22769478 |

Continued on next page

**Table 12 – continued from previous page**

| Name | Taxon ID | Kingdom | Seed | Number of Nodes | Number of Edges |
|---|---|---|---|---|---|
| Postia placenta MAD698RSB12 | 670580 | fungi | False | 9507 | 4842320 |
| Postia placenta Mad698R | 561896 | fungi | False | 6212 | 389671 |
| Powellomyces hirtus | 109895 | fungi | False | 5911 | 2056975 |
| Pristionchus pacificus | 54126 | metazoa | False | 12375 | 13504920 |
| Procavia capensis | 9813 | metazoa | False | 15818 | 11299824 |
| Prolemur simus | 1328070 | metazoa | False | 19855 | 17602679 |
| Propithecus coquereli | 379532 | metazoa | False | 17756 | 14058437 |
| Protomyces lactucaedebilis | 56484 | fungi | False | 5118 | 1587988 |
| Prunus persica | 3760 | plant | False | 23769 | 5663100 |
| Prunus yedoensis | 2094558 | plant | False | 33964 | 21305980 |
| Psathyrella aberdarensis | 2316362 | fungi | False | 11511 | 8521232 |
| Pseudocercospora eumusae | 321146 | fungi | False | 8373 | 3037166 |
| Pseudocercospora fijiensis | 383855 | fungi | False | 9451 | 1068934 |
| Pseudocercospora musae | 113226 | fungi | False | 7780 | 2426283 |
| Pseudogymnoascus destructans | 658429 | fungi | False | 7671 | 3556524 |
| Pseudogymnoascus sp. 05NY08 | 1622149 | fungi | False | 9176 | 4683493 |
| Pseudogymnoascus sp. 23342111 | 1524831 | fungi | False | 9335 | 4624409 |
| Pseudogymnoascus sp. 24MN13 | 1622150 | fungi | False | 9055 | 4481529 |
| Pseudogymnoascus sp. VKMF3557 | 1437433 | fungi | False | 8035 | 4046397 |
| Pseudogymnoascus sp. VKMF3808 | 1391699 | fungi | False | 8345 | 3540069 |
| Pseudogymnoascus sp. VKMF4246 | 1420902 | fungi | False | 7435 | 3325097 |
| Pseudogymnoascus sp. VKMF4281 | 1420906 | fungi | False | 7526 | 3597274 |
| Pseudogymnoascus sp. VKMF4513 | 1420907 | fungi | False | 7564 | 3133557 |
| Pseudogymnoascus sp. VKMF4515 | 1420909 | fungi | False | 8772 | 3938889 |
| Pseudogymnoascus sp. VKMF4516 | 1420910 | fungi | False | 8150 | 3361343 |
| Pseudogymnoascus sp. VKMF4518 | 1420913 | fungi | False | 9783 | 4807350 |
| Pseudogymnoascus sp. VKMF4519 | 1420914 | fungi | False | 9087 | 3523989 |
| Pseudogymnoascus sp. VKMF4520 | 1420915 | fungi | False | 9757 | 4900622 |
| Pseudogymnoascus sp. WSF3629 | 1622147 | fungi | False | 9601 | 4890425 |
| Pseudogymnoascus verrucosus | 342668 | fungi | False | 9615 | 4577105 |
| Pseudomassariella vexata | 1141098 | fungi | False | 10259 | 4981237 |
| Pseudomicrostroma glucosiphilum | 1684307 | fungi | False | 5325 | 1551236 |
| Pseudonaja textilis | 8673 | metazoa | False | 18031 | 13824935 |
| Pseudozyma hubeiensis | 1305764 | fungi | False | 5603 | 1350129 |
| Psilocybe cyanescens | 93625 | fungi | False | 10742 | 8675118 |
| Pteropus alecto | 9402 | metazoa | False | 18223 | 5859709 |
| Pteropus vampyrus | 132908 | metazoa | False | 16692 | 9988116 |
| Pterula gracilis | 1884261 | fungi | False | 8659 | 3856858 |
| Puccinia coronata | 200324 | fungi | False | 13727 | 9306529 |
| Puccinia graminis | 418459 | fungi | False | 8840 | 1544892 |
| Puccinia sorghi | 27349 | fungi | False | 10036 | 4597276 |
| Puccinia striiformis | 1165861 | fungi | False | 11002 | 7446166 |
| Puccinia triticina | 630390 | fungi | False | 11645 | 6357624 |
| Pundamilia nyererei | 303518 | metazoa | False | 23218 | 22830446 |
| Punica granatum | 22663 | plant | False | 32571 | 31040484 |
| Purpureocillium lilacinum | 33203 | fungi | False | 9337 | 1186500 |
| Pygocentrus nattereri | 42514 | metazoa | False | 26147 | 17449979 |
| Pygoscelis adeliae | 9238 | metazoa | False | 11851 | 2055766 |
| Pyrenochaeta sp. DS3sAY3a | 765867 | fungi | False | 11928 | 6679590 |

Continued on next page

**Table 12 – continued from previous page**

| Name | Taxon ID | Kingdom | Seed | Number of Nodes | Number of Edges |
|---|---|---|---|---|---|
| Pyrenophora teres | 861557 | fungi | False | 9561 | 1181131 |
| Pyrenophora triticirepentis | 426418 | fungi | False | 9426 | 3193606 |
| Pyricularia oryzae | 242507 | fungi | False | 9373 | 1116049 |
| Pyronema omphalodes | 1076935 | fungi | False | 7473 | 3104374 |
| Pyrrhoderma noxium | 2282107 | fungi | False | 7982 | 3778023 |
| Rachicladosporium antarcticum | 1507870 | fungi | False | 15699 | 11827711 |
| Rachicladosporium sp. CCFEE5018 | 1974281 | fungi | False | 15305 | 11927843 |
| Ramazzottius varieornatus | 947166 | metazoa | False | 11671 | 6031807 |
| Ramularia collocygni | 112498 | fungi | False | 9204 | 3764701 |
| Raphidocelis subcapitata | 307507 | plant | False | 10054 | 3376380 |
| Rasamsonia emersonii | 1408163 | fungi | False | 8179 | 3088608 |
| Rattus norvegicus | 10116 | metazoa | False | 21846 | 6876458 |
| Rhinocladiella mackenziei | 1442369 | fungi | False | 10252 | 5072752 |
| Rhinolophus ferrumequinum | 59479 | metazoa | False | 19176 | 18699072 |
| Rhinopithecus bieti | 61621 | metazoa | False | 19885 | 6556010 |
| Rhinopithecus roxellana | 61622 | metazoa | False | 20169 | 7236653 |
| Rhizoclosmatium globosum | 329046 | fungi | False | 9504 | 4635244 |
| Rhizoctonia solani 123E | 1423351 | fungi | False | 9236 | 3921586 |
| Rhizoctonia solani AG1IA | 983506 | fungi | False | 6942 | 2806397 |
| Rhizoctonia solani AG1IB | 1108050 | fungi | False | 10157 | 3950997 |
| Rhizophagus clarus | 94130 | fungi | False | 15620 | 6765953 |
| Rhizophagus irregularis DAOM | 1432141 | fungi | False | 15511 | 5060859 |
| Rhizophagus irregularis DAOM181602DAOM | 747089 | fungi | False | 20884 | 13449555 |
| Rhizophagus sp. MUCL43196 | 1803374 | fungi | False | 14426 | 5786184 |
| Rhizopogon vesiculosus | 180088 | fungi | False | 10086 | 6643018 |
| Rhizopogon vinicolor | 1314800 | fungi | False | 11141 | 6934632 |
| Rhizopus azygosporus | 86630 | fungi | False | 12811 | 7681185 |
| Rhizopus delemar | 246409 | fungi | False | 12241 | 7295622 |
| Rhizopus microsporus | 1340429 | fungi | False | 8740 | 3874696 |
| Rhizopus stolonifer | 4846 | fungi | False | 11918 | 7132123 |
| Rhodnius prolixus | 13249 | metazoa | False | 11166 | 4406473 |
| Rhodotorula diobovata | 5288 | fungi | False | 6294 | 2328012 |
| Rhodotorula graminis | 578459 | fungi | False | 6029 | 2087826 |
| Rhodotorula sp. CCFEE5036 | 1965284 | fungi | False | 5482 | 1925867 |
| Rhodotorula sp. JG1b | 1305733 | fungi | False | 5735 | 1950712 |
| Rhodotorula taiwanensis | 741276 | fungi | False | 5969 | 1976745 |
| Rhodotorula toruloides | 5286 | fungi | False | 6195 | 2073857 |
| Rhynchosporium commune | 914237 | fungi | False | 9505 | 4184734 |
| Ricinus communis | 3988 | plant | False | 23440 | 5757766 |
| Rickenella mellea | 50990 | fungi | False | 12055 | 9852332 |
| Rodentolepis nana | 102285 | metazoa | False | 10088 | 4921052 |
| Rosa chinensis | 74649 | plant | False | 35101 | 13583152 |
| Rosellinia necatrix | 77044 | fungi | False | 10450 | 5739852 |
| Rozella allomycis | 988480 | fungi | False | 4089 | 1249458 |
| Rutstroemia sp. NJR2017a BBW | 2070414 | fungi | False | 9740 | 4079250 |
| Rutstroemia sp. NJR2017a WRK4 | 2070412 | fungi | False | 9666 | 3421385 |
| Saccharomyces kudriavzevii | 226230 | fungi | False | 3580 | 461698 |
| Saimiri boliviensis | 39432 | metazoa | False | 18929 | 6627837 |
| Saitoella complicata | 698492 | fungi | False | 5436 | 1835349 |

Continued on next page

**Table 12 – continued from previous page**

| Name | Taxon ID | Kingdom | Seed | Number of Nodes | Number of Edges |
|---|---|---|---|---|---|
| Saitozyma podzolica | 1890683 | fungi | False | 8109 | 2385929 |
| Salarias fasciatus | 181472 | metazoa | False | 24452 | 28752421 |
| Salmo trutta | 8032 | metazoa | False | 43121 | 91339575 |
| Salpingoeca rosetta | 946362 | other | False | 8300 | 2133843 |
| Salvator merianae | 96440 | metazoa | False | 18898 | 14099940 |
| Salvia splendens | 180675 | plant | False | 49731 | 80547337 |
| Saprolegnia diclina | 1156394 | protists | False | 11697 | 6573347 |
| Saprolegnia parasitica | 695850 | protists | False | 13281 | 5328768 |
| Sarcophilus harrisii | 9305 | metazoa | False | 18072 | 4605206 |
| Scheffersomyces stipitis | 322104 | fungi | False | 5400 | 793284 |
| Schistocephalus solidus | 70667 | metazoa | False | 10886 | 5438868 |
| Schistosoma bovis | 6184 | metazoa | False | 10051 | 5092531 |
| Schistosoma curassoni | 6186 | metazoa | False | 15673 | 7995734 |
| Schistosoma haematobium | 6185 | metazoa | False | 9358 | 2088873 |
| Schistosoma japonicum | 6182 | metazoa | False | 7988 | 3212270 |
| Schistosoma mansoni | 6183 | metazoa | False | 11825 | 3717296 |
| Schistosoma margrebowiei | 48269 | metazoa | False | 15321 | 6858358 |
| Schistosoma mattheei | 31246 | metazoa | False | 16124 | 10115351 |
| Schistosoma rodhaini | 6188 | metazoa | False | 15259 | 8649098 |
| Schizophyllum commune | 578458 | fungi | False | 9134 | 1279357 |
| Schizopora paradoxa | 27342 | fungi | False | 10381 | 6132143 |
| Schizosaccharomyces cryophilus | 653667 | fungi | False | 4781 | 1208768 |
| Schizosaccharomyces japonicus | 402676 | fungi | False | 4524 | 1223519 |
| Scleroderma citrinum | 1036808 | fungi | False | 13093 | 9559337 |
| Scleropages formosus | 113540 | metazoa | False | 23583 | 9186885 |
| Sclerotinia borealis | 1432307 | fungi | False | 8419 | 3332729 |
| Sclerotinia sclerotiorum | 665079 | fungi | False | 9128 | 945635 |
| Scophthalmus maximus | 52904 | metazoa | False | 20572 | 20985136 |
| Scyliorhinus torazame | 75743 | metazoa | False | 23802 | 26510411 |
| Scytalidium lignicola | 5539 | fungi | False | 11131 | 5515910 |
| Serendipita indica | 1109443 | fungi | False | 7855 | 3928605 |
| Serendipita vermifera | 933852 | fungi | False | 9942 | 6570469 |
| Serinus canaria | 9135 | metazoa | False | 14790 | 3272926 |
| Seriola dumerili | 41447 | metazoa | False | 22809 | 7753960 |
| Seriola lalandi | 1841481 | metazoa | False | 24405 | 8212437 |
| Serpula lacrymans | 936435 | fungi | False | 9134 | 4212179 |
| Setaria italica | 4555 | plant | False | 39573 | 15629484 |
| Sinocyclocheilus anshuiensis | 1608454 | metazoa | False | 43211 | 31407409 |
| Sinocyclocheilus grahami | 75366 | metazoa | False | 43858 | 32128483 |
| Sinocyclocheilus rhinocerous | 307959 | metazoa | False | 44346 | 33815265 |
| Sistotremastrum niveocremeum | 1314777 | fungi | False | 9185 | 4773630 |
| Sistotremastrum suecicum | 1314776 | fungi | False | 8882 | 4800162 |
| Smittium culicis | 133412 | fungi | False | 7142 | 3392364 |
| Smittium megazygosporum | 133381 | fungi | False | 5200 | 1530019 |
| Smittium mucronatum | 133383 | fungi | False | 5135 | 1419102 |
| Smittium simulii | 133385 | fungi | False | 4735 | 1325652 |
| Sodiomyces alkalinus | 1314773 | fungi | False | 7359 | 2245336 |
| Solanum lycopersicum | 4081 | plant | False | 28716 | 8696921 |
| Sordaria macrospora | 771870 | fungi | False | 8407 | 1088391 |

Continued on next page

**Table 12 – continued from previous page**

| Name | Taxon ID | Kingdom | Seed | Number of Nodes | Number of Edges |
|---|---|---|---|---|---|
| Sorex araneus | 42254 | metazoa | False | 12963 | 8130008 |
| Sorghum bicolor | 4558 | plant | False | 26423 | 7936012 |
| Sparassis crispa | 139825 | fungi | False | 9854 | 4681237 |
| Sparus aurata | 8175 | metazoa | False | 24534 | 24923004 |
| Spathaspora passalidarum | 619300 | fungi | False | 5402 | 655967 |
| Spathaspora sp. JA1 | 2028339 | fungi | False | 4930 | 1289890 |
| Spermophilus dauricus | 99837 | metazoa | False | 20642 | 19422492 |
| Sphaceloma murrayae | 2082308 | fungi | False | 7520 | 2730639 |
| Sphaeramia orbicularis | 375764 | metazoa | False | 24168 | 26161145 |
| Sphaeroforma arctica | 667725 | other | False | 8015 | 2735076 |
| Sphaerulina musiva | 692275 | fungi | False | 8235 | 3831821 |
| Sphenodon punctatus | 8508 | metazoa | False | 17522 | 14449445 |
| Spizellomyces punctatus | 645134 | fungi | False | 7030 | 2731705 |
| Sporisorium scitamineum | 49012 | fungi | False | 6352 | 1983062 |
| Sporothrix insectorum | 1081102 | fungi | False | 8582 | 3848814 |
| Sporothrix schenckii | 1391915 | fungi | False | 8037 | 3144803 |
| Spraguea lophii | 1358809 | fungi | False | 1436 | 179707 |
| Stachybotrys chlorohalonata | 1283841 | fungi | False | 9430 | 4056485 |
| Stagonospora sp. SRC1lsM3a | 765868 | fungi | False | 11597 | 6421711 |
| Steccherinum ochraceum | 92696 | fungi | False | 9371 | 4467461 |
| Stegastes partitus | 144197 | metazoa | False | 22629 | 25919250 |
| Stegodyphus mimosarum | 407821 | metazoa | False | 18619 | 14265164 |
| Steinernema carpocapsae | 34508 | metazoa | False | 14049 | 9381676 |
| Stemphylium lycopersici | 183478 | fungi | False | 8083 | 2278689 |
| Stomoxys calcitrans | 35570 | metazoa | False | 12505 | 6714989 |
| Strigamia maritima | 126957 | metazoa | False | 11410 | 4974460 |
| Strigops habroptila | 2489341 | metazoa | False | 15822 | 11255391 |
| Strongylocentrotus purpuratus | 7668 | metazoa | False | 25181 | 5291309 |
| Strongyloides papillosus | 174720 | metazoa | False | 12530 | 6421735 |
| Strongyloides ratti | 34506 | metazoa | False | 7847 | 3422515 |
| Strongyloides stercoralis | 6248 | metazoa | False | 9816 | 4424240 |
| Strongyloides venezuelensis | 75913 | metazoa | False | 11332 | 6500222 |
| Struthio camelus | 441894 | metazoa | False | 14027 | 7718172 |
| Stylophora pistillata | 50429 | metazoa | False | 18793 | 2809863 |
| Suhomyces tanzawaensis | 984487 | fungi | False | 5140 | 1326833 |
| Suillus luteus | 930992 | fungi | False | 11989 | 6904006 |
| Suricata suricatta | 37032 | metazoa | False | 19103 | 5683960 |
| Syncephalastrum racemosum | 13706 | fungi | False | 8576 | 3385845 |
| Synchytrium endobioticum | 286115 | fungi | False | 5206 | 1645900 |
| Synchytrium microbalum | 1806994 | fungi | False | 5865 | 1543049 |
| Syphacia muris | 451379 | metazoa | False | 8522 | 3348980 |
| Taenia asiatica | 60517 | metazoa | False | 8043 | 3555256 |
| Taeniopygia guttata | 59729 | metazoa | False | 16268 | 3976395 |
| Takifugu rubripes | 31033 | metazoa | False | 20881 | 6745796 |
| Talaromyces amestolkiae | 1196081 | fungi | False | 9846 | 4141648 |
| Talaromyces atroroseus | 1441469 | fungi | False | 8923 | 3474406 |
| Talaromyces islandicus | 28573 | fungi | False | 9248 | 4136413 |
| Talaromyces marneffei | 441960 | fungi | False | 9397 | 1224711 |
| Talaromyces stipitatus | 441959 | fungi | False | 11297 | 5004761 |

Continued on next page

Table 12 – continued from previous page

| Name | Taxon ID | Kingdom | Seed | Number of Nodes | Number of Edges |
|---|---|---|---|---|---|
| Taphrina deformans | 1097556 | fungi | False | 4061 | 821078 |
| Temnothorax longispinosus | 300112 | metazoa | False | 9662 | 3866205 |
| Terfezia boudieri | 1051890 | fungi | False | 7327 | 3785542 |
| Termitomyces sp. J132 | 1306850 | fungi | False | 9303 | 3592938 |
| Terrapene carolina | 2587831 | metazoa | False | 21603 | 21132079 |
| Testicularia cyperi | 1882483 | fungi | False | 5342 | 1678492 |
| Tetradesmus obliquus | 3088 | plant | False | 10735 | 3042919 |
| Tetrahymena thermophila | 312017 | protists | False | 12463 | 3289164 |
| Tetranychus urticae | 32264 | metazoa | False | 10447 | 1870836 |
| Tetraodon nigroviridis | 99883 | metazoa | False | 19416 | 5279067 |
| Tetrapisispora blattae | 1071380 | fungi | False | 5041 | 925468 |
| Tetrapisispora phaffii | 1071381 | fungi | False | 4965 | 918178 |
| Thamnocephalis sphaerospora | 78915 | fungi | False | 5204 | 1418833 |
| Thecamonas trahens | 461836 | protists | False | 7153 | 2231061 |
| Theileria annulata | 5874 | protists | False | 2606 | 729206 |
| Theileria equi | 1537102 | protists | False | 3013 | 799496 |
| Theileria orientalis | 869250 | protists | False | 2516 | 647080 |
| Theileria parva | 5875 | protists | False | 2731 | 772809 |
| Thelazia callipaeda | 103827 | metazoa | False | 8880 | 4183230 |
| Theobroma cacao | 3641 | plant | False | 23049 | 4786032 |
| Thermothelomyces thermophilus | 573729 | fungi | False | 7931 | 1038702 |
| Thermothielavioides terrestris | 578455 | fungi | False | 8438 | 1038922 |
| Theropithecus gelada | 9565 | metazoa | False | 21112 | 5562982 |
| Thraustotheca clavata | 74557 | protists | False | 9910 | 4852899 |
| Tieghemostelium lacteum | 361077 | protists | False | 7071 | 1890340 |
| Tigriopus californicus | 6832 | metazoa | False | 11718 | 4636257 |
| Tilletia controversa | 13291 | fungi | False | 7031 | 2662702 |
| Tilletia walkeri | 117179 | fungi | False | 6212 | 2159424 |
| Tilletiaria anomala | 1037660 | fungi | False | 5300 | 1640726 |
| Tilletiopsis washingtonensis | 58919 | fungi | False | 5381 | 1653172 |
| Tinamus guttatus | 94827 | metazoa | False | 12751 | 5897874 |
| Tolypocladium capitatum | 45235 | fungi | False | 6966 | 2726035 |
| Tolypocladium ophioglossoides | 1163406 | fungi | False | 8301 | 3315146 |
| Tolypocladium paradoxum | 94208 | fungi | False | 8173 | 2719063 |
| Torrubiella hemipterigena | 1531966 | fungi | False | 9704 | 4434095 |
| Torulaspora delbrueckii | 1076872 | fungi | False | 4773 | 739965 |
| Toxocara canis | 6265 | metazoa | False | 12766 | 7207418 |
| Toxoplasma gondii | 432359 | protists | False | 4724 | 1461543 |
| Trachipleistophora hominis | 72359 | fungi | False | 1542 | 151957 |
| Trachymyrmex cornetzi | 471704 | metazoa | False | 13116 | 7612942 |
| Trachymyrmex septentrionalis | 34720 | metazoa | False | 10849 | 5628134 |
| Trachymyrmex zeteki | 64791 | metazoa | False | 10503 | 5343515 |
| Trametes cinnabarina | 5643 | fungi | False | 9095 | 4425598 |
| Trametes coccinea | 1353009 | fungi | False | 9786 | 6014139 |
| Trametes pubescens | 154538 | fungi | False | 10791 | 7069644 |
| Trema orientale | 63057 | plant | False | 24010 | 15447717 |
| Tremella mesenterica | 5217 | fungi | False | 5828 | 1080771 |
| Tribolium castaneum | 7070 | metazoa | False | 11961 | 1849529 |
| Trichechus manatus | 127582 | metazoa | False | 18991 | 6066337 |

Continued on next page

**Table 12 – continued from previous page**

| Name | Taxon ID | Kingdom | Seed | Number of Nodes | Number of Edges |
|---|---|---|---|---|---|
| Trichinella britovi | 45882 | metazoa | False | 7543 | 4052299 |
| Trichinella murrelli | 144512 | metazoa | False | 7097 | 2757967 |
| Trichinella nativa | 6335 | metazoa | False | 6852 | 2710751 |
| Trichinella nelsoni | 6336 | metazoa | False | 6885 | 2679041 |
| Trichinella papuae | 268474 | metazoa | False | 6244 | 1811509 |
| Trichinella patagoniensis | 990121 | metazoa | False | 7191 | 2633127 |
| Trichinella pseudospiralis | 6337 | metazoa | False | 6626 | 2246417 |
| Trichinella sp. T6 | 92179 | metazoa | False | 7231 | 2379530 |
| Trichinella sp. T8 | 92180 | metazoa | False | 7316 | 2641950 |
| Trichinella sp. T9 | 181606 | metazoa | False | 6574 | 2463496 |
| Trichinella spiralis | 6334 | metazoa | False | 6873 | 972343 |
| Trichinella zimbabwensis | 268475 | metazoa | False | 6552 | 1971608 |
| Trichobilharzia regenti | 157069 | metazoa | False | 16873 | 13520452 |
| Trichoderma arundinaceum | 490622 | fungi | False | 9535 | 3554632 |
| Trichoderma asperellum | 1042311 | fungi | False | 10025 | 4713280 |
| Trichoderma atroviride | 452589 | fungi | False | 10138 | 4879562 |
| Trichoderma gamsii | 398673 | fungi | False | 9849 | 3651243 |
| Trichoderma harzianum | 983964 | fungi | False | 11271 | 5198795 |
| Trichoderma longibrachiatum | 983965 | fungi | False | 8461 | 3583891 |
| Trichoderma reesei | 431241 | fungi | False | 8304 | 1178145 |
| Trichoderma virens | 413071 | fungi | False | 10949 | 4603246 |
| Trichomalopsis sarcophagae | 543379 | metazoa | False | 9905 | 4343335 |
| Trichomonas vaginalis | 5722 | protists | False | 14227 | 22227127 |
| Trichophyton benhamiae | 663331 | fungi | False | 6990 | 920439 |
| Trichophyton interdigitale | 1215338 | fungi | False | 7191 | 2733463 |
| Trichophyton rubrum | 559305 | fungi | False | 7375 | 3127998 |
| Trichoplax sp. H2 | 287889 | metazoa | False | 10158 | 5359227 |
| Trichosporon asahii | 1220162 | fungi | False | 6166 | 1798998 |
| Trichuris muris | 70415 | metazoa | False | 9864 | 4529738 |
| Trichuris trichiura | 36087 | metazoa | False | 8095 | 3629259 |
| Trifolium pratense | 57577 | plant | False | 40815 | 38626298 |
| Triticum aestivum | 4565 | plant | False | 89765 | 265509180 |
| Triticum urartu | 4572 | plant | False | 26964 | 40232331 |
| Tropilaelaps mercedesae | 418985 | metazoa | False | 9840 | 3952555 |
| Trypanosoma cruzi | 353153 | protists | False | 10547 | 4428447 |
| Trypanosoma theileri | 67003 | protists | False | 5688 | 1129254 |
| Tuber borchii | 42251 | fungi | False | 8522 | 5781722 |
| Tuber magnatum | 42249 | fungi | False | 7312 | 3366629 |
| Tuber melanosporum | 656061 | fungi | False | 6109 | 1055995 |
| Tubulinosema ratisbonensis | 291195 | fungi | False | 1366 | 135404 |
| Tulasnella calospora | 1051891 | fungi | False | 11817 | 9485244 |
| Tupaia belangeri | 37347 | metazoa | False | 14985 | 10363809 |
| Tupaia chinensis | 246437 | metazoa | False | 18965 | 6725785 |
| Tursiops truncatus | 9739 | metazoa | False | 16272 | 5344417 |
| Uncinocarpus reesii | 336963 | fungi | False | 6754 | 880853 |
| Urocitellus parryii | 9999 | metazoa | False | 19381 | 17307030 |
| Ursus americanus | 9643 | metazoa | False | 20245 | 6923156 |
| Ursus arctos | 116960 | metazoa | False | 19392 | 5628115 |
| Ursus maritimus | 29073 | metazoa | False | 18385 | 5919540 |

Continued on next page

**Table 12 – continued from previous page**

| Name | Taxon ID | Kingdom | Seed | Number of Nodes | Number of Edges |
|---|---|---|---|---|---|
| Ustilaginoidea virens | 1159556 | fungi | False | 6871 | 2608093 |
| Ustilago hordei | 1128400 | fungi | False | 5853 | 1886308 |
| Ustilago maydis | 237631 | fungi | False | 5888 | 898760 |
| Valsa mali | 694573 | fungi | False | 9358 | 4174929 |
| Valsa malicola | 356882 | fungi | False | 9296 | 4064339 |
| Valsa sordida | 252740 | fungi | False | 8725 | 3980413 |
| Vanderwaltozyma polyspora | 436907 | fungi | False | 5009 | 886564 |
| Varanus komodoensis | 61221 | metazoa | False | 17700 | 13388559 |
| Vavraia culicis | 948595 | fungi | False | 1457 | 171111 |
| Venturia effusa | 50376 | fungi | False | 8732 | 3674616 |
| Venturia nashicola | 86259 | fungi | False | 8658 | 3337786 |
| Venustampulla echinocandica | 2656787 | fungi | False | 9280 | 4042665 |
| Verruconis gallopava | 253628 | fungi | False | 8540 | 3701901 |
| Verticillium alfalfae | 526221 | fungi | False | 9359 | 1103758 |
| Verticillium dahliae | 498257 | fungi | False | 9502 | 1225254 |
| Verticillium longisporum | 100787 | fungi | False | 17793 | 13249795 |
| Verticillium nonalfalfae | 1051616 | fungi | False | 8807 | 2758413 |
| Vicugna pacos | 30538 | metazoa | False | 11599 | 5639350 |
| Vigna angularis | 3914 | plant | False | 25473 | 6475703 |
| Vigna radiata | 3916 | plant | False | 25826 | 6372728 |
| Vitis vinifera | 29760 | plant | False | 28092 | 4581669 |
| Vitrella brassicaformis | 1169540 | protists | False | 9820 | 4600367 |
| Vittaforma corneae | 993615 | fungi | False | 1324 | 154807 |
| Volvox carteri | 3068 | plant | False | 9821 | 2044607 |
| Vombatus ursinus | 29139 | metazoa | False | 21067 | 19566817 |
| Vulpes vulpes | 9627 | metazoa | False | 19998 | 6974892 |
| Wallemia mellicola | 671144 | fungi | False | 4573 | 677202 |
| Wickerhamiella sorbophila | 45607 | fungi | False | 4154 | 1089649 |
| Wickerhamomyces anomalus | 683960 | fungi | False | 5587 | 1636778 |
| Wickerhamomyces ciferrii | 1206466 | fungi | False | 5577 | 1539334 |
| Wolfiporia cocos | 742152 | fungi | False | 9445 | 5307101 |
| Wuchereria bancrofti | 6293 | metazoa | False | 8089 | 3195395 |
| Xenopus laevis | 8355 | metazoa | False | 32988 | 13695756 |
| Xenopus tropicalis | 8364 | metazoa | False | 19529 | 3405065 |
| Xiphophorus couchianus | 32473 | metazoa | False | 20257 | 5862427 |
| Xiphophorus maculatus | 8083 | metazoa | False | 23449 | 7821376 |
| Xylaria flabelliformis | 2512241 | fungi | False | 9922 | 4822826 |
| Xylaria grammica | 363999 | fungi | False | 10876 | 6045899 |
| Xylaria hypoxylon | 37992 | fungi | False | 10053 | 5099345 |
| Xylona heveae | 1328760 | fungi | False | 6965 | 2971633 |
| Yamadazyma tenuis | 590646 | fungi | False | 5031 | 619034 |
| Yarrowia lipolytica | 284591 | fungi | False | 5371 | 781808 |
| Zonotrichia albicollis | 44394 | metazoa | False | 13549 | 8040567 |
| Zootermopsis nevadensis | 136037 | metazoa | False | 11254 | 1457725 |
| Zostera marina | 29655 | plant | False | 17636 | 11136311 |
| Zygosaccharomyces bailii | 1333698 | fungi | False | 829 | 26840 |
| Zygosaccharomyces rouxii | 559307 | fungi | False | 4744 | 707672 |
| Zymoseptoria brevis | 1047168 | fungi | False | 8746 | 3700700 |
| Zymoseptoria tritici ST99CH3D7 | 1276538 | fungi | False | 9069 | 3409781 |

Continued on next page

**Table 12 – continued from previous page**

| Name | Taxon ID | Kingdom | Seed | Number of Nodes | Number of Edges |
|---|---|---|---|---|---|
| fungal sp. No11243 | 1603295 | fungi | False | 7414 | 2388731 |
| fungal sp. No14919 | 1813822 | fungi | False | 11459 | 7425848 |

Generated LaTeX code for supplementary figures

# 6 KEGG Curves Per Species

Suppl. Figure 5: Comparison of protein embedding methods in eukaryotic species using KEGG pathways

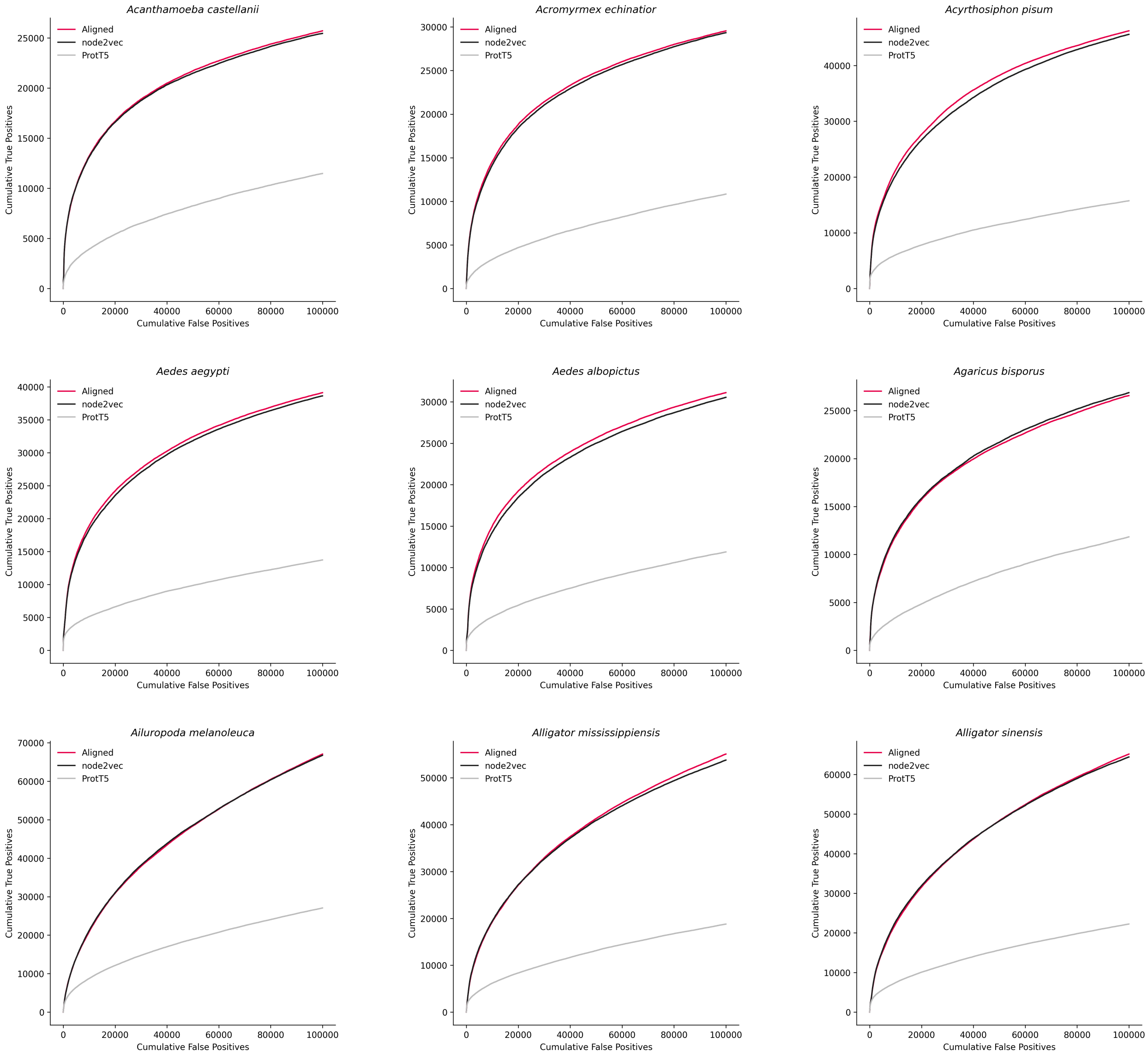

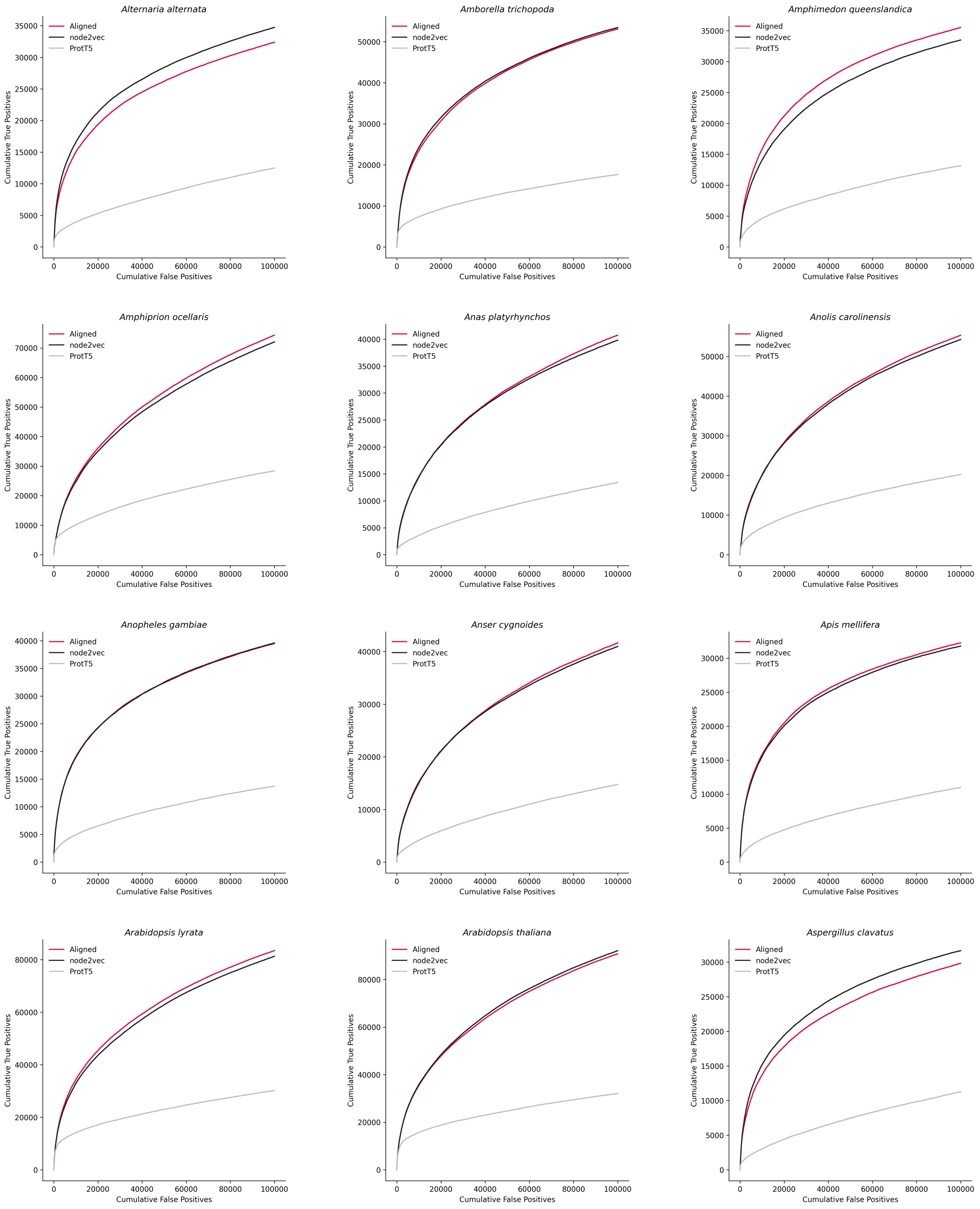
Alternaria alternata
Amborella trichopoda
Amphimedon queenslandica
Amphiprion ocellaris
Anas platyrhynchos
Anolis carolinensis
Anopheles gambiae
Anser cygnoides
Apis mellifera
Arabidopsis lyrata
Arabidopsis thaliana
Aspergillus clavatus
Aligned
node2vec
ProtT5
Cumulative True Positives
Cumulative False Positives

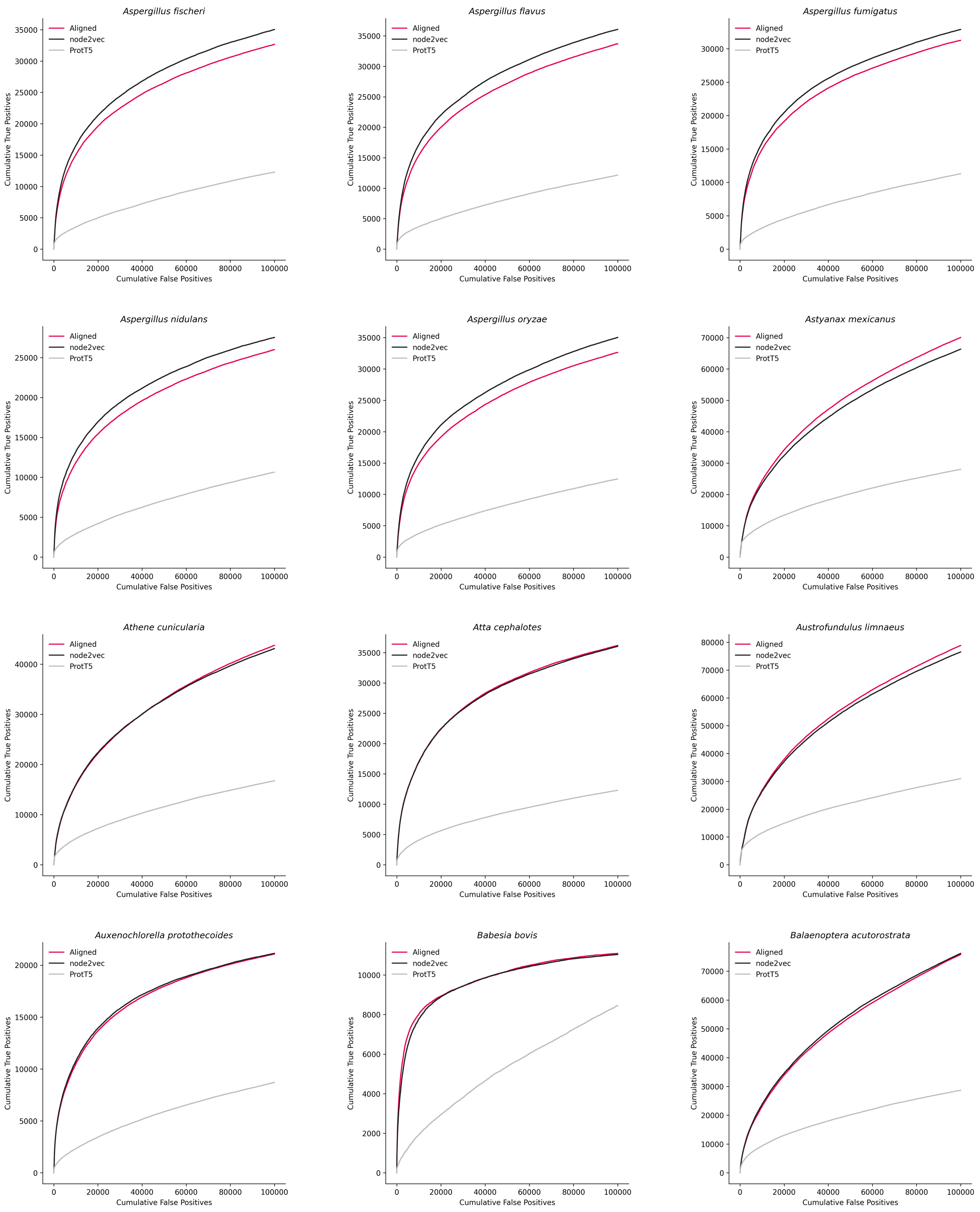
Aspergillus fischeri
Aspergillus flavus
Aspergillus fumigatus
Aspergillus nidulans
Aspergillus oryzae
Astyanax mexicanus
Athene cunicularia
Atta cephalotes
Austrofundulus limnaeus
Auxenochlorella protothecoides
Babesia bovis
Balaenoptera acutorostrata
Aligned
node2vec
ProtT5
Cumulative True Positives
Cumulative False Positives

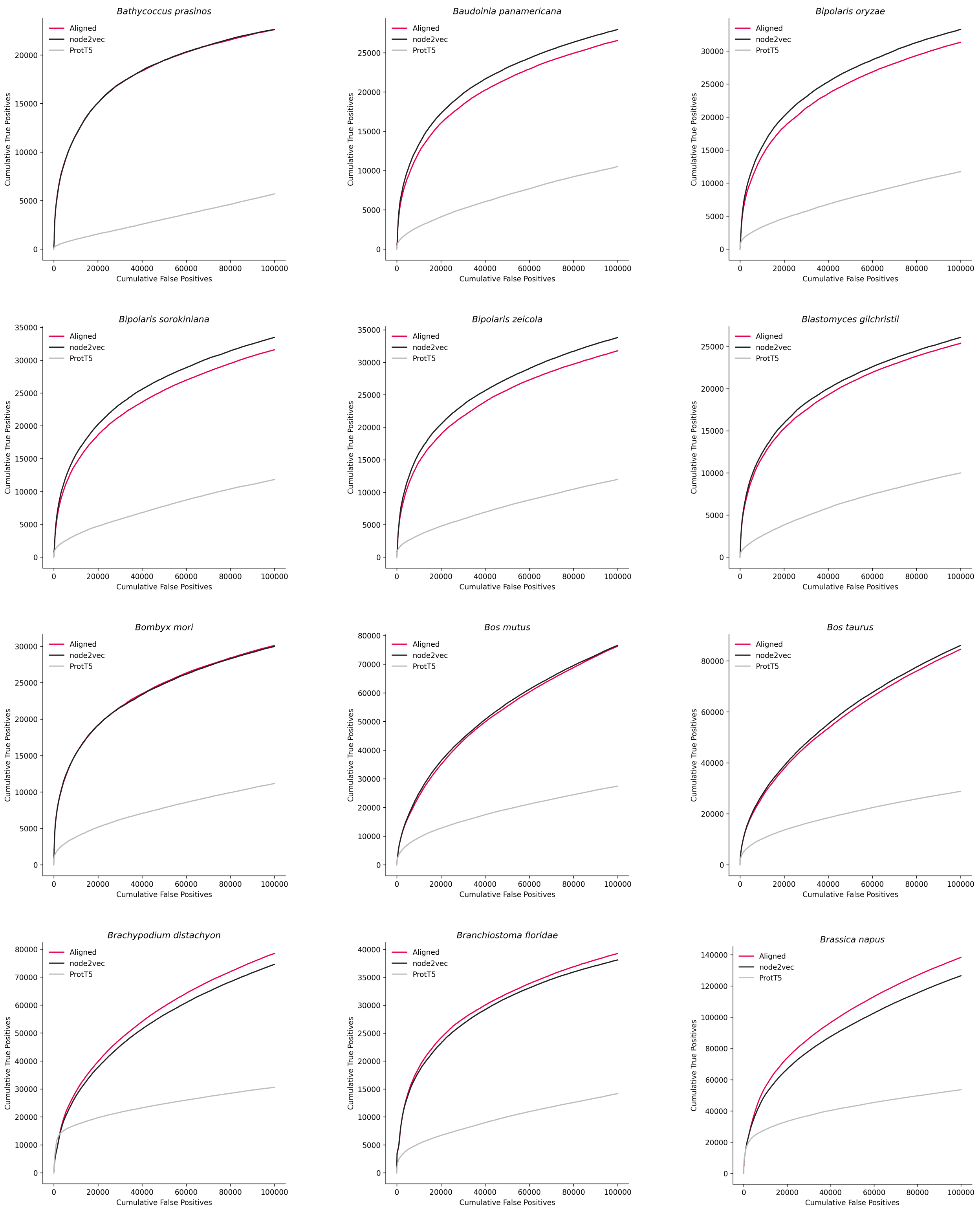
Bathycoccus prasinos
Baudoinia panamericana
Bipolaris oryzae
Bipolaris sorokiniana
Bipolaris zeicola
Blastomyces gilchristii
Bombyx mori
Bos mutus
Bos taurus
Brachypodium distachyon
Branchiostoma floridae
Brassica napus
Aligned
node2vec
ProtT5
Cumulative True Positives
Cumulative False Positives

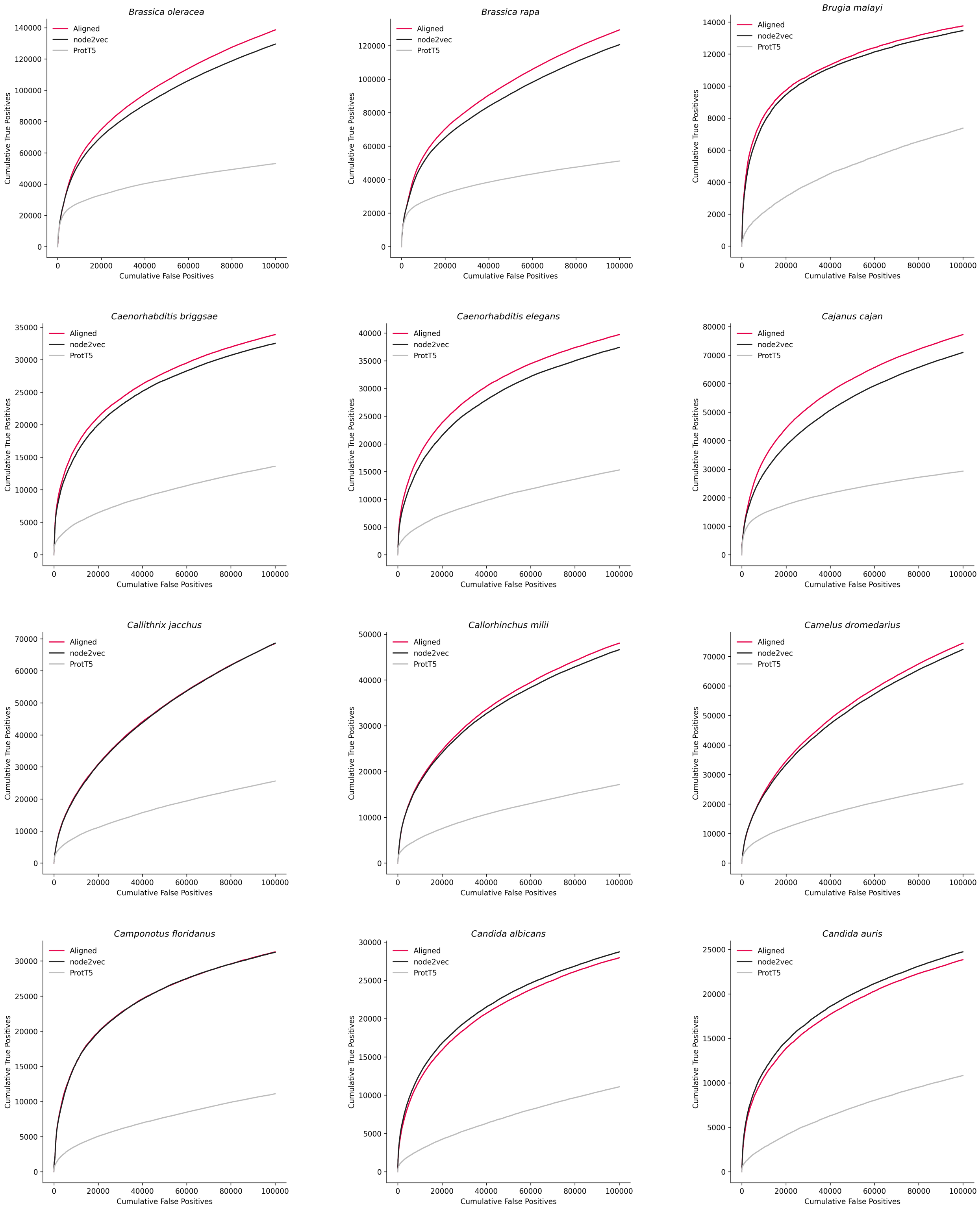
Brassica oleracea
Brassica rapa
Brugia malayi
Caenorhabditis briggsae
Caenorhabditis elegans
Cajanus cajan
Callithrix jacchus
Callorhinchus milii
Camelus dromedarius
Camponotus floridanus
Candida albicans
Candida auris
Aligned
node2vec
ProtT5
Cumulative True Positives
Cumulative False Positives

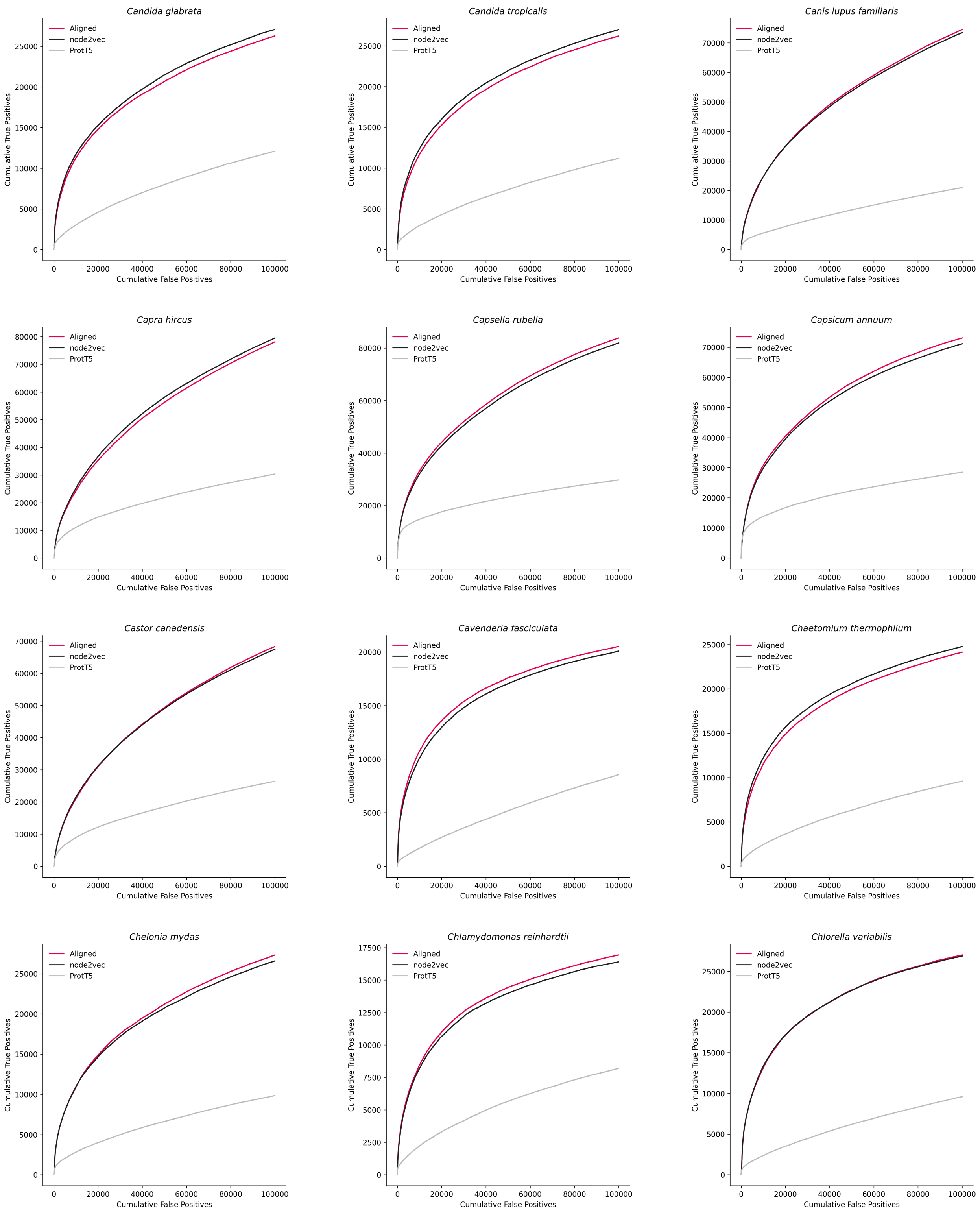
Candida glabrata
Candida tropicalis
Canis lupus familiaris
Capra hircus
Capsella rubella
Capsicum annuum
Castor canadensis
Cavenderia fasciculata
Chaetomium thermophilum
Chelonia mydas
Chlamydomonas reinhardtii
Chlorella variabilis
Aligned
node2vec
ProtT5
Cumulative True Positives
Cumulative False Positives

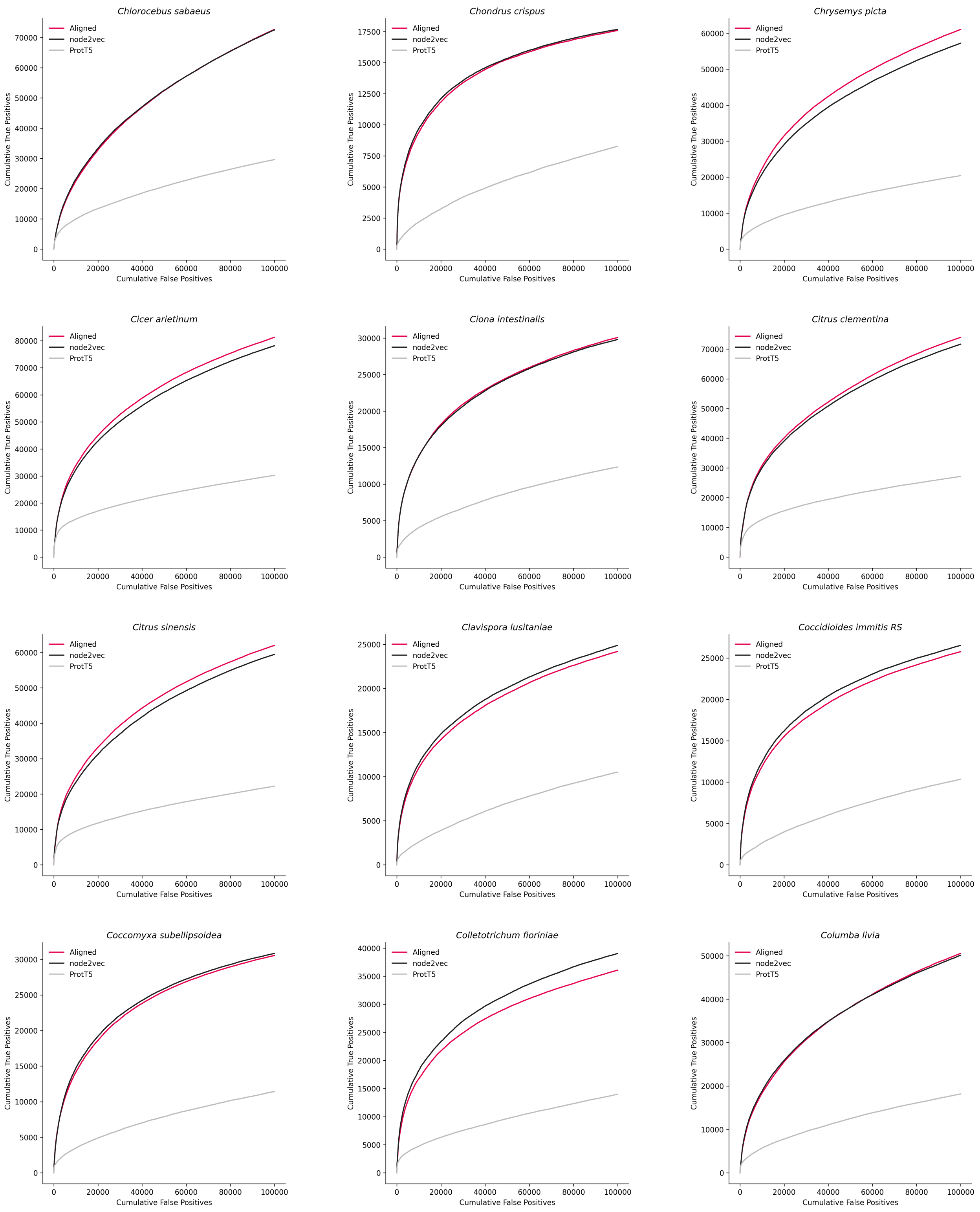
Chlorocebus sabaeus
Chondrus crispus
Chrysemys picta
Cicer arietinum
Ciona intestinalis
Citrus clementina
Citrus sinensis
Clavispora lusitaniae
Coccidioides immitis RS
Coccomyxa subellipsoidea
Colletotrichum fioriniae
Columba livia
Aligned
node2vec
ProtT5
Cumulative True Positives
Cumulative False Positives

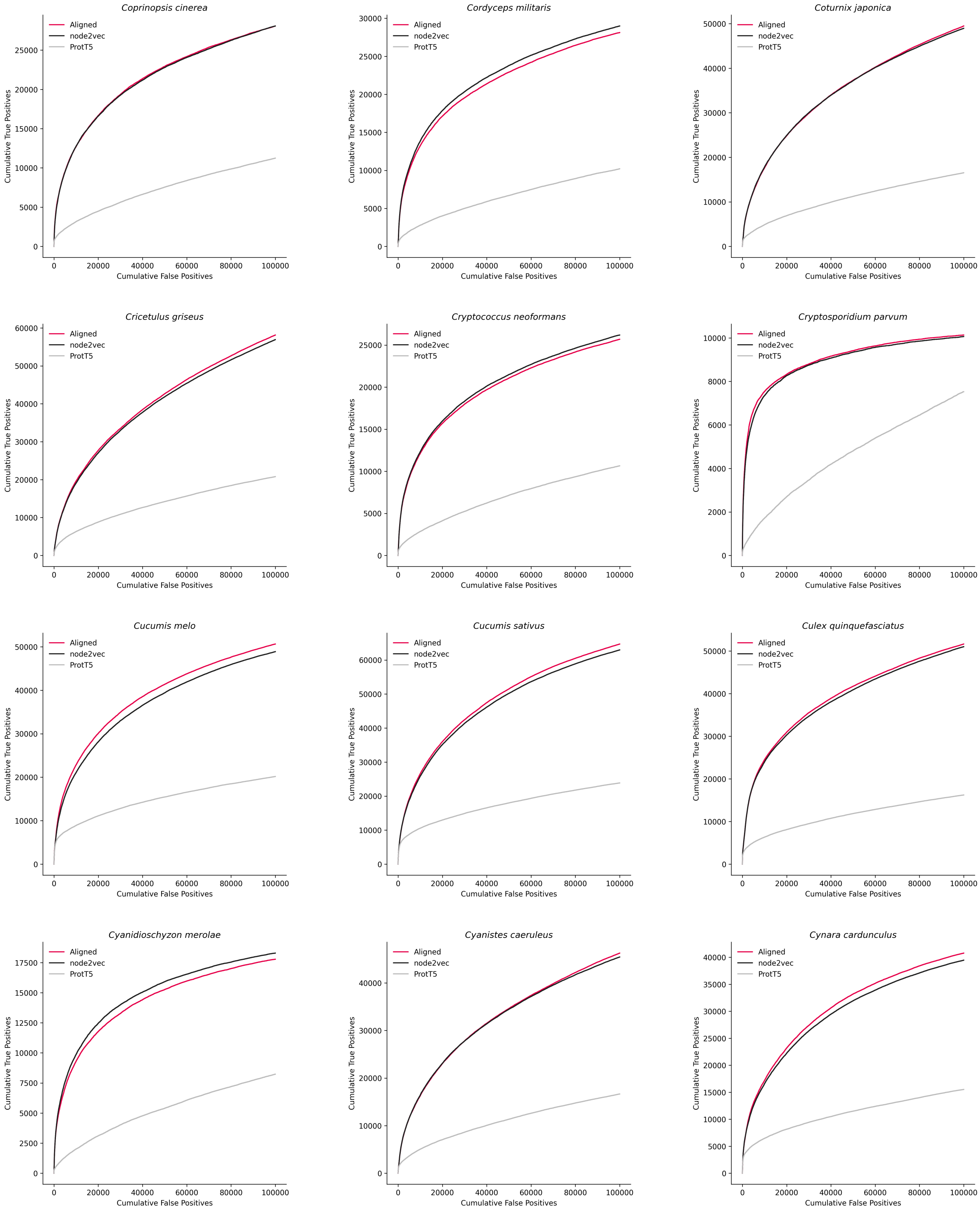
Coprinopsis cinerea
Cordyceps militaris
Coturnix japonica
Cricetulus griseus
Cryptococcus neoformans
Cryptosporidium parvum
Cucumis melo
Cucumis sativus
Culex quinquefasciatus
Cyanidioschyzon merolae
Cyanistes caeruleus
Cynara cardunculus
Aligned
node2vec
ProtT5
Cumulative True Positives
Cumulative False Positives

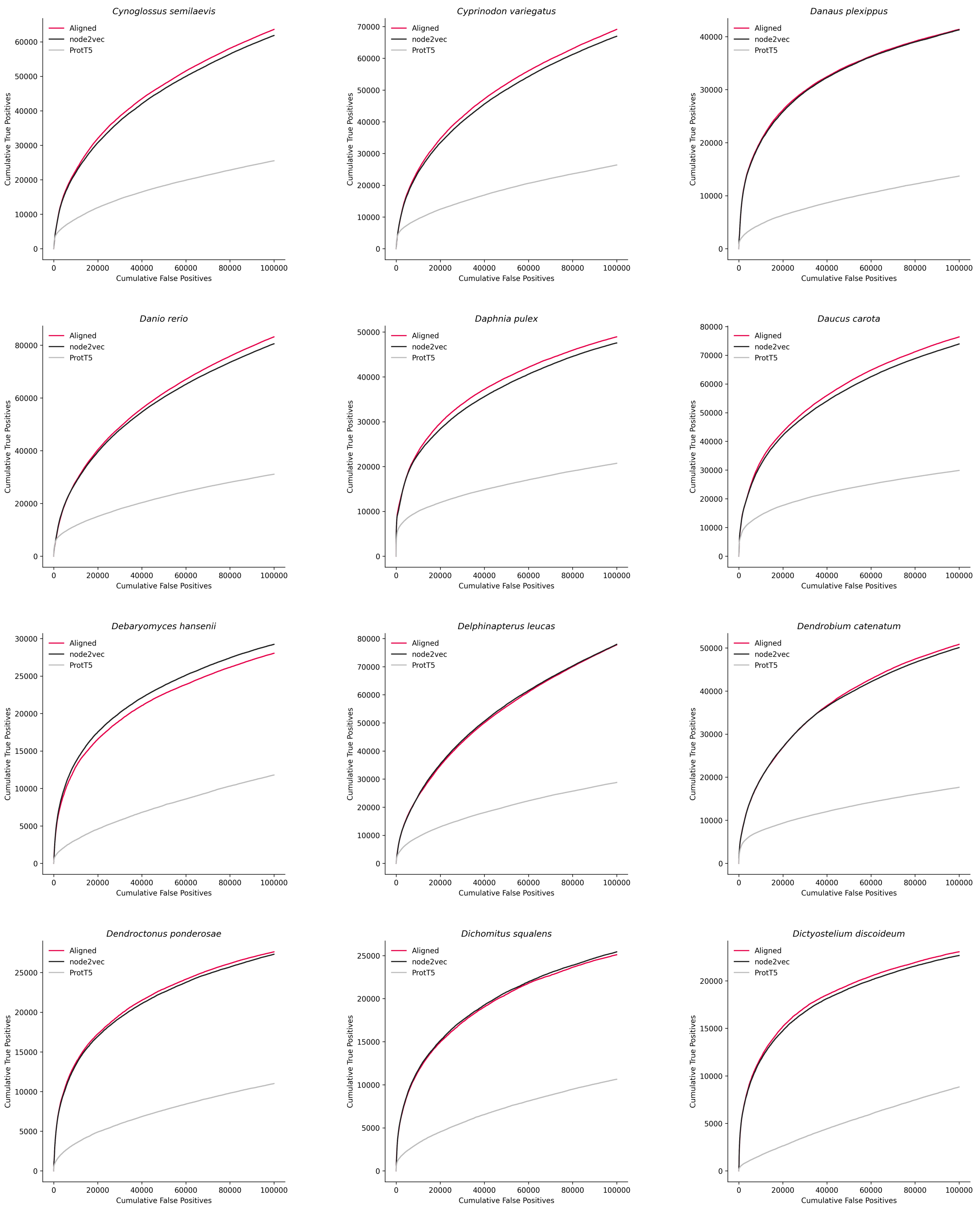

*Cynoglossus semilaevis*
*Cyprinodon variegatus*
*Danaus plexippus*
*Danio rerio*
*Daphnia pulex*
*Daucus carota*
*Debaryomyces hansenii*
*Delphinapterus leucas*
*Dendrobium catenatum*
*Dendroctonus ponderosae*
*Dichomitus squalens*
*Dictyostelium discoideum*
Aligned
node2vec
ProtT5
Cumulative True Positives
Cumulative False Positives

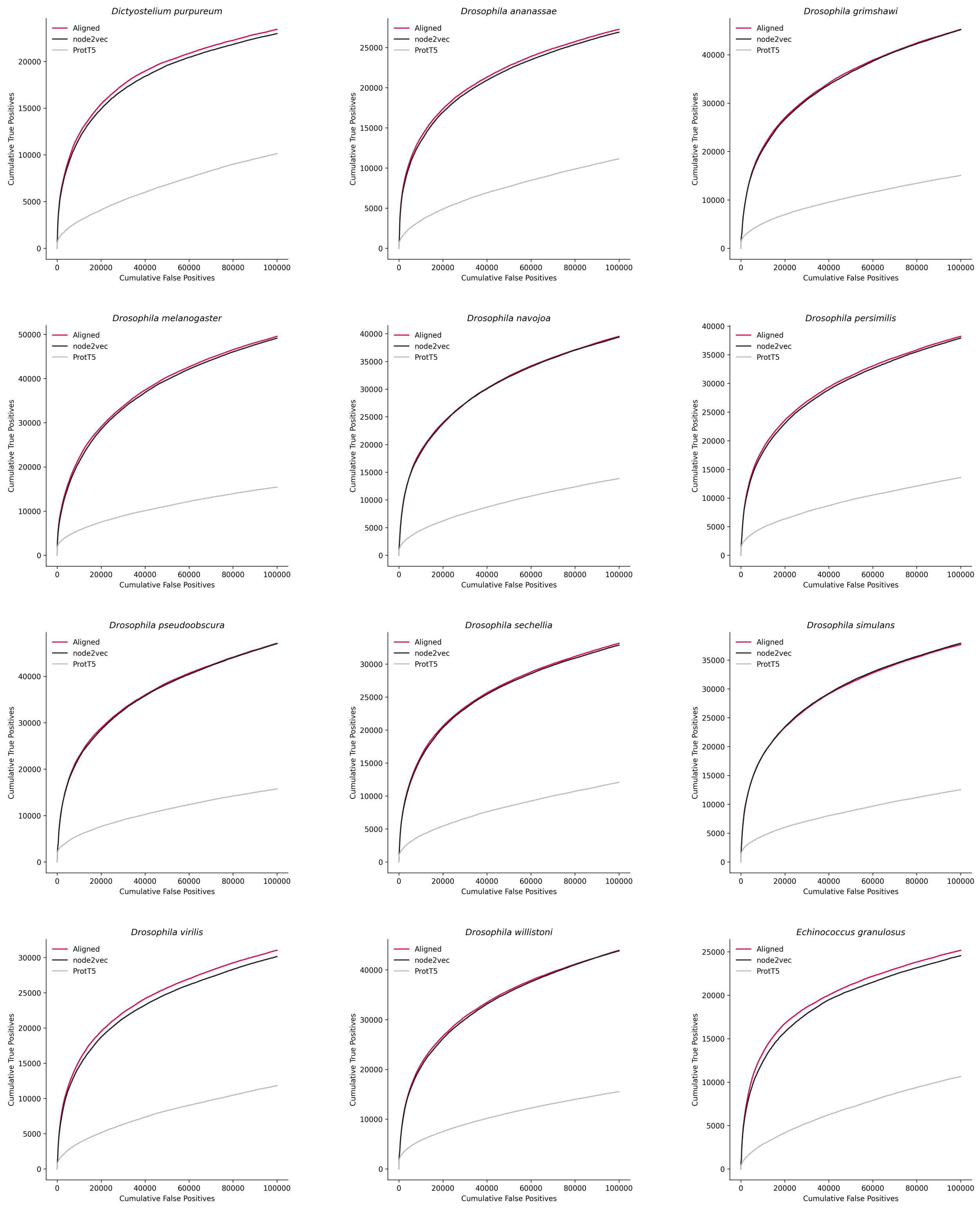
Dictyostelium purpureum
Drosophila ananassae
Drosophila grimshawi
Drosophila melanogaster
Drosophila navojoa
Drosophila persimilis
Drosophila pseudoobscura
Drosophila sechellia
Drosophila simulans
Drosophila virilis
Drosophila willistoni
Echinococcus granulosus
Aligned
node2vec
ProtT5
Cumulative True Positives
Cumulative False Positives

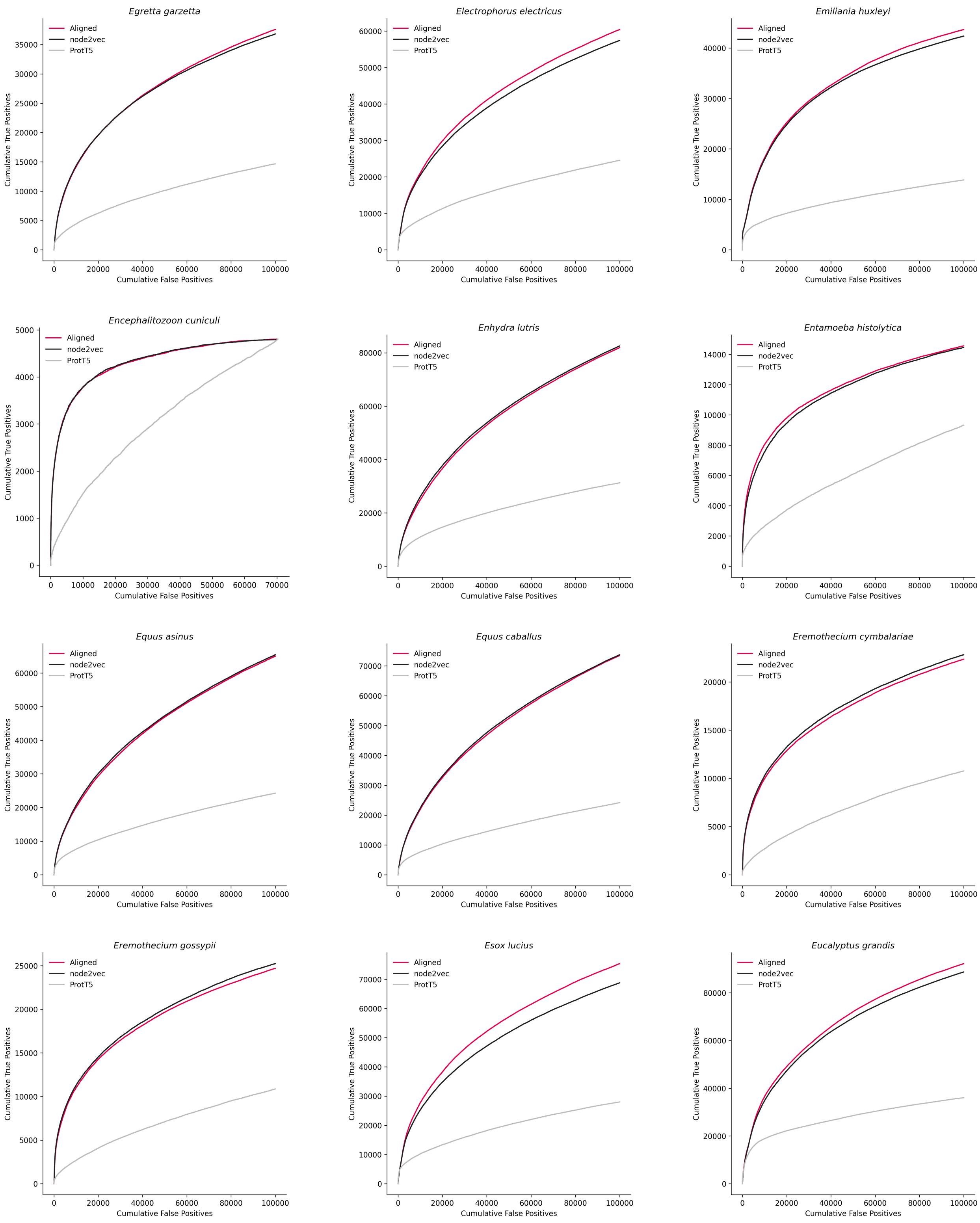
Egretta garzetta
Electrophorus electricus
Emiliania huxleyi
Encephalitozoon cuniculi
Enhydra lutris
Entamoeba histolytica
Equus asinus
Equus caballus
Eremothecium cymbalariae
Eremothecium gossypii
Esox lucius
Eucalyptus grandis
Aligned
node2vec
ProtT5
Cumulative True Positives
Cumulative False Positives

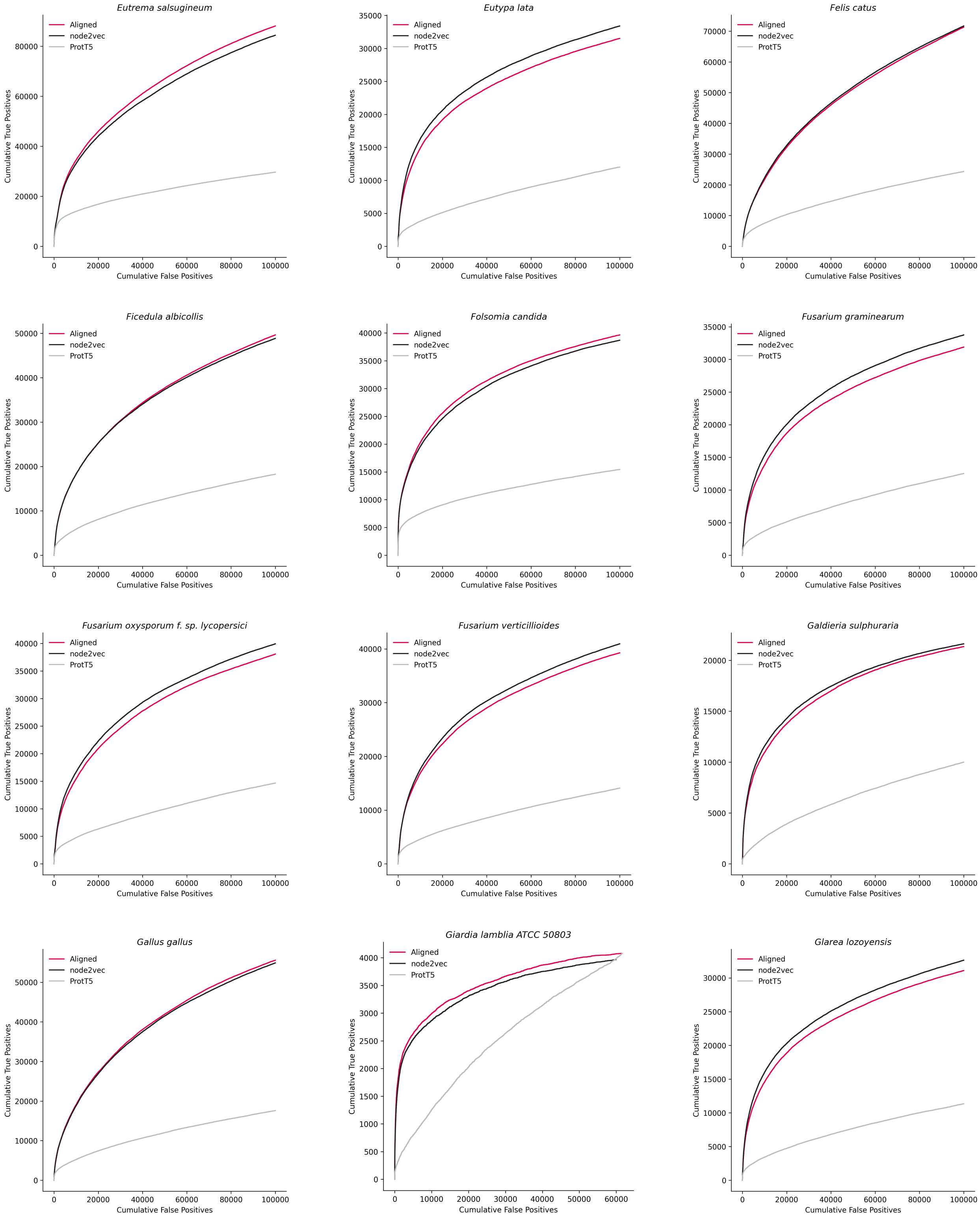

Eutrema salsugineum
Eutypa lata
Felis catus
Ficedula albicollis
Folsomia candida
Fusarium graminearum
Fusarium oxysporum f. sp. lycopersici
Fusarium verticillioides
Galdieria sulphuraria
Gallus gallus
Giardia lamblia ATCC 50803
Glarea lozoyensis
Aligned
node2vec
ProtT5
Cumulative True Positives
Cumulative False Positives

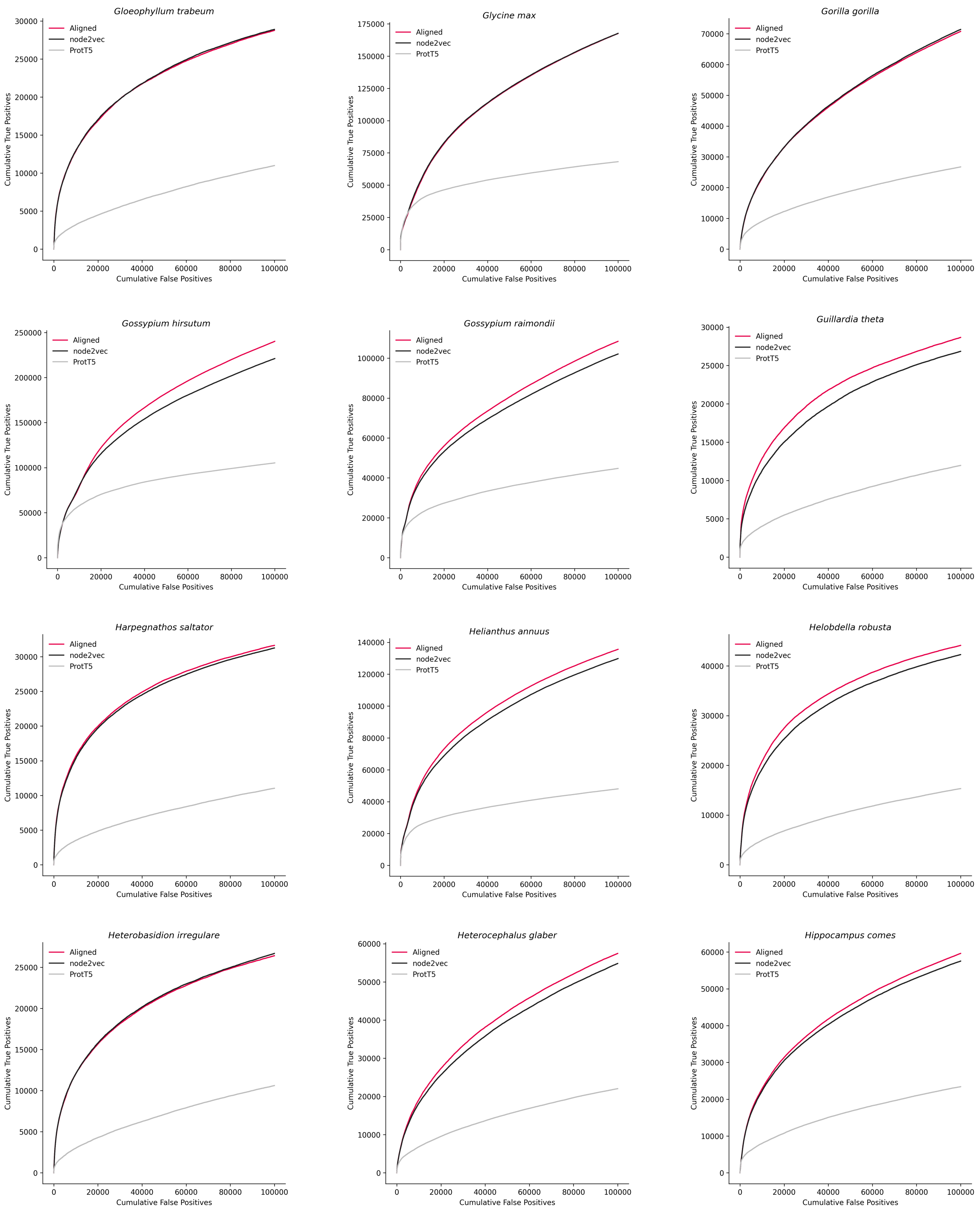
Gloeophyllum trabeum
Glycine max
Gorilla gorilla
Gossypium hirsutum
Gossypium raimondii
Guillardia theta
Harpegnathos saltator
Helianthus annuus
Helobdella robusta
Heterobasidion irregulare
Heterocephalus glaber
Hippocampus comes
Aligned
node2vec
ProtT5
Cumulative True Positives
Cumulative False Positives

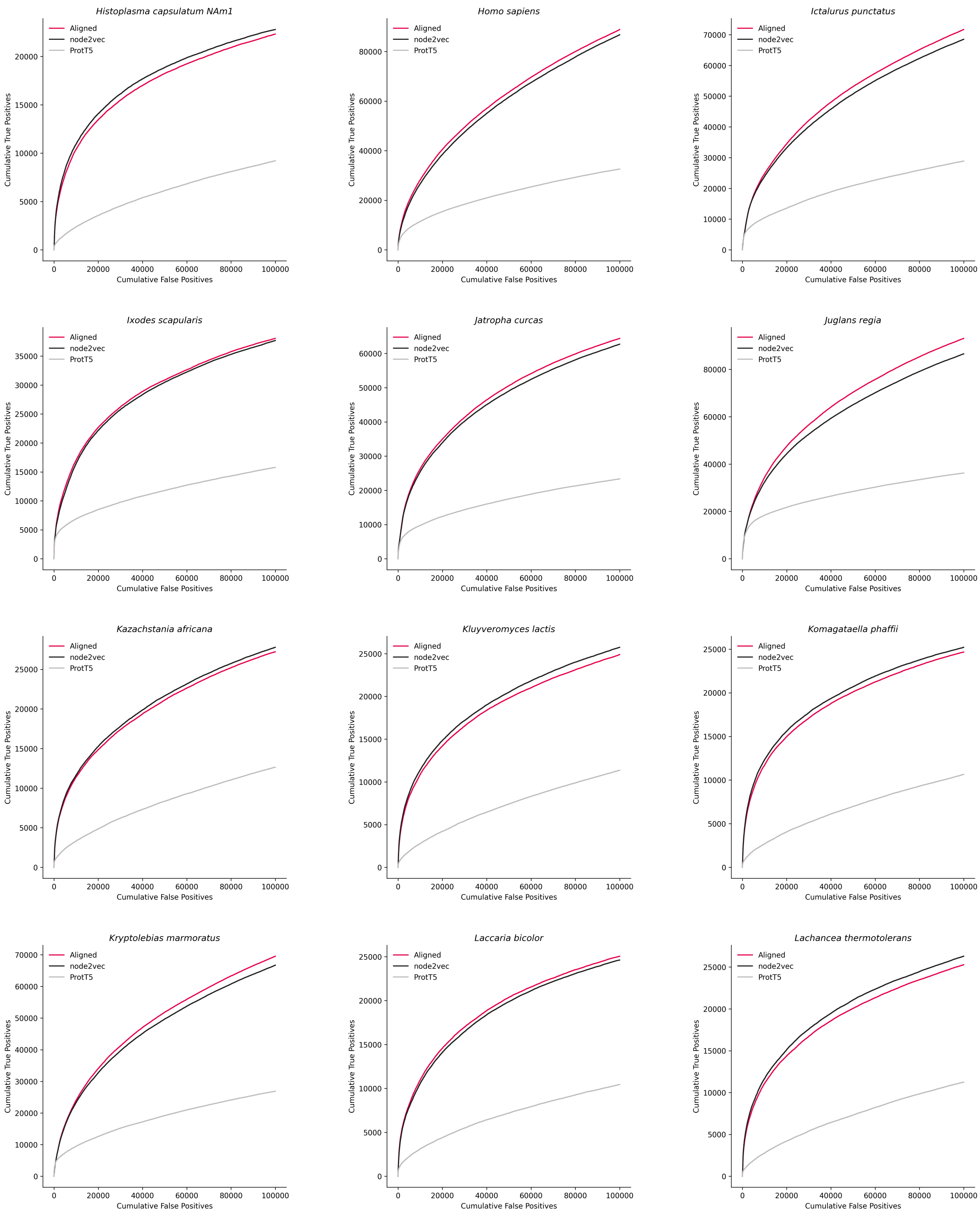
Histoplasma capsulatum NAm1
Homo sapiens
Ictalurus punctatus
Ixodes scapularis
Jatropha curcas
Juglans regia
Kazachstania africana
Kluyveromyces lactis
Komagataella phaffii
Kryptolebias marmoratus
Laccaria bicolor
Lachancea thermotolerans
Aligned
node2vec
ProtT5
Cumulative True Positives
Cumulative False Positives

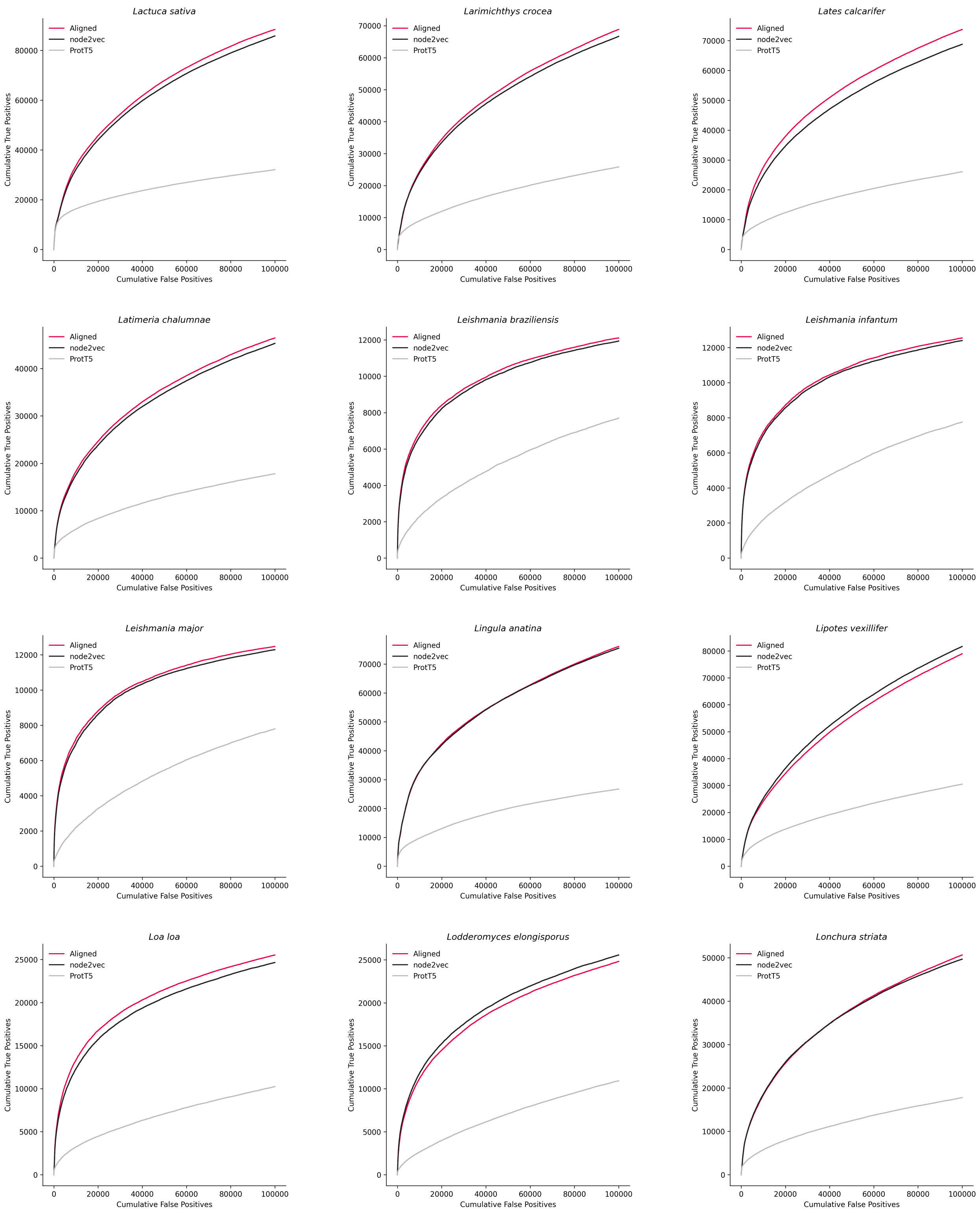
Lactuca sativa
Larimichthys crocea
Lates calcarifer
Latimeria chalumnae
Leishmania braziliensis
Leishmania infantum
Leishmania major
Lingula anatina
Lipotes vexillifer
Loa loa
Lodderomyces elongisporus
Lonchura striata
Aligned
node2vec
ProtT5
Cumulative True Positives
Cumulative False Positives

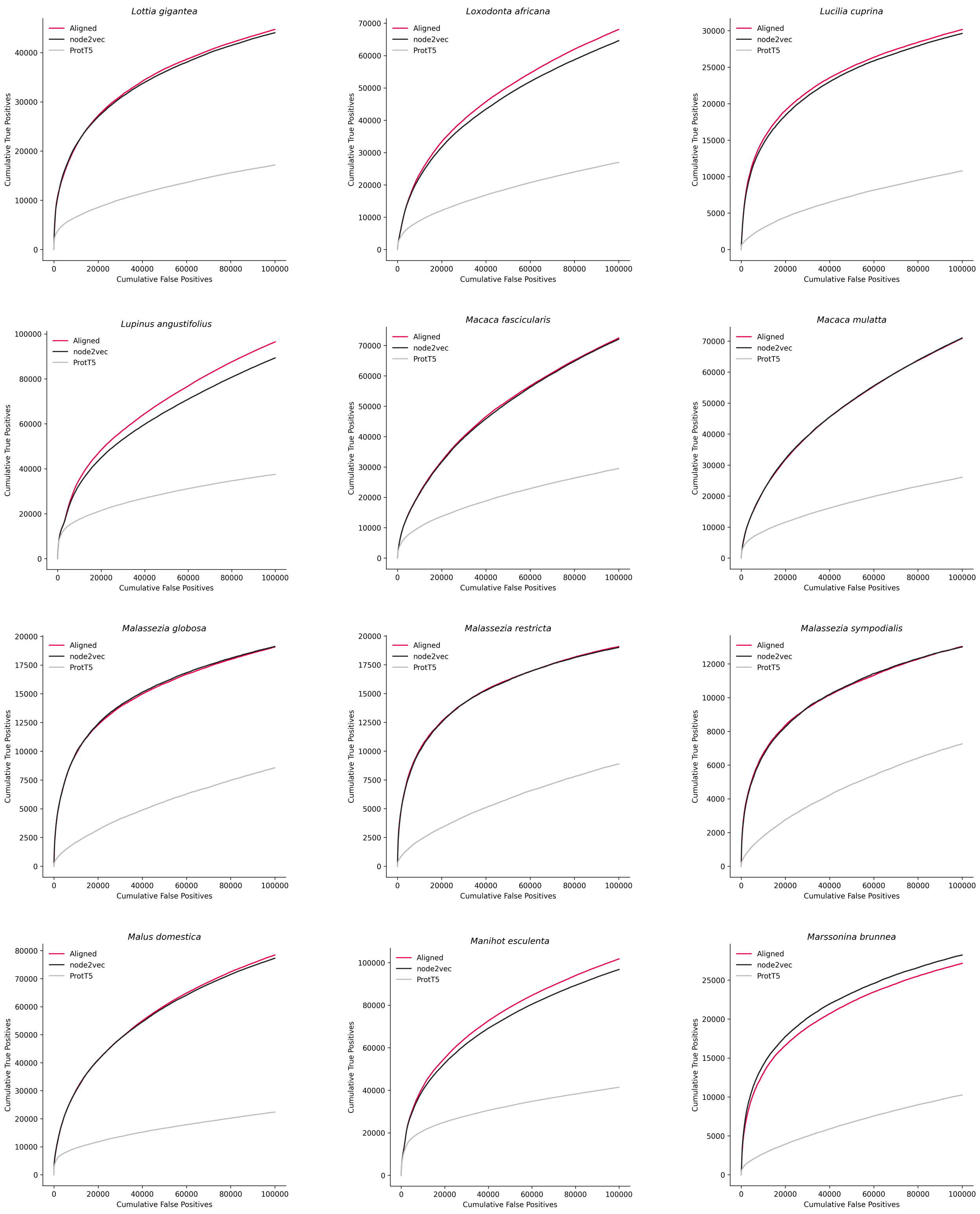

Lottia gigantea
Loxodonta africana
Lucilia cuprina
Lupinus angustifolius
Macaca fascicularis
Macaca mulatta
Malassezia globosa
Malassezia restricta
Malassezia sympodialis
Malus domestica
Manihot esculenta
Marssonina brunnea
Aligned
node2vec
ProtT5
Cumulative True Positives
Cumulative False Positives

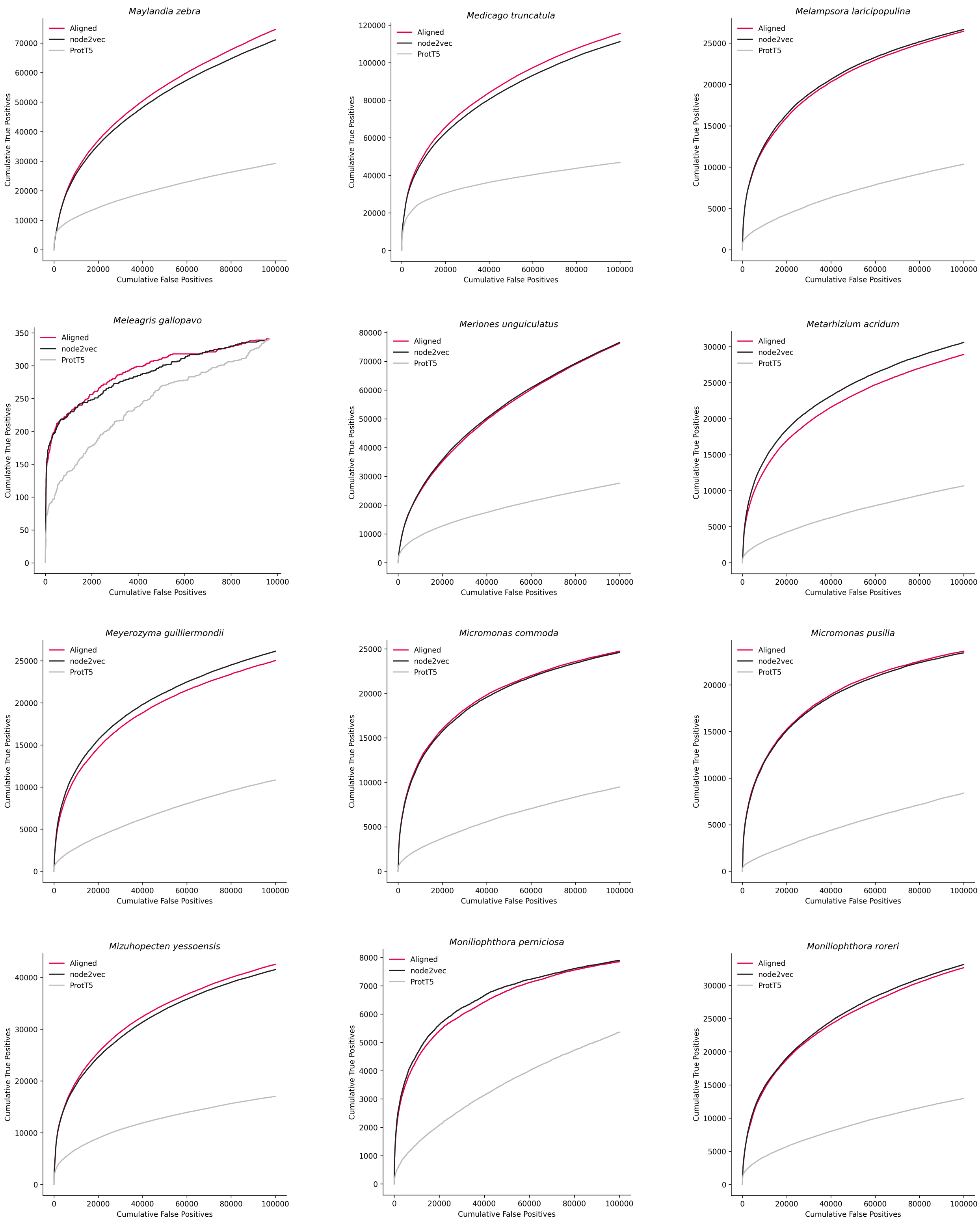
Maylandia zebra
Medicago truncatula
Melampsora laricipopulina
Meleagris gallopavo
Meriones unguiculatus
Metarhizium acridum
Meyerozyma guilliermondii
Micromonas commoda
Micromonas pusilla
Mizuhopecten yessoensis
Moniliophthora perniciosa
Moniliophthora roreri
Aligned
node2vec
ProtT5
Cumulative True Positives
Cumulative False Positives

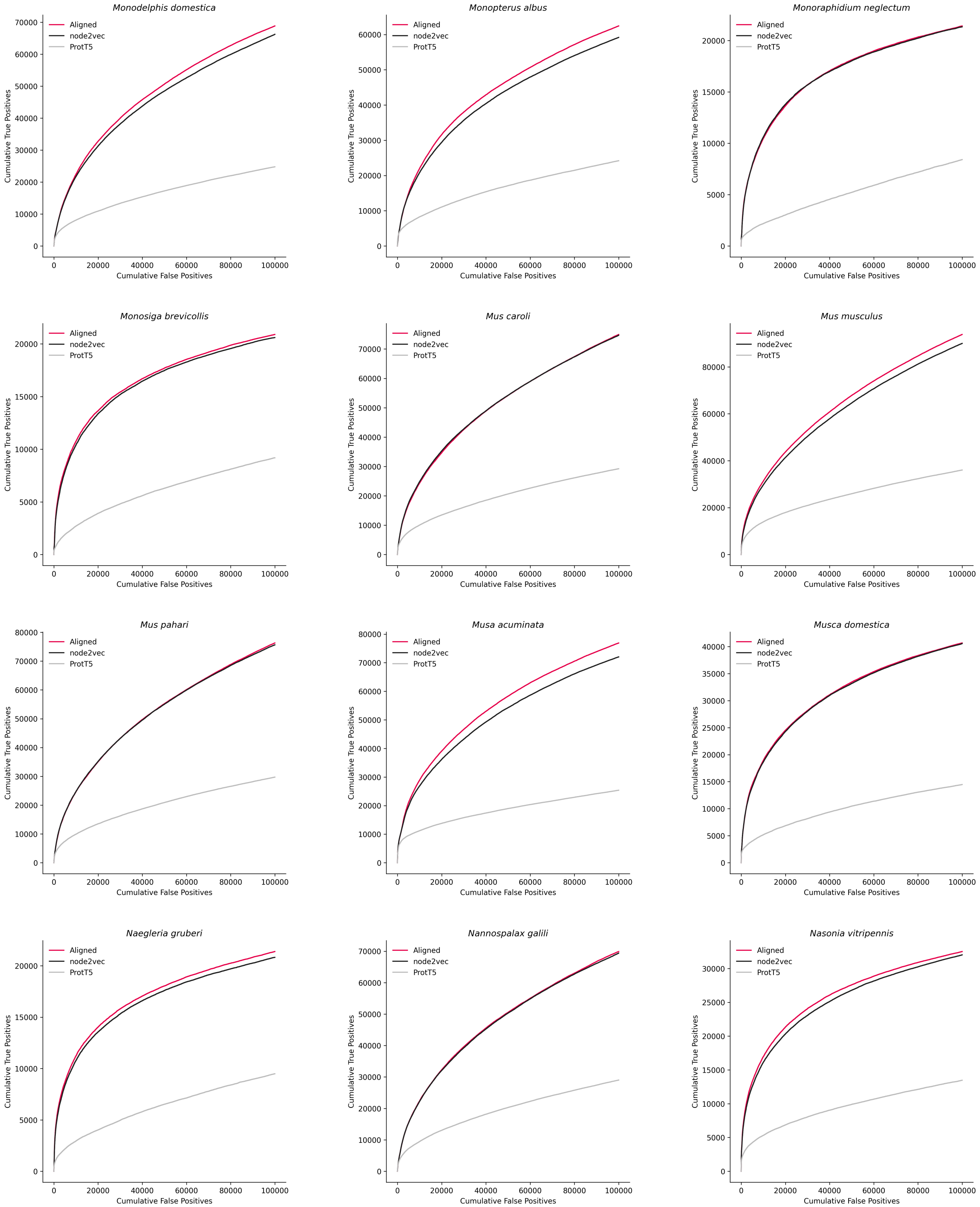

Monodelphis domestica
Monopterus albus
Monoraphidium neglectum
Monosiga brevicollis
Mus caroli
Mus musculus
Mus pahari
Musa acuminata
Musca domestica
Naegleria gruberi
Nannospalax galili
Nasonia vitripennis
Aligned
node2vec
ProtT5
Cumulative True Positives
Cumulative False Positives

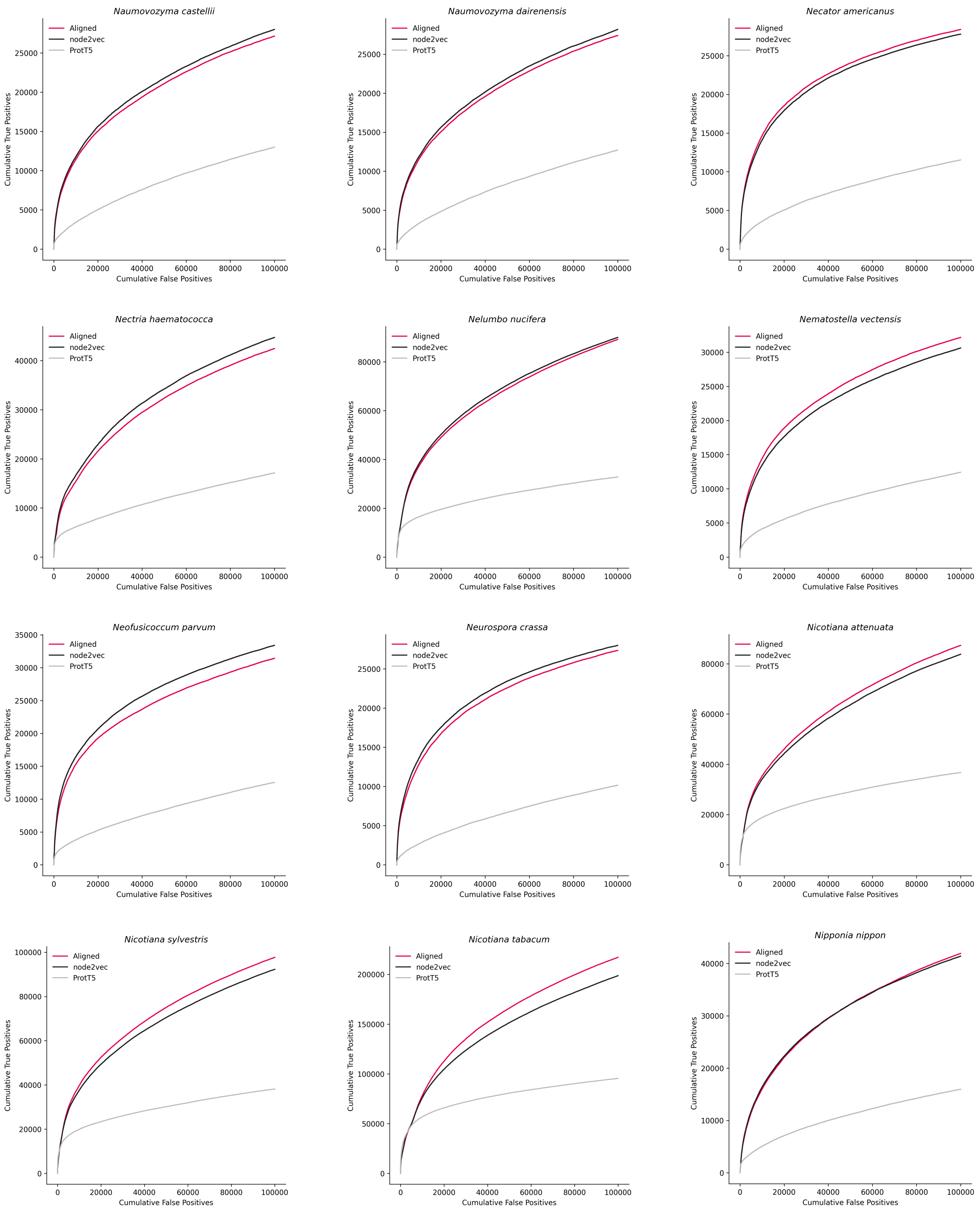
Naumovozyma castellii
Naumovozyma dairenensis
Necator americanus
Nectria haematococca
Nelumbo nucifera
Nematostella vectensis
Neofusicoccum parvum
Neurospora crassa
Nicotiana attenuata
Nicotiana sylvestris
Nicotiana tabacum
Nipponia nippon
Aligned
node2vec
ProtT5
Cumulative True Positives
Cumulative False Positives

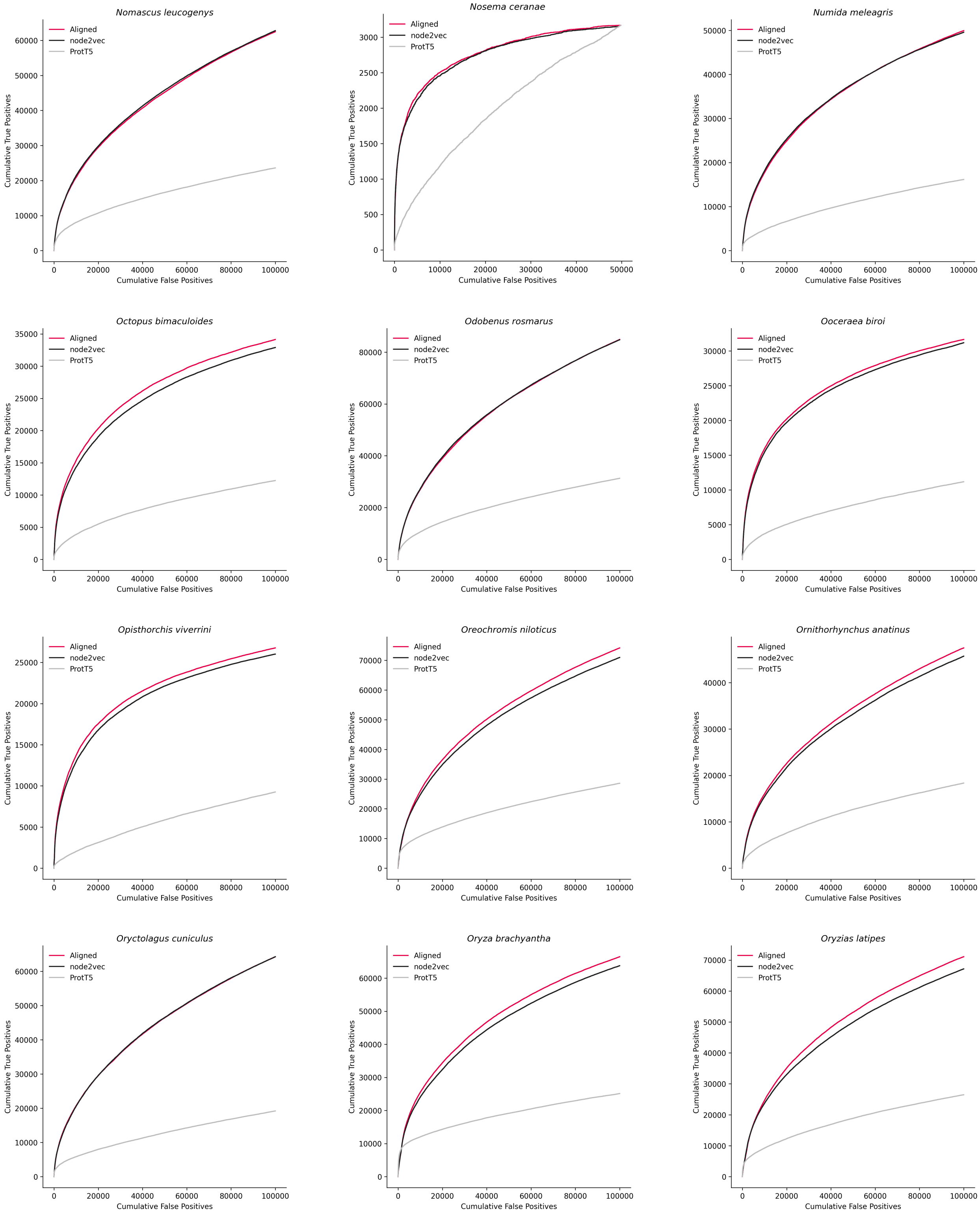
Nomascus leucogenys
Nosema ceranae
Numida meleagris
Octopus bimaculoides
Odobenus rosmarus
Ooceraea biroi
Opisthorchis viverrini
Oreochromis niloticus
Ornithorhynchus anatinus
Oryctolagus cuniculus
Oryza brachyantha
Oryzias latipes
Aligned
node2vec
ProtT5
Cumulative True Positives
Cumulative False Positives

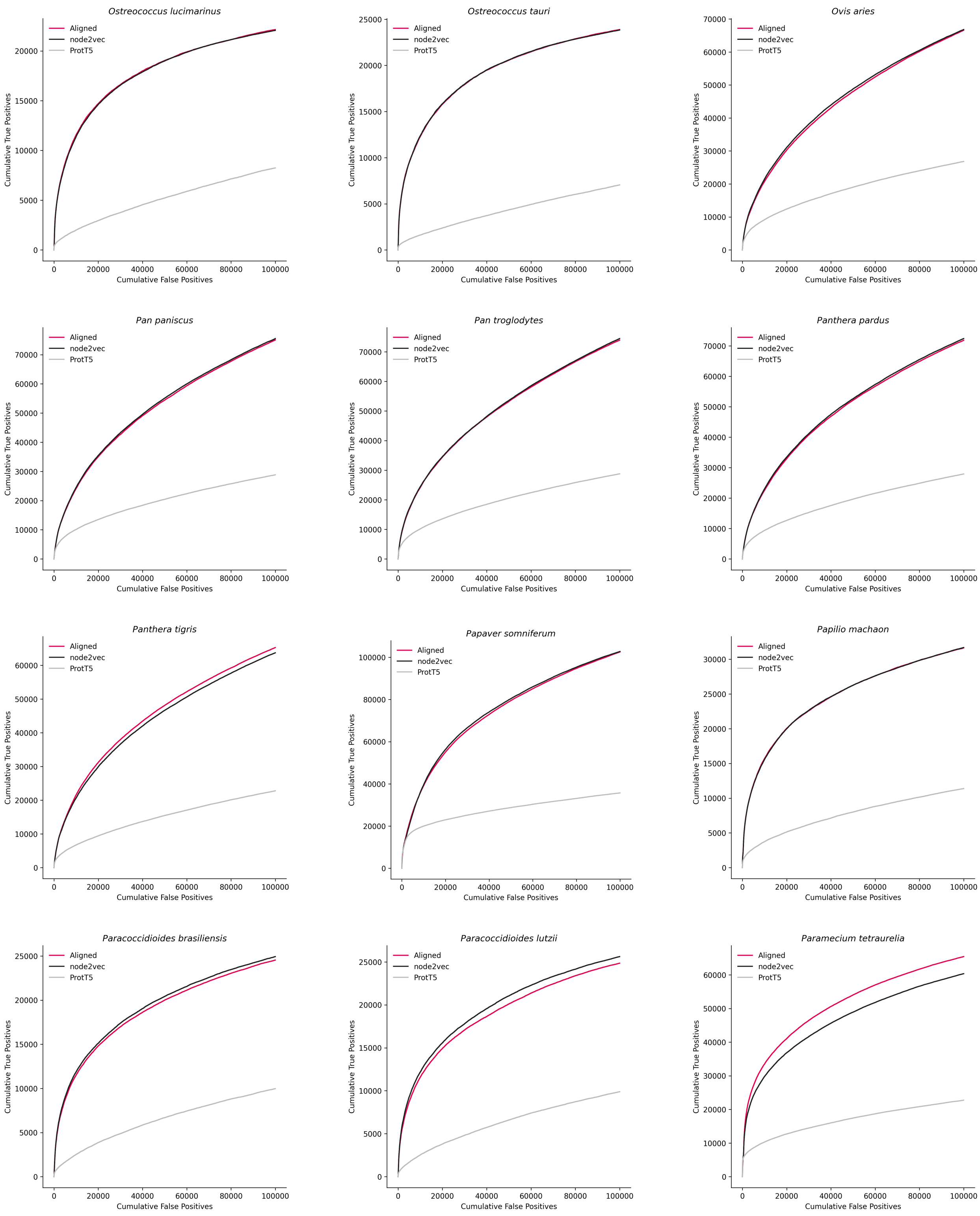
Ostreococcus lucimarinus
Ostreococcus tauri
Ovis aries
Pan paniscus
Pan troglodytes
Panthera pardus
Panthera tigris
Papaver somniferum
Papilio machaon
Paracoccidioides brasiliensis
Paracoccidioides lutzii
Paramecium tetraurelia
Aligned
node2vec
ProtT5
Cumulative True Positives
Cumulative False Positives

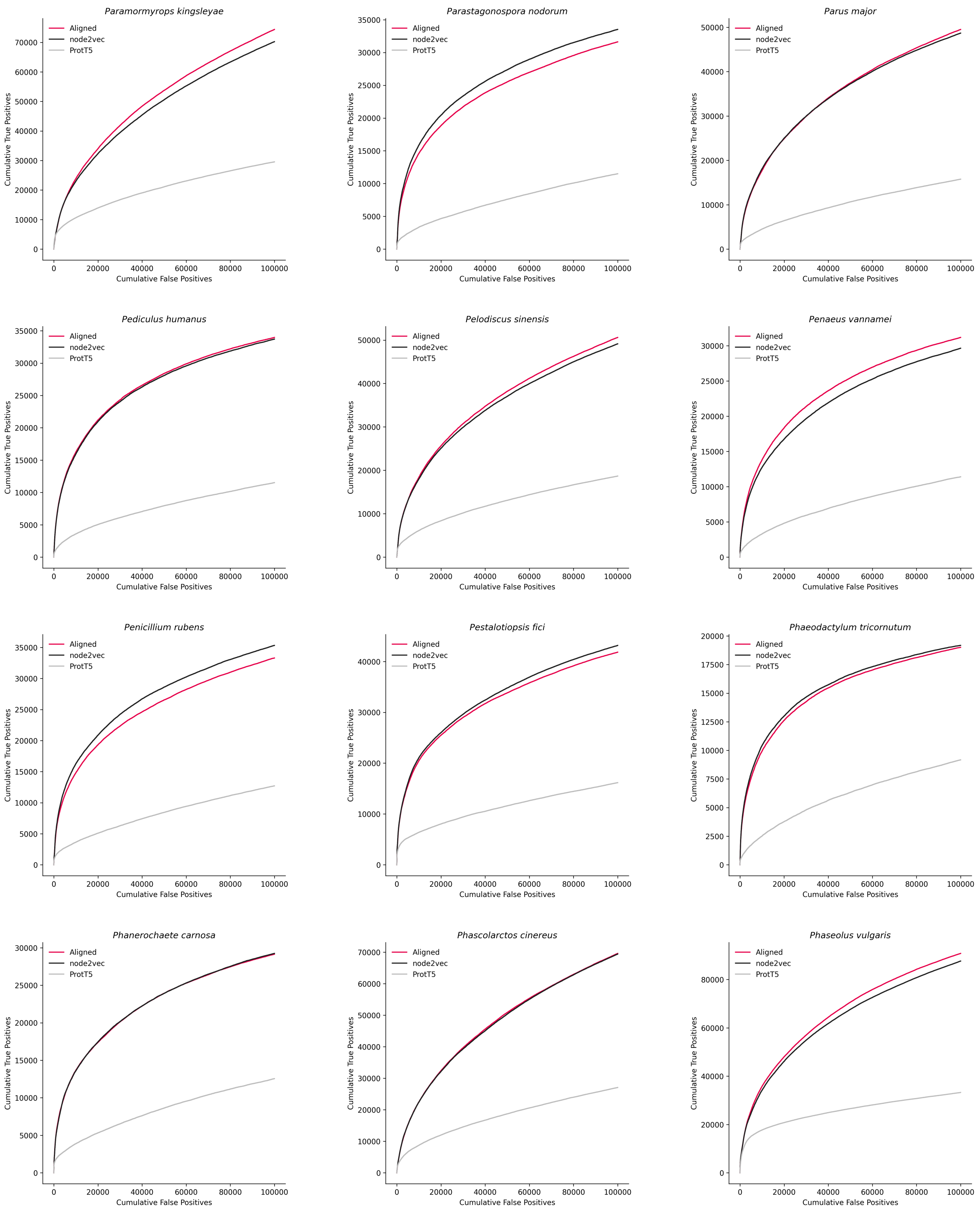
Paramormyrops kingsleyae
Parastagonospora nodorum
Parus major
Pediculus humanus
Pelodiscus sinensis
Penaeus vannamei
Penicillium rubens
Pestalotiopsis fici
Phaeodactylum tricornutum
Phanerochaete carnosa
Phascolarctos cinereus
Phaseolus vulgaris
Aligned
node2vec
ProtT5
Cumulative True Positives
Cumulative False Positives
0
20000
40000
60000
80000
100000

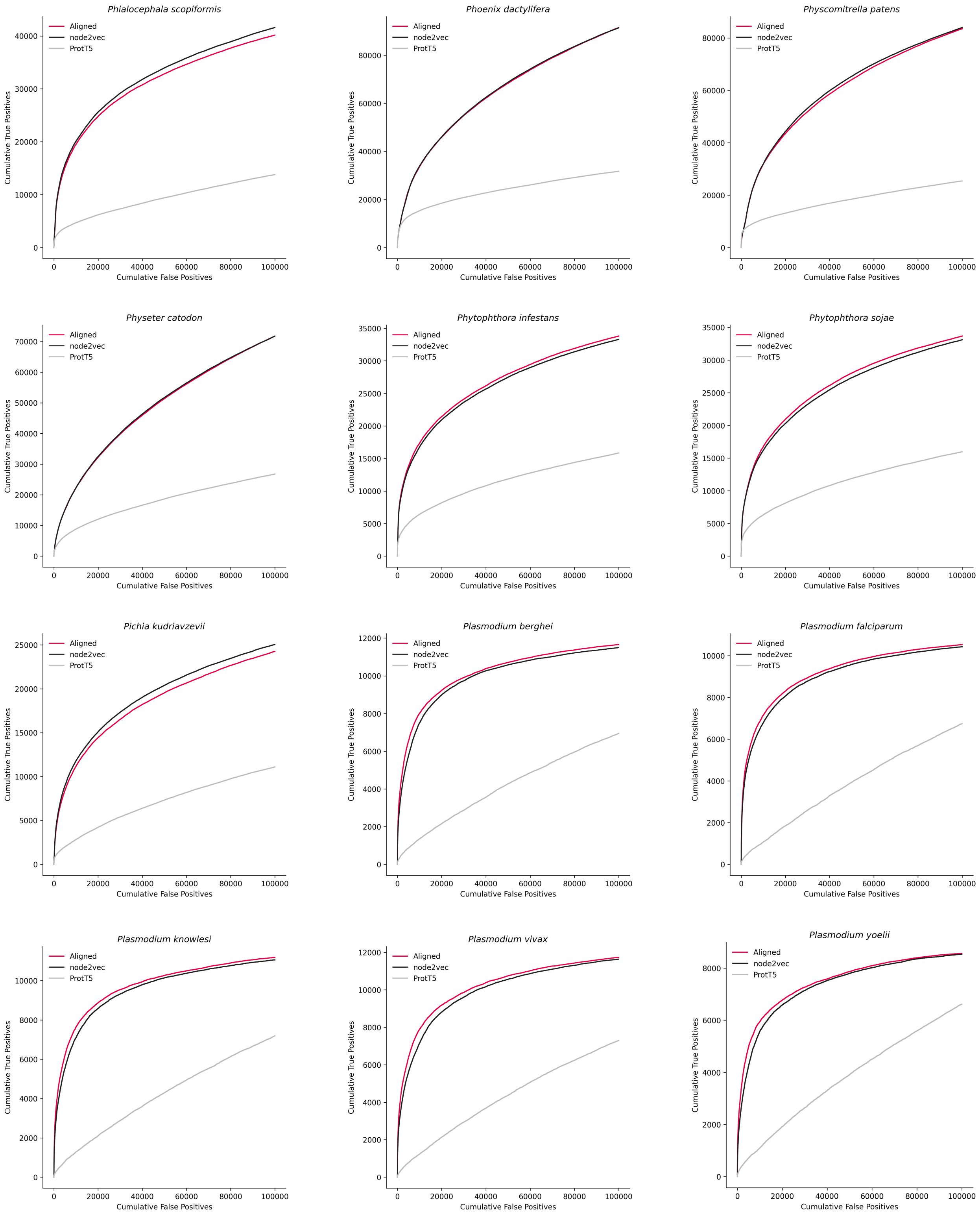
Phialocephala scopiformis
Phoenix dactylifera
Physcomitrella patens
Physeter catodon
Phytophthora infestans
Phytophthora sojae
Pichia kudriavzevii
Plasmodium berghei
Plasmodium falciparum
Plasmodium knowlesi
Plasmodium vivax
Plasmodium yoelii
Aligned
node2vec
ProtT5
Cumulative True Positives
Cumulative False Positives

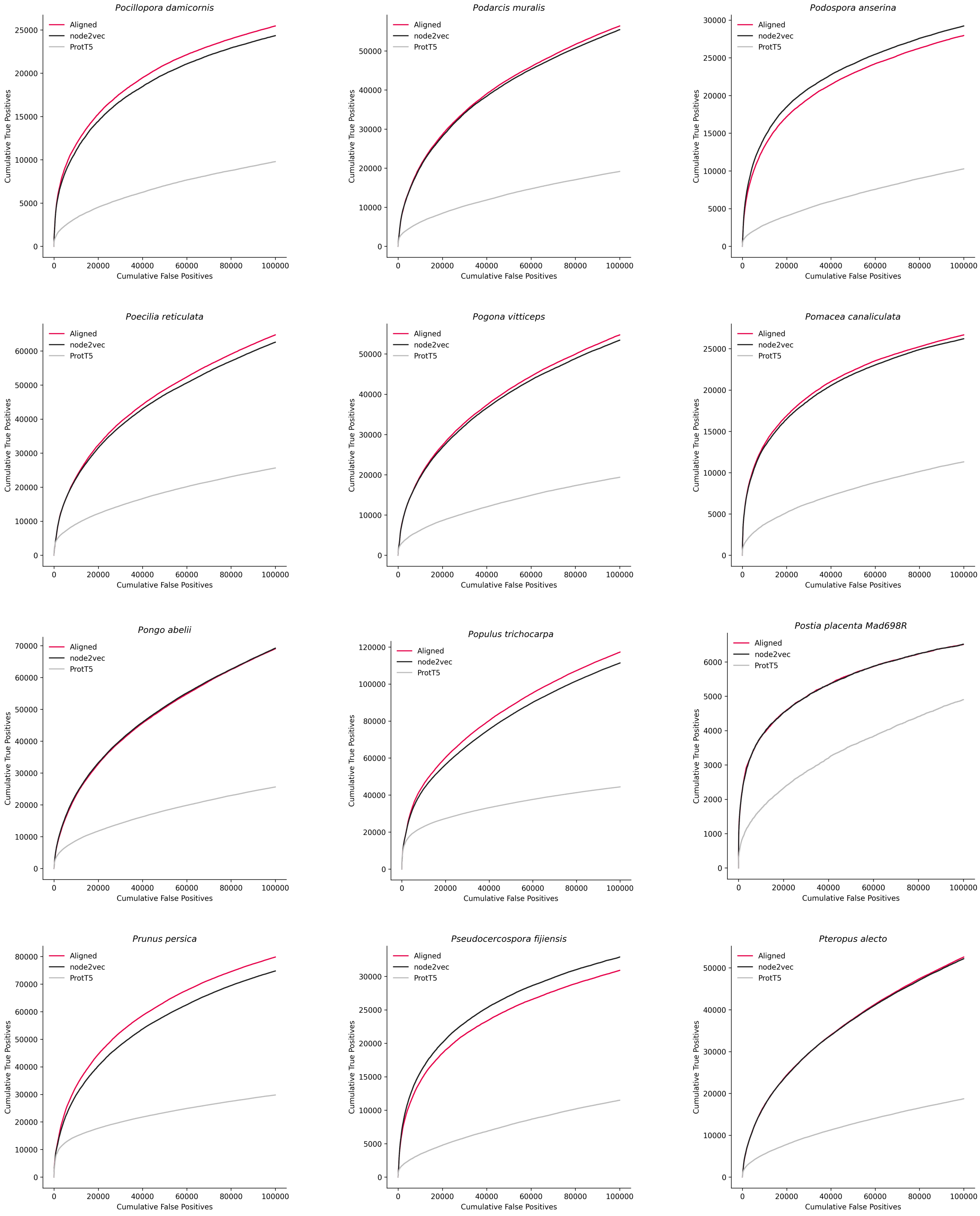
Pocillopora damicornis
Podarcis muralis
Podospora anserina
Poecilia reticulata
Pogona vitticeps
Pomacea canaliculata
Pongo abelii
Populus trichocarpa
Postia placenta Mad698R
Prunus persica
Pseudocercospora fijiensis
Pteropus alecto
Aligned
node2vec
ProtT5
Cumulative True Positives
Cumulative False Positives

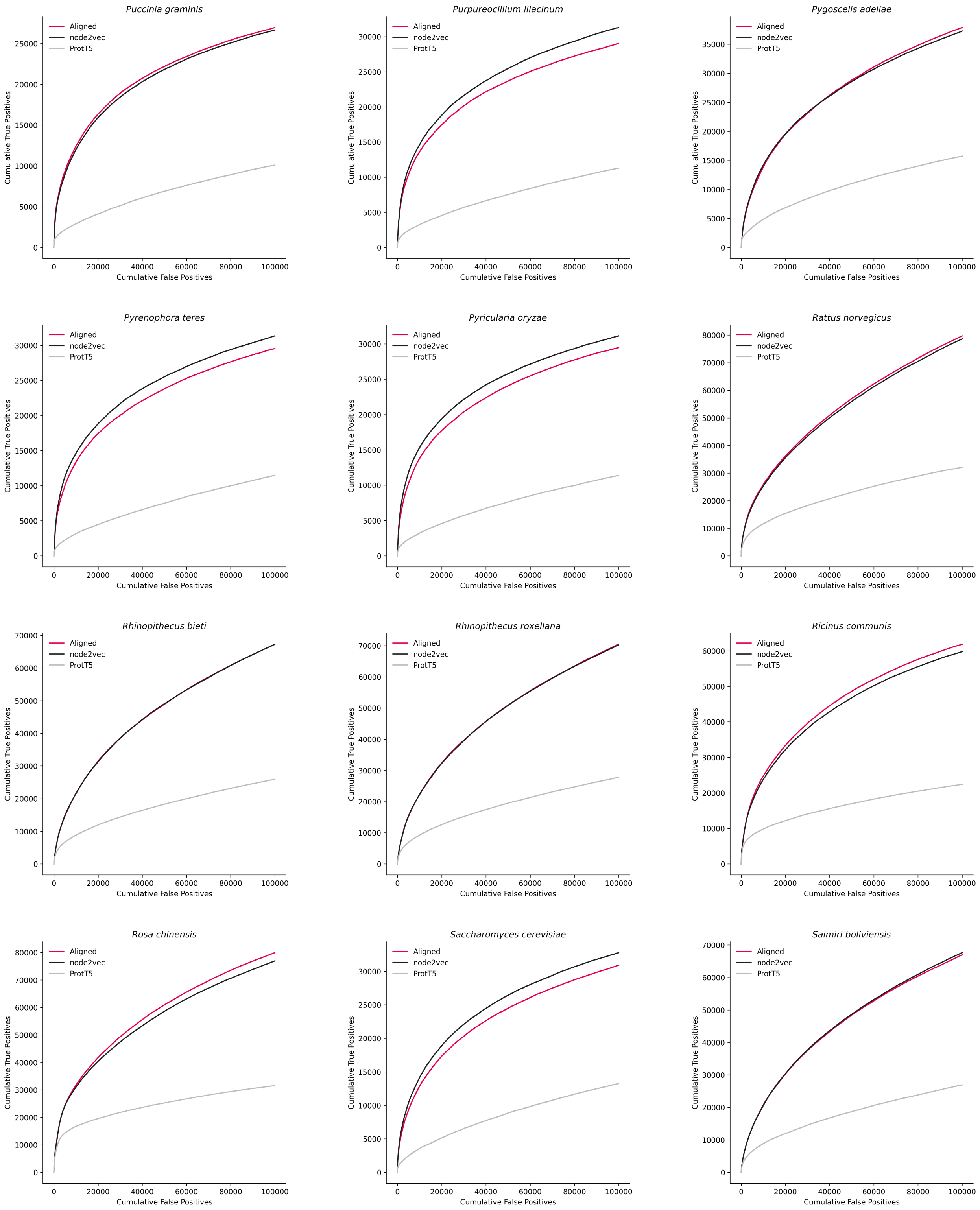
Puccinia graminis
Purpureocillium lilacinum
Pygoscelis adeliae
Pyrenophora teres
Pyricularia oryzae
Rattus norvegicus
Rhinopithecus bieti
Rhinopithecus roxellana
Ricinus communis
Rosa chinensis
Saccharomyces cerevisiae
Saimiri boliviensis
Aligned
node2vec
ProtT5
Cumulative True Positives
Cumulative False Positives

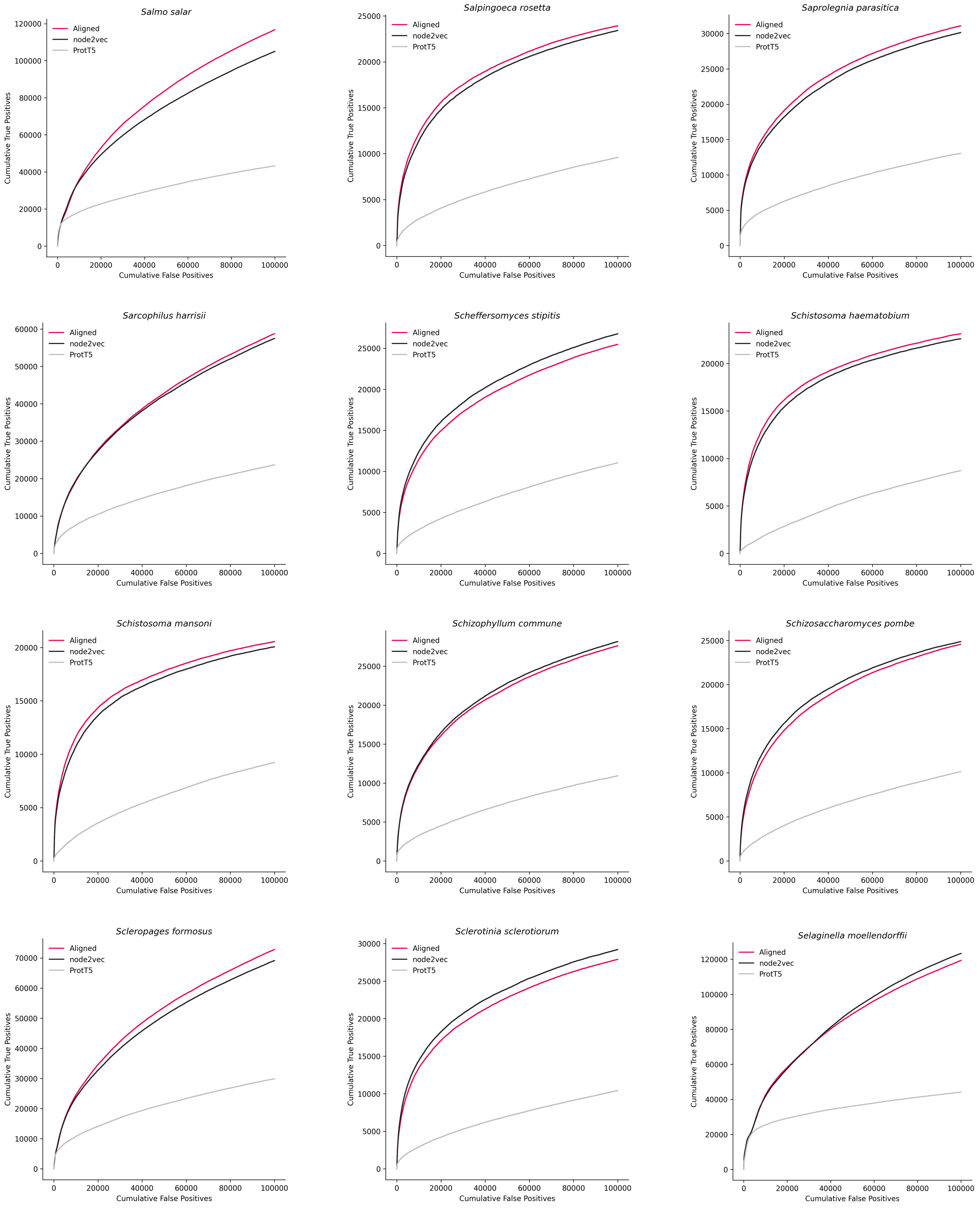
Salmo salar
Salpingoeca rosetta
Saprolegnia parasitica
Sarcophilus harrisii
Scheffersomyces stipitis
Schistosoma haematobium
Schistosoma mansoni
Schizophyllum commune
Schizosaccharomyces pombe
Scleropages formosus
Sclerotinia sclerotiorum
Selaginella moellendorffii
Aligned
node2vec
ProtT5
Cumulative True Positives
Cumulative False Positives

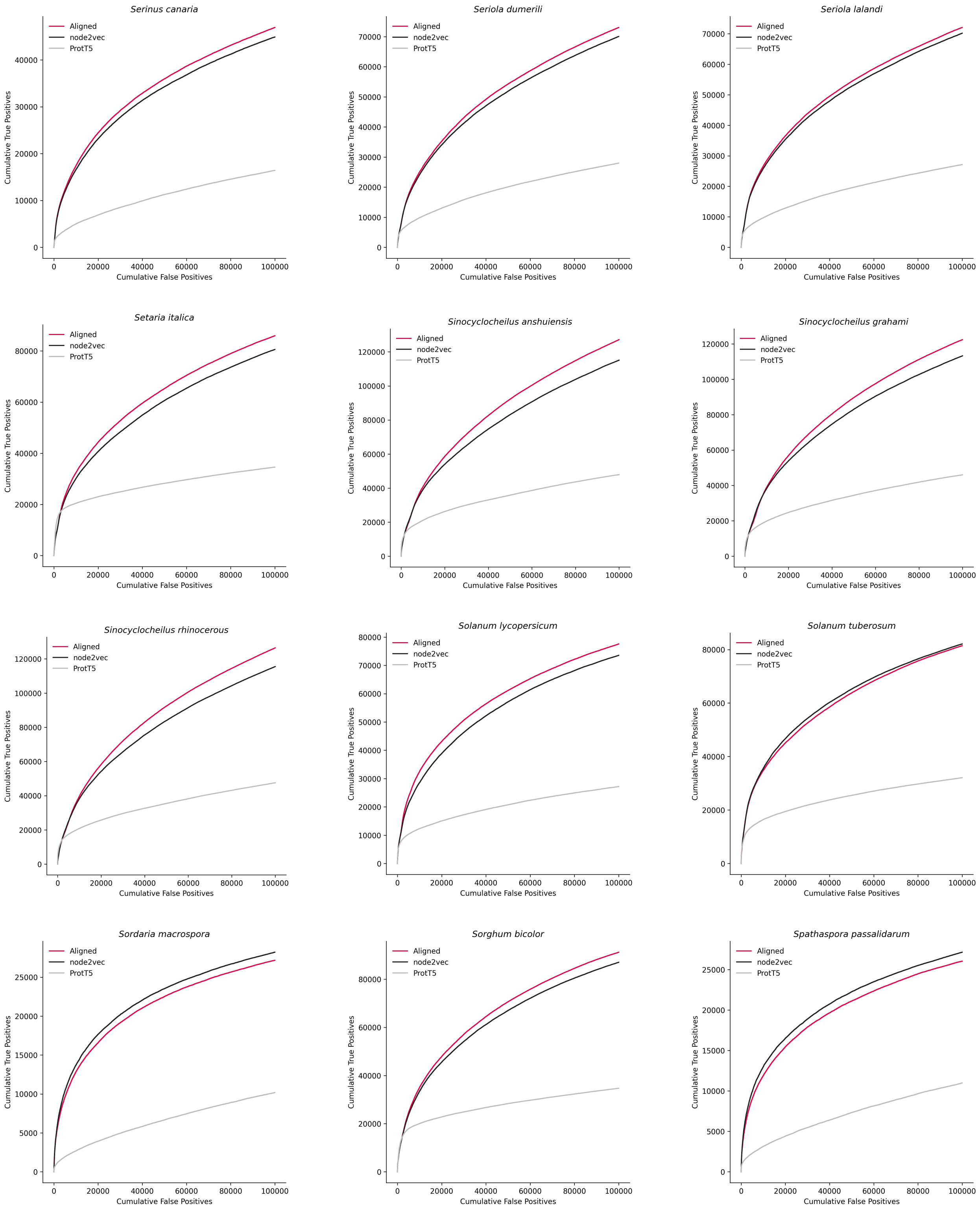
Serinus canaria
Seriola dumerili
Seriola lalandi
Setaria italica
Sinocyclocheilus anshuiensis
Sinocyclocheilus grahami
Sinocyclocheilus rhinocerous
Solanum lycopersicum
Solanum tuberosum
Sordaria macrospora
Sorghum bicolor
Spathaspora passalidarum
Aligned
node2vec
ProtT5
Cumulative True Positives
Cumulative False Positives

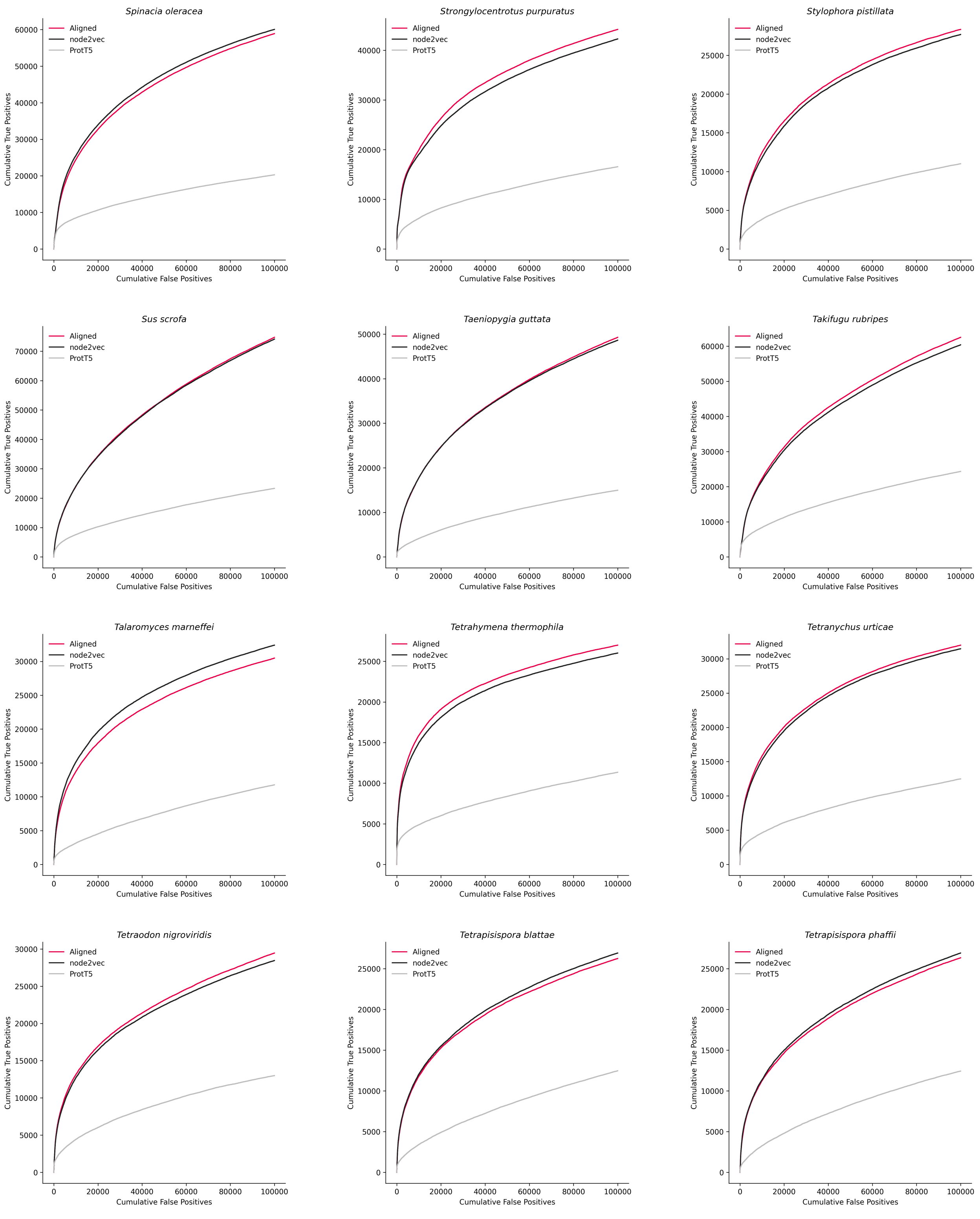

Spinacia oleracea
Strongylocentrotus purpuratus
Stylophora pistillata
Sus scrofa
Taeniopygia guttata
Takifugu rubripes
Talaromyces marneffei
Tetrahymena thermophila
Tetranychus urticae
Tetraodon nigroviridis
Tetrapisispora blattae
Tetrapisispora phaffii
Aligned
node2vec
ProtT5
Cumulative True Positives
Cumulative False Positives

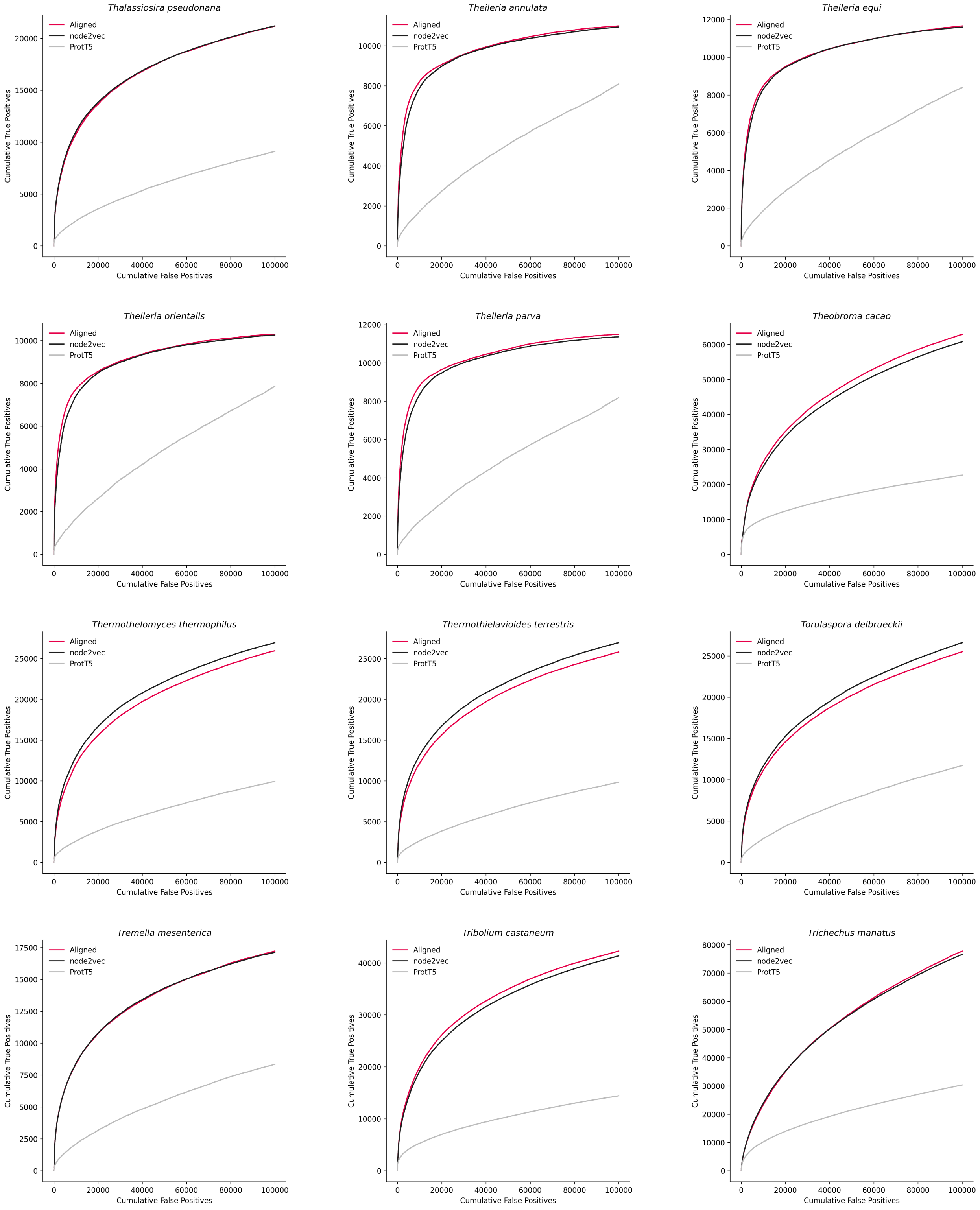
Thalassiosira pseudonana
Theileria annulata
Theileria equi
Theileria orientalis
Theileria parva
Theobroma cacao
Thermothelomyces thermophilus
Thermothielavioides terrestris
Torulaspora delbrueckii
Tremella mesenterica
Tribolium castaneum
Trichechus manatus
Aligned
node2vec
ProtT5
Cumulative True Positives
Cumulative False Positives

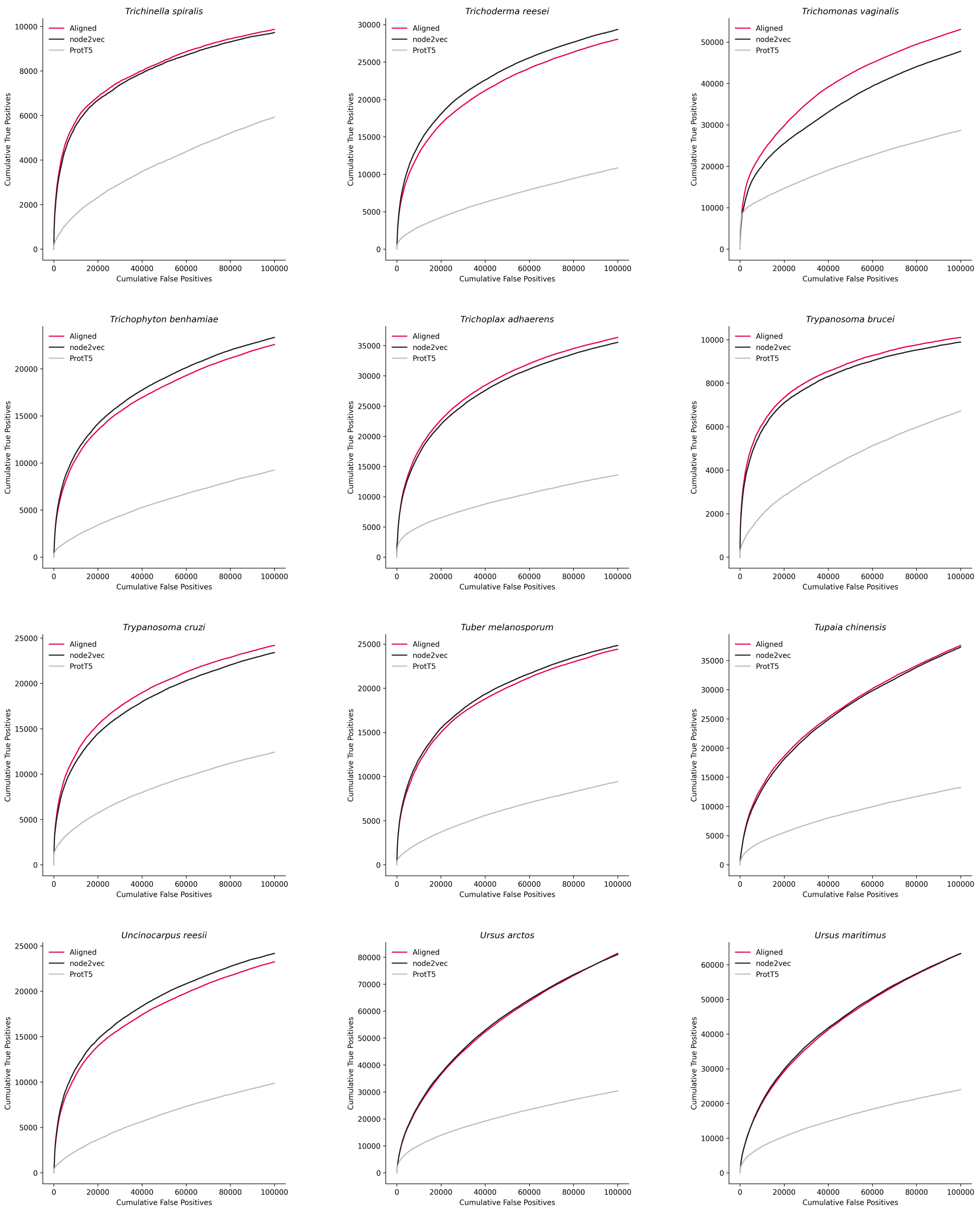
Trichinella spiralis
Trichoderma reesei
Trichomonas vaginalis
Trichophyton benhamiae
Trichoplax adhaerens
Trypanosoma brucei
Trypanosoma cruzi
Tuber melanosporum
Tupaia chinensis
Uncinocarpus reesii
Ursus arctos
Ursus maritimus
Aligned
node2vec
ProtT5
Cumulative True Positives
Cumulative False Positives

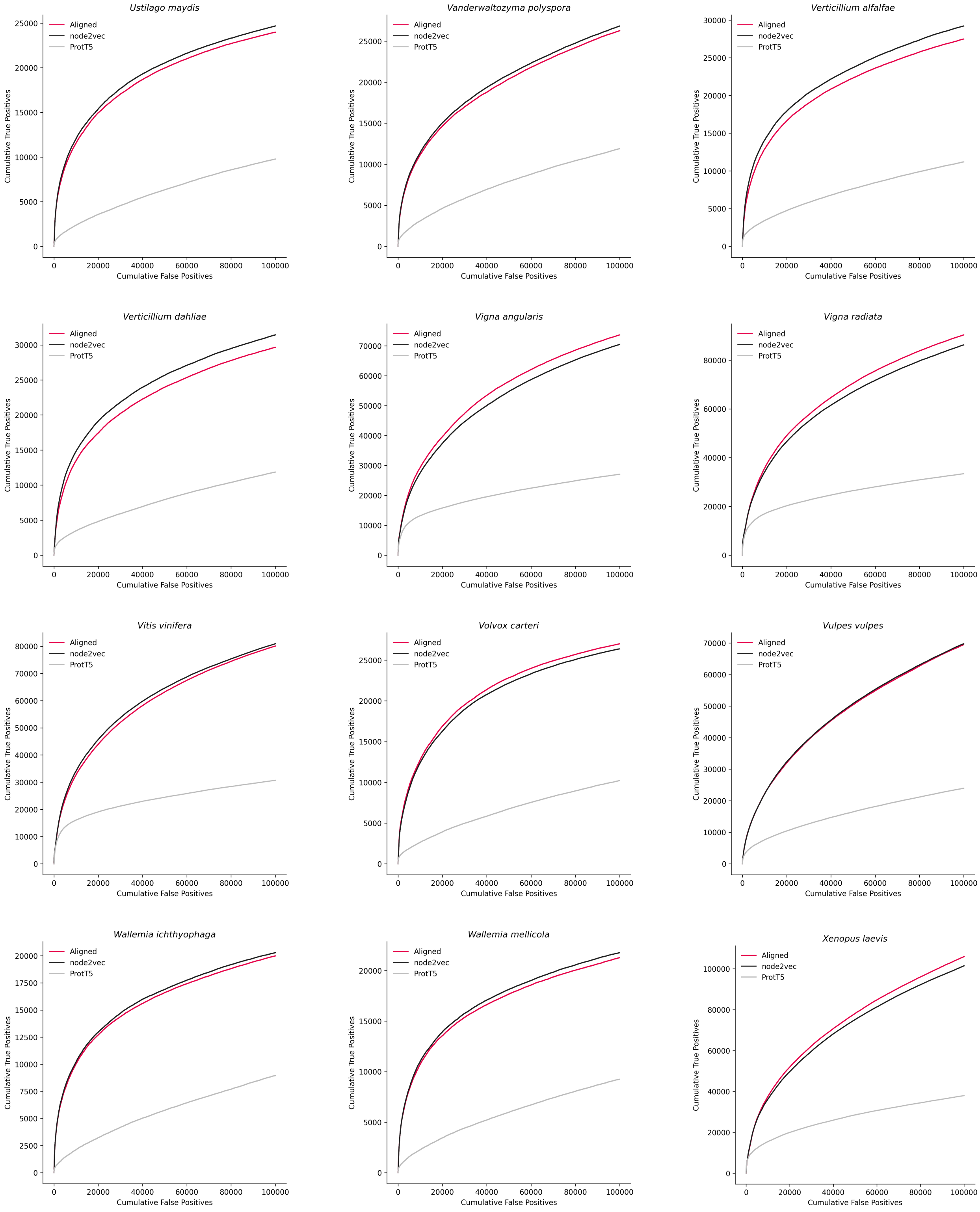
Ustilago maydis
Vanderwaltozyma polyspora
Verticillium alfalfae
Verticillium dahliae
Vigna angularis
Vigna radiata
Vitis vinifera
Volvox carteri
Vulpes vulpes
Wallemia ichthyophaga
Wallemia mellicola
Xenopus laevis
Aligned
node2vec
ProtT5
Cumulative True Positives
Cumulative False Positives

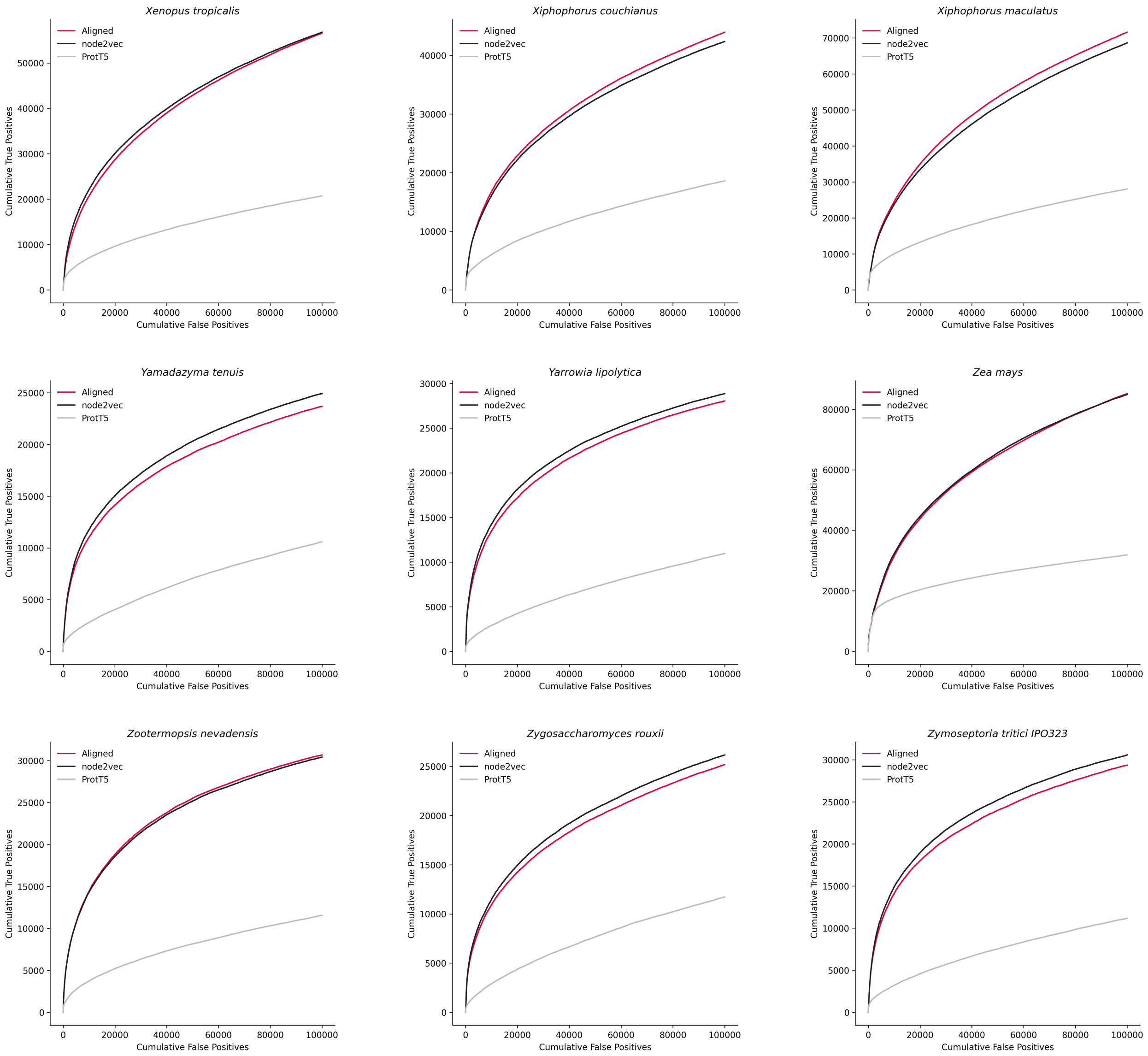
Xenopus tropicalis
Xiphophorus couchianus
Xiphophorus maculatus
Yamadazyma tenuis
Yarrowia lipolytica
Zea mays
Zootermopsis nevadensis
Zygosaccharomyces rouxii
Zymoseptoria tritici IPO323
Aligned
node2vec
ProtT5
Cumulative True Positives
Cumulative False Positives
